# *Ocimum* metabolomics in response to abiotic stresses: Cold, flood, drought and salinity

**DOI:** 10.1371/journal.pone.0210903

**Published:** 2019-02-06

**Authors:** Shubhra Rastogi, Saumya Shah, Ritesh Kumar, Divya Vashisth, Md Qussen Akhtar, Ajay Kumar, Upendra Nath Dwivedi, Ajit Kumar Shasany

**Affiliations:** 1 Department of Biochemistry, University of Lucknow, Lucknow, Uttar Pradesh, India; 2 Biotechnology Division, CSIR-Central Institute of Medicinal and Aromatic Plants, Lucknow, Uttar Pradesh, India; Louisiana State University, UNITED STATES

## Abstract

*Ocimum tenuiflorum* is a widely used medicinal plant since ancient times and still continues to be irreplaceable due to its properties. The plant has been explored chemically and pharmacologically, however, the molecular studies have been started lately. In an attempt to get a comprehensive overview of the abiotic stress response in *O*. *tenuiflorum*, *de novo* transcriptome sequencing of plant leaves under the cold, drought, flood and salinity stresses was carried out. A comparative differential gene expression (DGE) study was carried out between the common transcripts in each stress with respect to the control. KEGG pathway analysis and gene ontology (GO) enrichment studies exhibited several modifications in metabolic pathways as the result of four abiotic stresses. Besides this, a comparative metabolite profiling of stress and control samples was performed. Among the cold, drought, flood and salinity stresses, the plant was most susceptible to the cold stress. Severe treatments of all these abiotic stresses also decreased eugenol which is the main secondary metabolite present in the *O*. *tenuiflorum* plant. This investigation presents a comprehensive analysis of the abiotic stress effects in *O*. *tenuiflorum*. Current study provides an insight to the status of pathway genes’ expression that help synthesizing economically valuable phenylpropanoids and terpenoids related to the adaptation of the plant. This study identified several putative abiotic stress tolerant genes which can be utilized to either breed stress tolerant *O*. *tenuiflorum* through pyramiding or generating transgenic plants.

## Introduction

Abiotic stresses like- drought, flood, salinity and cold are the environmental factors which hold an adverse impact over the plants’ growth and productivity. In response, plants have developed numerous physiological and biochemical homeostatic balance to attain tolerance and adaptation against such stresses [1]. These remedies include molecular rearrangements at some stage in gene expression initiating from regulation at transcriptional level leading to processing of mRNA, post-transcriptional level or modification of protein. Under these abiotic stresses, plants exhibit precise transcriptional regulation affecting its transcriptome [2–5]. Abiotic stresses affect differently plant to plant, like drought stress in potato reduces the economic yield of plant by 13% while, in case of rice, maize and common beans yield reduction ranges from 24 to 92% [6].

Cold stress is one of the most damaging abiotic stresses. Transcriptome and the metabolism of the plant are severely affected by the cold stress which may be due to the direct inhibition of metabolic enzymes at cold temperatures as well as reprogrammed gene expression [7]. As reported earlier, cold stress raises the production of phenolics and facilitates their assimilation into the cell wall as either lignin or suberin [8]. Whereas drought stress due to water deficit conditions is generally associated with elevated temperatures and solar heat. Though the tolerance to drought stress is observed in all plants, while the extent of tolerance differs from species to species [9]. On the contrary, the condition of flood also imposes a critical problem in terms of stress which is caused by oxygen insufficiency leading to wilting though the root is surrounded by surplus water. This condition subsequently influences nutrient uptake by reducing the potassium/ sodium ion uptake inhibiting the translocation of potassium ion to the plant shoot [10,11]. The state of low oxygen concentrations or hypoxia in soils is due to the low oxygen solubility in water [12]. On the other hand, salinity causes cell dehydration by osmotic imbalance and cytoplasmic water elimination and this leads to drop in vacuolar and cytosolic volumes. Because of salinity stress, there may be accretion or reduction of a particular secondary metabolite *in planta* [13] like anthocyanins show an increase in content in response to salt stress [14]. On the contrary, salt-sensitive species exhibit a decreased level of anthocyanin under salinity stress [15].

**Fig 1:**
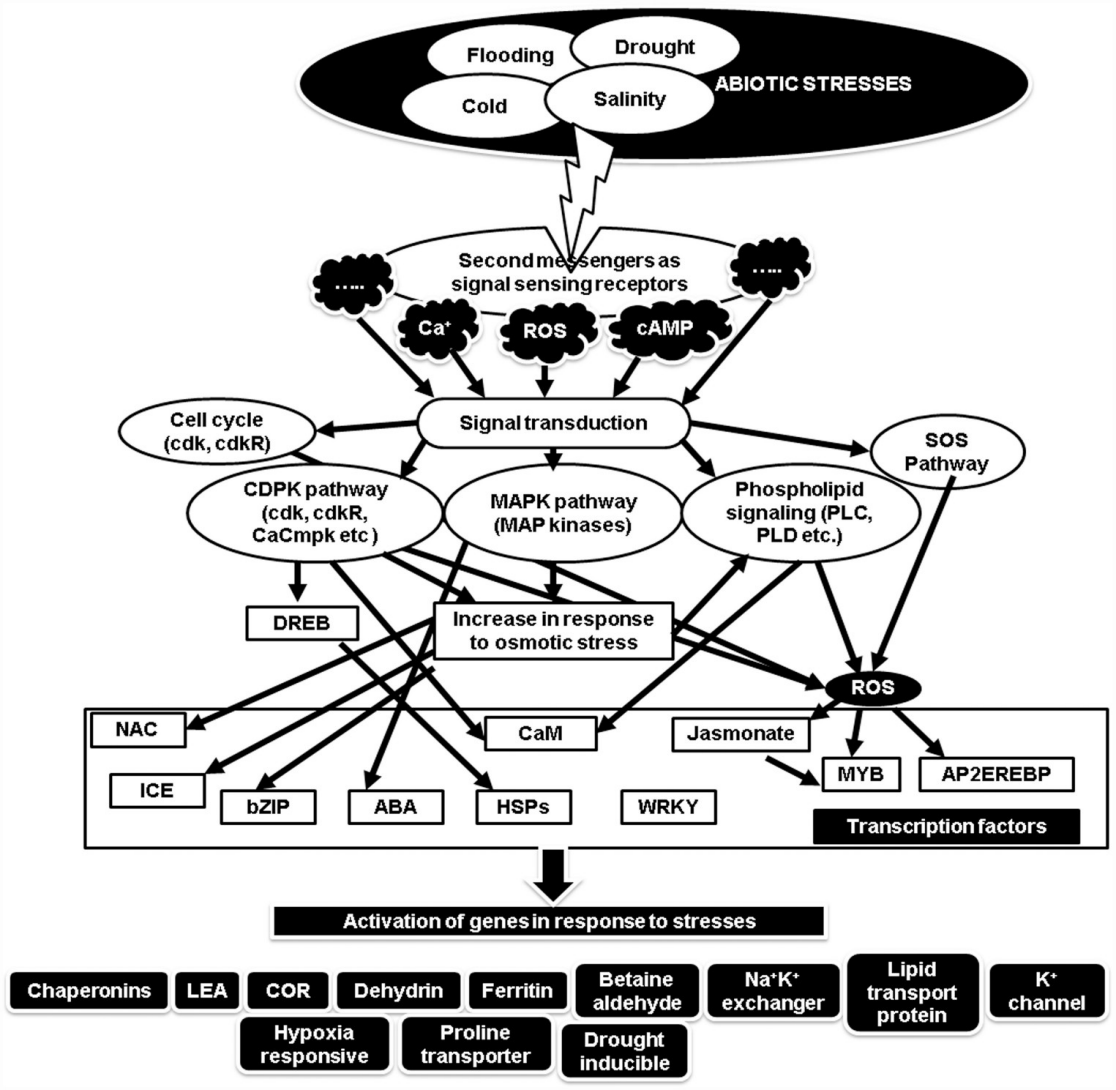
A Schematic overview of the activation of the genes in response to four abiotic stresses (Cold, Flood, Drought and Salinity). **Abbreviations**: ROS (Reactive Oxygen species), cAMP (cyclic adenosine monophosphate), *CDPK* (Calcium dependent protein kinases), *cdk* (cyclin dependent kinase), *cdkR* (cyclin dependent kinases regulatory), *CaCmpk* (Ca calmodulin dependent protein kinases), *MAPK* (mitogen activated protein kinases, *PLC* (phospholipase C), *SOS* (salt overlay stress), *PLD* (phospholipase D), *NAC* (*NAM* (no apical meristem), *ArabidopsisATAF1/2* and *CUC2*), *ICE* (inducer of CBF expression 1), *bZIP* (basic Leucine Zipper Domain), ABA (abscisic acid), CaM (calmodulin), *AP2EREBP* (*AP2* (*APETALA2*) and *EREBP*s (ethylene-responsive element binding proteins), *LEA* (late embryogenesis abundant), and *COR* (cold responsive).

Previously, immense research has been carried out in an effort to understand the stress tolerance mechanism in plants. Significant candidate proteins, genes and pathways have been recognized utilising *Omics* approaches referring to huge sets of biological molecules [16]. Gen*Omics* imply for identification of genes, transcript*Omics* for mRNA, proteomics for protein, and metabol*Omics* for metabolites identification [16]. Despite the fact that several molecular mechanisms related to stress have been revealed in other plants [7–13], however, complete understanding in *O*. *tenuiflorum* is still lacking. In this regard, transcriptomic strategies have opened the avenues in comprehending plants’ response against abiotic stresses [17–18]. A schematic overview of the process of abiotic stress and activation of the genes in response to these stresses has been provided in Fig 1.

*Ocimum tenuiflorum* (2n = 16) is one of an important medicinal and aromatic plant belonging to the family lamiaceae which is grown widely worldwide [19]. Lately, *O*. *tenuiflorum* has been investigated for its genome information and the secondary metabolic pathway related genes [20–22]. However, in the current scenario of climate change, the molecular studies of the plant against the major abiotic stresses still remain elusive. It is well established that *O*. *tenuiflorum* is most susceptible to cold stress but the effect of drought, flooding and salinity stresses on this plant has yet not been investigated. Considering all these above points, the present study was executed to get an insight of the transcriptomic changes occurring in the *O*. *tenuiflorum* plant under cold, drought, flood and salinity stresses, separately. Transcripts common as well as exclusive in each of the abiotic stresses were also analysed.

## Materials and methods

### Abiotic stress treatment to the plants and their sampling

Seeds of ‘CIM-AYU’ variety of *O*. *tenuiflorum* were collected from National Gene Bank of CSIR-Central Institute of Medicinal and Aromatic Plants, India. The pot experiment was conducted at Glass-house facility of CIMAP, Lucknow, U.P. India (26.8946° N, 80.9815° E). The seeds were sown in earthen pots (3000 cm^3^) in the glass house providing a photoperiod of 14 h natural light/10 h dark, 85% relative humidity and temperature of 250C/ 200C (day/ night). Two months old plants after germination were used for the stress treatment. Each treatment of flood, drought, salt and cold was provided to 10 plants along with control of same developmental stage and pooled samples were collected for library preparation. Stress treatments were given in the soil (sandy loam type) mixed with vermi-compost in the ratio of 3:1. Conditions of all the stress treatments were optimized with different parameters as shown in S1 Fig. and only single sample of each abiotic stress exhibiting severe stress were chosen for transcriptome sequencing. Flood treatment was given to the plants by constantly maintaining the water level 0.5 inches above the soil in the earthen pots [23]. 0.5 inch water level was maintained in flood treatment plants so as to avoid the water loss due to evaporation. The treatment was continued up to 30 days and thereafter the samples were collected. In case of drought stress, plants were maintained at 20% field capacity for 30 days as described by Chauhan and Abugho [24]. *O*. *tenuiflorum* plants were subjected to drought stress with 20%, 40% and 60% field capacities and out of this 20% field capacity sample was selected for the transcriptome sequencing. Salinity stress was given in 5 concentrations *viz*. 25mM, 50mM, 75mM, 100mM and 125mM NaCl by irrigating the test plant pots with 100ml of NaCl solution [25] constantly for 10 days and at every alternate day during the complete treatment cycle of 30 days. To prevent the plants from osmotic shock, treatment was started with lower concentrations, gradually increasing the concentration daily, until each group. However, sample of 125 mM salt with severe stress was selected for transcriptome sequencing. Cold treatment was given to the plants by incubating them in a cold room at 40C by maintaining the optimal light required by the plant [26]. The cold treatment sample was collected after 72 hours as standardized by the prior experiments because exceeding the incubation time leads to plant death.

Common control plants were kept in normal conditions with regular watering to maintain the soil moisture up to the water holding capacity of the soil. Leaf samples of drought, flood and salinity stress treatments were harvested after 30 days while the cold treatment was provided at the third last day such that all the controls had the same time point for sample collection. Leaf samples from the whole plant were collected and pooled for sequencing in triplicates as well as other molecular and metabolite analysis. Samples were stored in RNA later (Sigma Aldrich) at -800C until use [27].

### Total RNA isolation and its quantitative and qualitative analysis

Total RNA was isolated from the leaf samples by ZR Plant RNA Miniprep (Zymo Research, Irvine, CA, USA) as per supplier’s protocol [28]. Qualitative analysis of the isolated RNA was carried out using 1% Denaturing RNA agarose gel and was quantified using NanoDrop.

### Library preparation, transcriptome sequencing and assembly

Illumina NextSeq paired end (PE) libraries were prepared from the quality check passed RNA samples *via* illumina TruSeq stranded mRNA sample preparation kit (Part # 15008136; Rev. A; Nov 2010) following the protocol as stated in “TruSeq RNA Sample Preparation Guide” [29]. The PCR enriched libraries were analysed in 4200 Tape Station system (Agilent Technologies) with High sensitivity D1000 Screen tape as per the makers’ instructions. PE illumina libraries were loaded onto NextSeq 500 for cluster generation and sequencing in both directions, applying 2x75 bp chemistry. The raw sequencing data was then processed to get the high quality clean reads using Trimmomatic v0.35 [30] to get rid of adapter sequences, confusing reads (unidentified nucleotides bigger than 5%), and the bad quality reads (having quality threshold (QV) more than 10% and phred score less than 20). A minimum length of 50 nucleotides after trimming was applied. *De novo* assembly was then carried out with these high quality (QV<20), paired-end reads using Trinity RNA-Seq assembler set at default parameters. Great quantity of misassembled transcripts and erroneous transcripts may appear during the sequence assembly; hence, all high quality sequence reads were mapped back to the corresponding assembled transcripts using BWA v0.7.12 [31] for validation of transcript. The non-redundant transcripts were further clustered together using CD-HIT-EST-4.5.5 [32] at 95% identity and query coverage. These are called the unigenes (the sequences which could no longer be extended). NGS sequencing data generated in the current investigation is submitted to NCBI SRA (Short Read Archive). The bioproject IDassigned is: (PRJNA390220) https://www.ncbi.nlm.nih.gov/bioproject/PRJNA390220).

### Coding sequence prediction and functional annotation

The TransDecoder (http://transdecoder.github.io) was used to predict coding sequences (CDS) from the unigenes [33]. NCBI non redundant (NR) protein database was used to search the predicted CDSs with the help of Basic local alignment search tool (Blast X) (E value 1e-05). Blast2GO program was used to reveal the Gene ontology (GO) annotations of the CDSs. Further, the predicted CDSs were classified for their roles through GO assignments. Three criterions were followed to retrieve GO terms from the annotated CDSs in the course of GO mapping. First, accession IDs from the BlastX result were utilized to reclaim gene symbols or names, identified gene symbols or names were then explored in the species specific records of the gene product tables from GO database. Secondly, UniProt IDs were retrieved using the BlastX result accession IDs with the help of Protein Information Resource (PIR) which consists of databases like- Protein Sequence Database (PSD), UniProt, SwissProt, TrEMBL, RefSeq, GenPept and Protein Data Bank (PDB). Thirdly, dbxref and gene product tables of GO database were directly explored to search for the accession IDs. To identify the potential involvement of CDS of four abiotic stress samples (cold, flood, drought, salt) and control in biological pathways, CDS were mapped to standard pathways in KEGG database. The result of KEGG study consists of KEGG Orthology (KO) assignments and Corresponding EC numbers and metabolic pathways of the predicted CDS using KEGG automated annotation server KAAS [34]. A supplementary dataset for all the annotations of transcripts in control, cold, flood, drought and salt stresses have been provided in S1 Dataset.

### Differential gene expression

The good quality reads of each stress sample were mapped to their corresponding set of CDS using BWA aligner for read count calculation. The common hit accessions were recognized for differential gene expression studies on the basis of BLAST results against NR database. A negative binomial distribution model (DESeq1.8.1 package) (http://www-huber.embl.de/users/anders/DESeq/) [35] was employed to perform differential expression analysis. Method- blind, sharing mode- fit only and fit type- local parameters were used to estimate the dispersion values. Further on the basis of formula FC = Log2 (Treated/ Control) the log fold change (FC) was determined in order to classify the genes as up- or down-regulated. A comprehensive analysis of linkage hierarchical cluster was executed using multiple experiment viewer (MEV v4.8.1) [36] on best hundred differentially expressed genes. Heat map cluster shows the expression of genes. Level of expression is represented log2 ratio of gene abundance between control vs. cold, control vs. flood, control vs. drought, and control vs. salt. Log-transformed and normalized value of genes was used to construct the heat map on the basis of Pearson uncentered correlation distance as well as based on complete linkage method. Venn diagrams were prepared using Venny’s on-line [37]. A supplementary dataset for the DGE of Control vs. Cold, Control vs. Flood, Control vs. Drought, and Control vs. Salt with annotations have been provided in the S2, S3, S4 and S5 Datasets, respectively.

### Metabolite analysis and comparative profiling

Comparative metabolite profiling of different stress samples (Cold, flood, drought and salt) was performed as described by Akhtar et al. [38] using Gas Chromatography (MSD 7890A, Agilent Technologies) equipped with autosampler, HP-5MS column and 7977A mass detector. For identification and quantification of metabolites, 100mg of fresh leaves were ground in ice cold MTBE (tert-butyl methyl ether) (Sisco Research Laboratory, India) along with toluene (Sisco Research Laboratory, India) as internal standard. The extracts were dehydrated by adding anhydrous sodium sulfate and 1μl of the each extracts were injected to GC-MS system. All the samples were run in triplicates and the average with standard deviation were considered for data interpretation.

### Quantitative and semi-quantitative RT–PCR analysis

SYBR Green chemistry (Applied Biosystems) was applied for performing the quantitative RT–PCR as described by Rastogi et al. [21]. Primer designing for the validation of DGE and to study the effect of four abiotic stresses on the phenylpropanoid pathway genes through qRT-PCR was carried out with the help of software Primer Express version 2.0 (Applied Biosystems) and were procured from Eurofins Genomics India Pvt. Ltd. (S1 Table). Triplicates of each reaction were run on Fast Real Time PCR System model ‘7900HT’ (Applied Biosystems) [21, 22].

## Results and discussion

### *De novo* transcriptome sequencing and assembly

The RNAseq PE sequencing libraries were made using the RNA samples of all the four stresses (cold, drought, flood, and salt) and a control leaf sample. PE illumina libraries were sequenced on NextSeq 500 using 2 x 75 bp chemistry. The mean of the libraries fragment size distributions were 434 bp, 399 bp, 419 bp, 467 bp, and 381 bp for control, cold, drought, flood, and salt samples, respectively. The sequenced raw data was processed using Trimmomatic v0.35 in order to obtain clean high quality reads. Statistical summary of RNA sequencing and assembly has been provided in S2 Table. *De novo* assembly was then carried out using these paired end reads. The filtered high quality reads were assembled into transcripts using Trinity RNA seq assembler. A total of 40,560 (control), 32,731 (cold), 36,502 (drought), 34,650 (flood) and 27,811 (salt) coding sequences (CDS) were identified from within 64,603 (control), 55,891 (cold), 58,018 (drought), 55,956 (flood) and 46,235 (salt) unigene transcripts (S3 Table) using TransDecoder. Summary of unigene and CDS statistics is presented in S3 Table. Deviation in the number of assembled unigene transcript of control and stress (cold, drought, flood and salt) samples may possibly be because of the inconsistent response of the plants against the stresses, or might have resulted due to some technical noise arisen at some step of sequencing procedure [39]. The assembled unigene transcripts ranged between 201 to >13,338 bases in length. Distribution number of coding sequences (CDS) and unigene according to their length has been provided in the S2 Fig, where the highest transcripts among the assembled transcript data of all the samples were in the size range of below 500 bp, subsequently transcripts ranging between 1,001–1,500 bp. The comparable range of transcript length and abundance has also been prior reported from *O*. *tenuiflorum* in a comparison of its transcriptome data with that of *O*. *basilicum* [22].

### Differential gene expression

**Fig 2:**
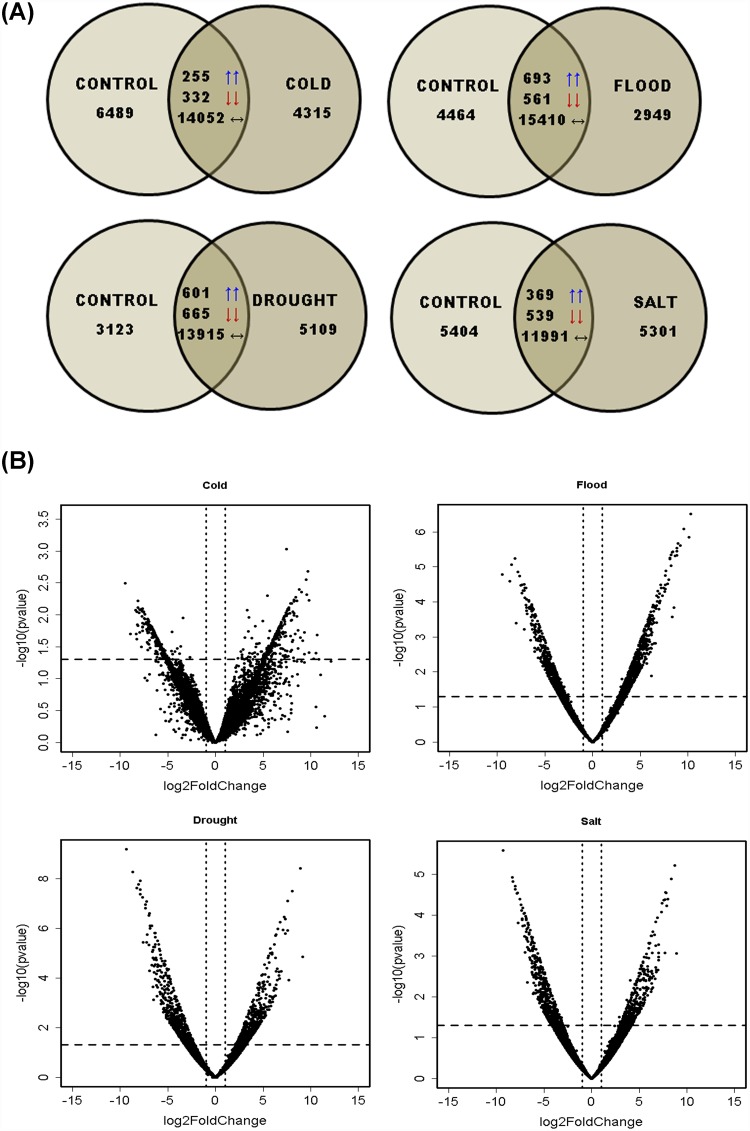
Differentially expressed genes distribution. **(A)** Venn diagrams representing commonly up-regulated, down-regulated, neutral and exclusive transcripts in each stress condition with respect to control. **(B)** Volcano plot represents significantly the up- and down-regulated transcripts having fold changes >1 and <-1, respectively where each point signifies a DEG. Points lying above the horizontal line and left to the vertical line were significantly down- regulated (p < 0.05) while the ones below the horizontal line on the same side were non- significantly down-regulated. On the other hand, points above the horizontal line and right to the vertical line were found to be significantly up-regulated while the ones below the horizontal line on the respective side were non-significantly up-regulated.

**Fig 3:**
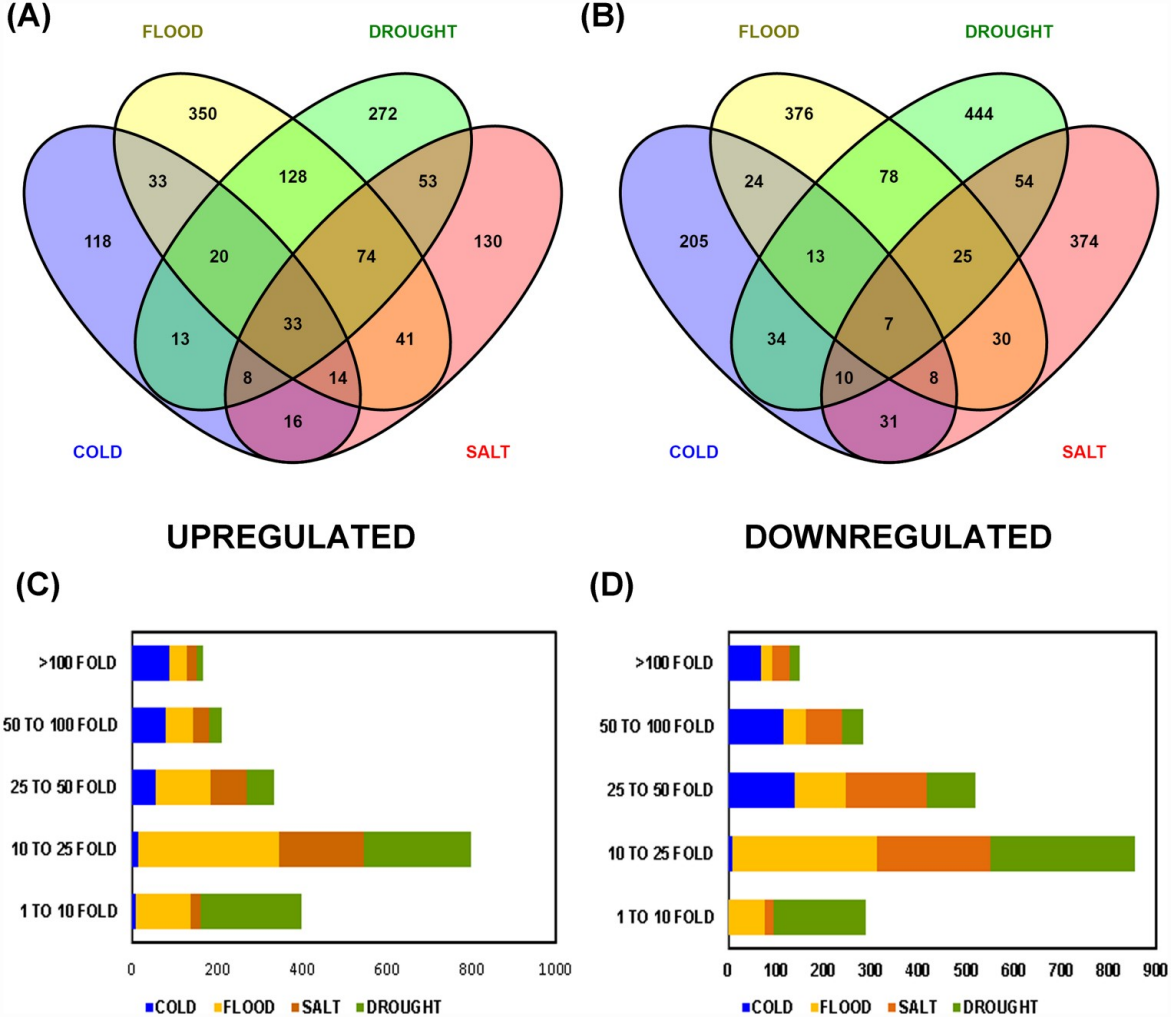
Differentially up-regulated and down-regulated transcripts in all the 4 stresses. (A) Venn diagram illustrating the common and exclusive transcripts that are differentially up- regulated. **(B)** Venn diagram illustrating the common and exclusive transcripts that are differentially down-regulated. **(C)** Bar graph representing the differentially up-regulated transcripts classified on their fold change expression basis. **(D)** Bar graph representing differentially down-regulated transcripts classified on their fold change expression basis.

**Fig 4:**
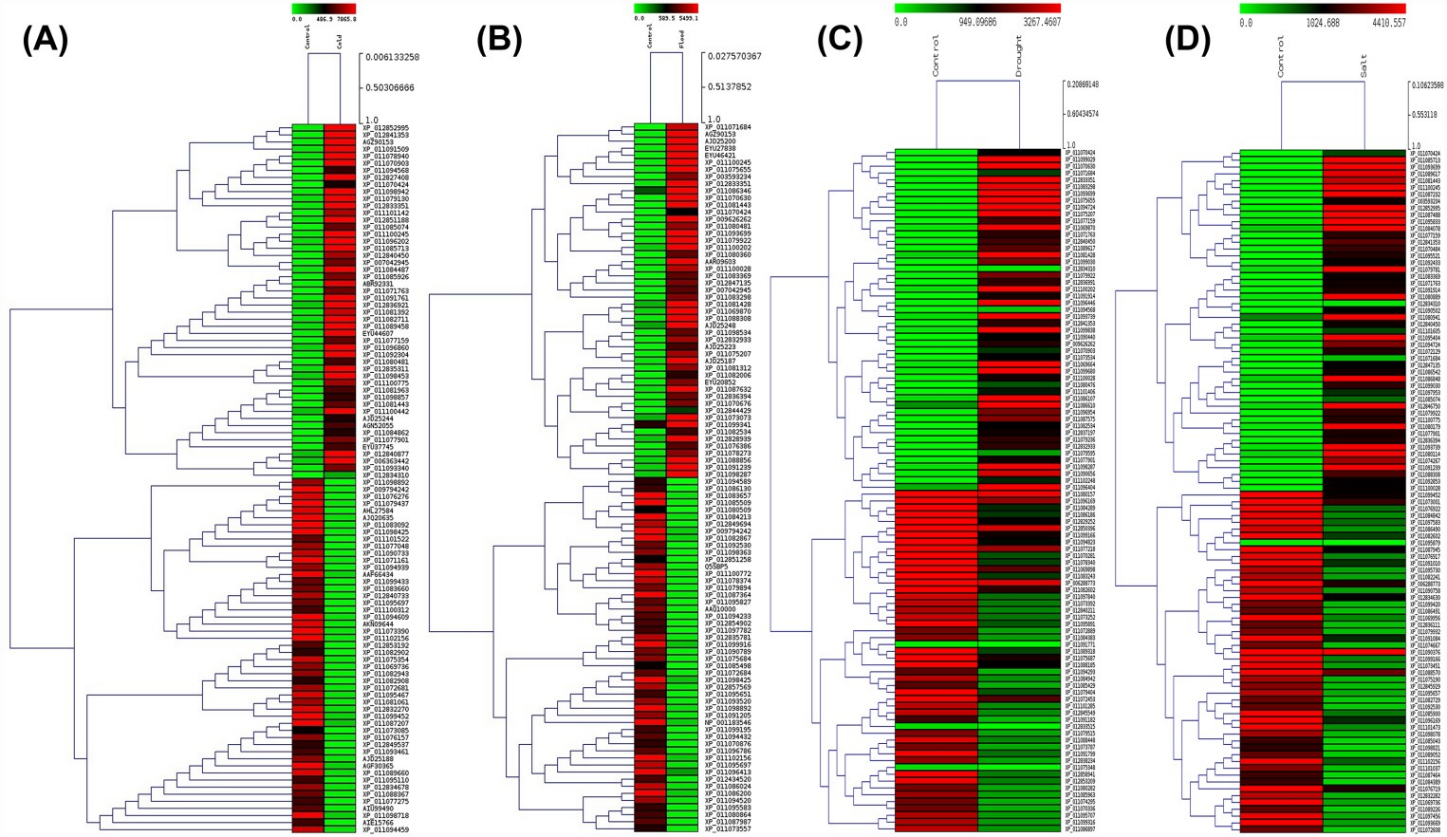
Heatmap of differentially expressed genes. **(A)** Control vs. Cold **(B)** Control vs. Flood **(C)** Control vs. Drought **(D)** Control vs. Salt.

Differential gene expression analysis was carried out based on the identification of the common hit accessions based on BLAST against NR database performed using DESeq. A total of 14,639 (cold), 16,664 (flood), 15,181 (drought) and 12,899 (salt) transcripts were common in each stress with respect to the control. Further, these genes were categorized as up-regulated or down-regulated based on their fold change values. Genes with fold change value more than zero were considered to be up-regulated whereas less than zero as down-regulated. Statistically significant results were filtered using the threshold p-value and p-adjusted value ≤0.05. In flood, drought and salt treatments significant Benjamini-Hochberg adjusted P values ≤0.05 [40] were obtained, while it was not so in case of cold stress treatment, hence, the transcripts with p-value ≤0.05 were considered for analyzing the differentially expressed transcripts under cold stress. Venn diagram in the Fig 2A summarizes the number of genes which are up-regulated, down-regulated, having no change and also the genes exclusive to each of the four abiotic stresses (cold, flood, drought, salt). From the venn diagram, it could be inferred that maximum number of commonly expressed transcripts showed no change in all the four abiotic stresses with respect to control. However, transcripts showing down-regulation were higher than the up-regulated ones in all the stresses except the flooding stress and the transcripts exclusive to stress were lower than control except in drought stress where the exclusive transcripts were more in number as compared to control. Whereas the volcano plots in the Fig 2B represent significantly the up- and down-regulated transcripts having fold changes >1 and <1, respectively. Transcripts above the horizontal line and left to the vertical line were significantly down-regulated while the ones below the horizontal line on the same side were non-significantly down-regulated. On the other hand, transcripts above the horizontal line and right to the vertical line were found to be significantly up-regulated while the ones below the horizontal line on the respective side were non-significantly up-regulated. However, the transcripts that lie between the two vertical lines were neutral in expression. A total of 255 (cold), 693 (flood), 601 (drought), and 369 (salt) genes were found to be up-regulated in the differential gene expression while 332 (cold), 561 (flood), 665 (drought), and 539 (salt) genes were down-regulated. Fig 3A and 3B represents separate Venn diagram of the up- and down-regulated genes showing the common as well as the exclusive genes expressed in the course of stress condition. It was observed that 33 transcripts were commonly up-regulated while only 7 transcripts were commonly down-regulated in all the four stresses, the annotations of these transcripts are provided in S4 Table and sequences provided in S5 Table. In all the four abiotic stresses of the present investigation, out of the 33 commonly up-regulated and 7 commonly down-regulated transcripts, 20 and 3 transcripts, respectively (S4 Table), were the ones which were previously reported to be involved in stress response [41–48]. Primary role of PP2C genes is in stress tolerance, particularly ABA response, hence up-regulation of this transcript (CDS_21223_Unigene_34825) in all the long term stresses may easily be attributed [49]. F-box proteins (CDS_721_Unigene_1749) in plants are reported to be induced by abiotic and biotic stresses [41]. Na+/H+ (AtNHX1) and K+/H+ (AtNHX3) antiporters from *Arabidopsis* have been demonstrated for improved tolerance (CDS_39398_Unigene_61545) against salt and drought stresses in transgenic poplar [42]. Cytosolic sulfotransferase (OsSOT9) gene from rice has been reported as a novel candidate (CDS_38924_Unigene_60033) concerned to stress tolerance [43]. Wang et al. [44] have demonstrated that RPN1a, the *Arabidopsis* 26S proteasome subunit (CDS_12189_Unigene_22188) is necessary for optimal growth of plant as well as stress responses. Though, cyclic nucleotide-gated ion channel (CNGC) family members (CDS_14008_Unigene_24746) are recognized for their role in the cations uptake of Na+, K+ and Ca2+ ions thereby, regulating growth and development of plant, Jha et al. [45], have also demonstrated its role in imparting abiotic and biotic stress tolerance. Commonly down-regulated transcripts among all the four abiotic stresses in the present investigation are- coiled coil domain-containing 150 (CDS_26797_Unigene_42479), flavanone 3-dioxygenase-like (CDS_20547_Unigene_33835), methylthioribose kinase (CDS_1507_Unigene_3270), and GDSL esterase lipase At4g18970-like (CDS_24654_Unigene_39576). Plant long coiled-coil protein, F3H (2-oxoglutartate-dependent dioxygenase) and GDSL lipase have been reported to be involved in a plant defense response [46–49] and since these transcripts were found to be down-regulated in all the stresses of the present investigation, making the defense system of plant weak, to resist the abiotic stresses imposed on the plant. A highest range of 10–25 fold change expression was observed in both up- and down-regulated genes followed by 25–50 fold (Fig 3C and 3D). A complete linkage hierarchical cluster analysis was also carried out on top 100 differentially expressed genes using MEV v4.8.1, representing top 50 up-regulated and top 50 down-regulated transcripts in each stress. Level of expression in the heat map generated represents log2 ratio of gene abundance between control vs. each of stress samples (cold, flood, drought, salt) discretely (Fig 4, S6 Table).

### Gene ontology and KEGG pathway analysis

Candidate coding regions or the CDS predicted (S7 Table) from the unigenes were searched against the non-redundant protein database of NCBI using Blast x. More than 94% of the CDS in each sample got annotated while less than 4% showed no significant Blast hit (S7 Table). Blast2GO program was used for gene ontology (GO) and assigning roles to the predicted CDS (S8 Table). Predicted CDS of control, cold, drought, flood, and salt were mapped onto the standard pathways available in the KEGG database [50]. CDS of all the samples were categorized under five categories: Metabolism, Cellular processes, Genetic information and processing, Environmental information processing and Organismal Systems. A total of 11,107 (control), 9,678 (cold), 10,201 (flood), 8,073 (drought) and 6,890 (salt) CDS got functionally assigned in KEGG out of which only 441 (control), 379 (cold), 413 (flood), 298 (drought) and 260 (salt) CDS were attributed to the metabolism and biosynthesis of secondary metabolites (S3 Fig, S6 Dataset). CDS exhibiting significant up and down-regulation with p value ≤0.05 in all four stresses were also mapped in KEGG database against the terms.

### Differentially expressed genes (DEGs)—Enrichment analysis

**Fig 5:**
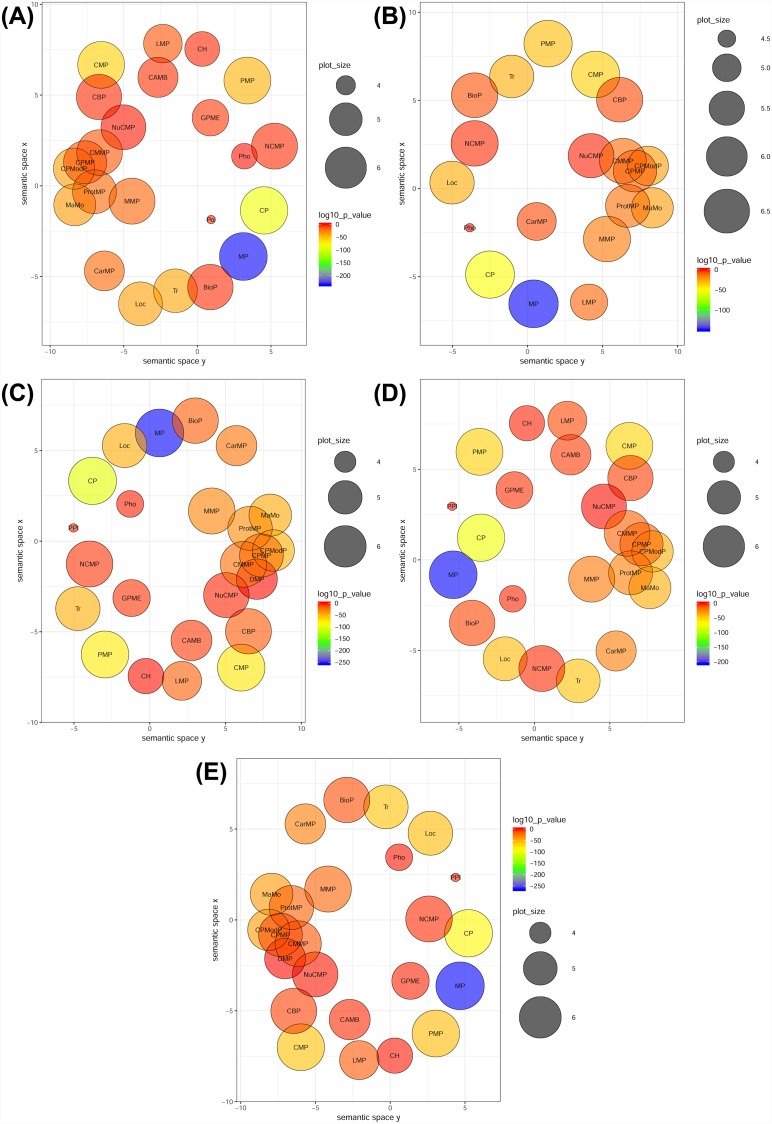
GO enrichment analyses of biological process (BP) terms using REVIGO. (A) Significantly enriched transcripts in BP GO terms related to cold treatment. **(B)** Significantly enriched transcripts in BP GO terms related to flood treatment. **(C)** Significantly enriched transcripts in BP GO terms related to drought treatment. **(D)** Significantly enriched transcripts in BP GO terms related to salt treatment. **(E)** Significantly enriched transcripts in BP GO terms related to control. Circles indicate the GO terms (Plant GO slims) clustered on the basis of semantic identities to other GO terms in ontology (larger circles depict more general terms, while adjacent circles are strongly related). Size of the circle is relative to the GO term frequency where the colors indicate the enrichment in form of log10P-value from the AgriGO results (blue lower, red higher). **Abbreviations**: lipid metabolic process, LMP, transport, Tr, establishment of localization, EL, metabolic process, MP, pollination, Pol, cellular process, CP, localization, Loc, generation of precursor metabolites and energy, GPME, photosynthesis, Pho, cellular metabolic process, CMP, biosynthetic process, BioP, nitrogen compound metabolic process, NCMP, cellular protein modification process, CPModP, carbohydrate metabolic process, CarMP, cellular homeostasis, CH, homeostatic process, HP, primary metabolic process, PMP, macromolecule metabolic process, MMP, cellular biosynthetic process, CBP, macromolecule modification, MaMo, cellular amino acid metabolic process, CAMB, nucleobase-containing compound metabolic process, NuCMP, protein metabolic process, ProtMP, cellular macromolecule metabolic process, CMMP, cellular protein metabolic process, CPMP, pollen-pistil interaction, PPI, DNA metabolic process, DMP.

Each of the four stress samples were explored and analyzed to recognize that which of the GO terms and KEGG pathways got enriched. The FDR was also calculated in order to identify the percentage of false predictions in the prediction dataset. In cold, drought and salt stresses 46 GO terms while in flood stress 33 GO terms for the DEGs were found significantly enriched (FDR ≤0.05). Under the biological process category, “cellular process”, “metabolic process” and the “localization” GO terms were the most affected and therefore enriched significantly in all the four abiotic stresses (Fig 5). These processes are imperative to the growth of plant and its survival [39]; however, they may also be indicative of the plant response against four different stresses given to the plant in this investigation. In the molecular function category, a noticeable effect was observed in the GO terms exhibiting “catalytic activity”, “binding”, “transporter activity”, and “structural molecule activity”(S4 Fig) representing the dominion of signal transduction and the genetic regulation of the active enzymatic processes [39]. In addition to the GO terms enrichment, the KEGG pathway enrichment in the up-regulated and the down-regulated DEGs was also carried out using KOBAS 3.0 web server (S9 Table, S7 Dataset). In KOBAS analysis, only 10 and 4 transcripts up-regulated in flooding and salt treatments, respectively, were found to be highly significant i.e. with corrected p-value (p adjusted) of ≤0.05. The important flood up-regulated DEGs were the GO terms- “Biosynthesis of secondary metabolites” (corrected p-value = 0.0002), “Flavonoid biosynthesis” (corrected p-value = 0.0295), “Limonene and pinene degradation” (corrected p-value = 0.0544), and “Stilbenoid, diarylheptanoid and gingerol biosynthesis” (corrected p-value = 0.0205). Since, secondary metabolites in plants play an important role in stress responses [51], therefore these terms related to secondary metabolites might have got up-regulated. Highly significant up-regulated DEGs GO terms in salinity stress i.e. with corrected p-value ≤0.05, were “Carbon metabolism”, “Spliceosome”, “Carbon fixation in photosynthetic organisms”, “Pentose phosphate pathway” etc. In addition to the changes in carbon and starch/ sugar metabolism, ribosomes also play an important role. In response to the environmental stresses the plants organize the interaction between eukaryotic and prokaryotic ribosomes to maintain the balance of protein synthesis in cell for the cell survival under the stress environment [52]. Hence, the main aim of the enrichment analysis was to analyze the family of proteins or genes that are excessively represented in a considerably large set of proteins or genes, and may possibly be associated with stress response and defense.

### Validation of DGE data

**Fig 6:**
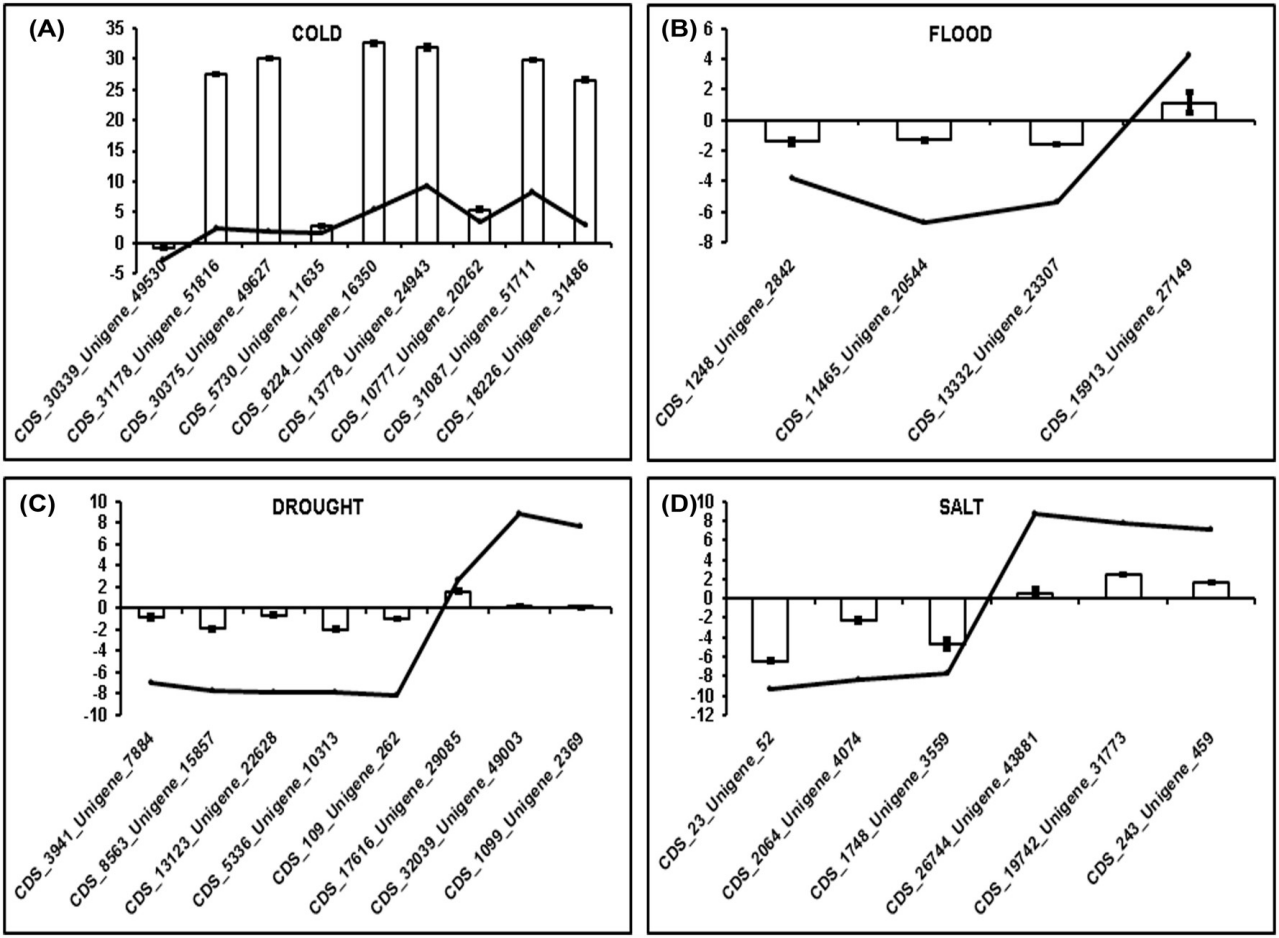
Validation of some of the up-regulated and down-regulated DEGs using qRT-PCR. Columns in the graph represent the expression pattern (log2 fold change values) of selected transcripts using total RNA isolated from leaf tissues through quantitative Real time PCR while line represents the digital gene expression values of the same transcripts.

A qRT-PCR study to validate the digital gene expression (DGE) data has also been conducted for some of the up-regulated and down-regulated DEGs as represented in the Fig 6. Transcripts for validation included some of the stress responsive transcripts and some were randomly selected.

#### Cold stress

Though in cold stress the significant p-values of ≤0.05 were only obtained and not significant p- adjusted of ≤0.05, however, upon validation of DGE data with qRT-PCR it was found that gene expression pattern was similar to that of the DGE data. Hence, p-value of ≤0.05 was considered in this analysis, where p-adjusted or corrected p-value was not ≤0.05. As shown in Fig 6A for cold stress, out of the 8 transcripts, 4 transcripts were related to the cold stress response (CDS_30339_Unigene_49530, CDS_5730_Unigene_11635, CDS_8224_Unigene_16350, and CDS_10777_Unigene_20262). “CDS_30339_Unigene_49530” transcript was annotated as “ESKIMO 1-like”. ESKIMO1 as a key gene involved in conserving plant water as well as cold acclimation and salt tolerance Bouchabke-Coussa et al. [53]. Since, ESKIMO1 gene functions in cold tolerance and therefore plant will not be able to combat cold stress if the gene is down-regulated. In the DGE data this transcript was found to be down-regulated which was also evident in the qRT-PCR result. Another transcript was “CDS_5730_Unigene_11635” annotated as “pentatricopeptide repeat-containing At1g56570 isoform X2”. As reported in case of rice, pentatricopeptide repeat protein (PPR) is essential for chloroplast biogenesis under cold stress [54]. Under the cold stress, there were no symptoms of leaf decoloration observed and also the transcript expression in qRT-PCR shows up-regulation, thus the DGE data exhibiting the up-regulation of the same transcript also gets established. In the series, “CDS_8224_Unigene_16350” which is annotated to be “probable calcium-binding CML44” is a cold responsive calmodulin-like gene (CML). This transcript was found to be up-regulated in both qRT-PCR data as well as the DGE data of the 72 hour cold stress treated plant leaves which might be responsible for withstanding the 72 hours of stress in extremely cold sensitive *Ocimum* plant. Over-expression of *ShCML44* gene from *Solanum habrochaites* is attributed to the greater tolerance to cold, drought, and salinity stresses [55]. “CDS_10777_Unigene_20262” was another cold stress response related transcript annotated to be “homeobox-leucine zipper ATHB-12-like (HD-Zip)” which was found to be up-regulated in both the qRT-PCR results as well as the DGE data. As reported by Zhang et al. [56] low expression level of HD-Zip genes provided the defensive and tolerant protection to the tomato plant against cold stress. Therefore higher expression of this transcript is indicative of the susceptibility of experimental plant *O*. *tenuiflorum* against the cold stress.

#### Flooding stress

A total of 4 transcripts were selected from the flooding stress data for the DGE validation with qRT-PCR (Fig 6B). All the 4 transcripts (CDS_1248_Unigene_2842, CDS_11465_Unigene_20544, CDS_13332_Unigene_ 23307, and CDS_15913_Unigene_27149) were related to the flooding stress response. “CDS_1248_Unigene_2842” annotated as “IQ-DOMAIN 14” was found to be down-regulated in the qRT-PCR which was in synchronization with the DGE data. As also reported by Komatsu et al. [57], protein IQ-DOMAIN 31-like was among those set of proteins which decreased under the flooding stress. The next transcript “CDS_11465_Unigene_20544” was described to be “DNA-damage-repair toleration DRT100-like” which was also identified as gene expression biomarker for tolerating waterlogging in *Prunus* [58]. This transcript was also found to be down-regulated both in the qRT-PCR as well as the DGE data which might be the reason that after 30 days of stress the plant was unable to tolerate the water-logging stress any further. Another transcript “CDS_13332_Unigene_23307” identified as “BTB POZ domain- containing At3g22104-like” showed down-regulation in qRT-PCR as well as the DGE data. As per the reports of Yin and Komatsu [59], there was down-regulation of BTB domain containing protein 47 in the soybean root tips under flooding stress. The last transcript taken for validation of the flooding stress was “CDS_15913_Unigene_27149” designated as “zinc finger ZAT10- like”. This transcript showed up-regulation in both the DGE as well as qRT PCR data. ZAT 10 was reported to be involved in regulating the responses to stresses mediated by ROS, suggesting their potential roles in protecting photosynthesis from the injury during water stress [60].

#### Drought stress

A total of 8 transcripts were selected for validation of drought sample DGE data and all of them were ascribed to be involved in stress response (Fig 6C). “CDS_3941_Unigene_7884 (a kinesin- like calmodulin-binding homolog)” and “CDS_5336_Unigene_10313 (kinesin KCA2)” are first and fourth transcripts exhibiting down-regulation in both the DGE and qRT-PCR results, belong to CML family. CML family members are found to be highly induced by abiotic stress factors like drought, salt and osmotic stress, etc. but often the pattern of expression in individual members of the CaM/CML family vary temporally, spatially and in the degree of response against stimuli, signifying specific functional roles in signal transduction [61]. However, experimental confirmation of the utility of CMLs as stress tolerance regulators is lacking [62]. The second transcript is a “transcription-associated 1-like isoform X4” (CDS_8563_Unigene_15857) which also shows down-regulation in DGE as well as qRT-PCR data. Transcription factors correspond to the crucial molecular switches coordinating the regulation of plant developmental processes in response to diverse stresses [63]. The third transcript is “glycine dehydrogenase (decarboxylating) mitochondrial” (CDS_13123_Unigene_22628). Drought-sensitive cultivar of *Cynodon dactylon* exhibited decreased expression of glycine dehydrogenase [64] which could be easily attributed to the down-regulation of this transcript in the DGE as well as the qRT-PCR data of the drought sample. Fifth transcript is annotated as “callose synthase 3-like” (CDS_109_Unigene_262). Callose is a polysaccharide which exists in the cell walls of a wide variety of higher plants. Down-regulation of this transcript in DGE and qRT-PCR data is indicative of the decreased mechanical strength required by the plant to resist and recover from drought [64]. The sixth transcript “EARLY RESPONSIVE TO DEHYDRATION 15 or ERD15 (CDS_17616_Unigene_29085)” was found to be up-regulated in DGE and qRT-PCR results. Shao et al. [65] have reported that ERD15 gene from the roots of sweet potato is involved in defense response to drought which could be related to the fact that *Ocimum* plant was able to withstand drought until a period of 30 days. “Receptor kinase FERONIA” (CDS_32039_Unigene_49003) is the next transcript taken up for validating the up-regulation in the DGE and qRT-PCR data. It has also been reported in the literature that FERONIA (FER) pathway inhibits ABA signaling and also a significant increase in the ABA content in plant cells significantly have been documented under drought, hence FER provides tolerance against drought in long term stress treatment provided in the current study [66]. The last transcript for drought stress DGE and qRT PCR data validation was “polyphenol oxidase chloroplastic-like” (CDS_1099_Unigene_2369) which was found to be up-regulated. Up-regulation of this transcript was attributed to the drought tolerance in long duration stress, as Chakhchar et al. [67] also reported that moderate and severe drought stress increased significantly the catalase, superoxide dismutase, peroxidase, polyphenoloxidase and lipoxygenase activities, with respect to time.

#### Salinity stress

In case of validation of salinity stress DGE data by qRT PCR (Fig 6D), a total of six transcripts were selected, three of which showed down-regulation (CDS_23_Unigene_52; CDS_2064_Unigene_4074; CDS_17489_Unigene_28412) and three up-regulation (CDS_26744_Unigene_43881; CDS_19742_Unigene_31773; CDS_243_Unigene_459). Out of these transcripts CDS_23_Unigene_52 (acetylajmalan esterase-like); CDS_2064_Unigene_4074 (zinc-finger homeodomain 9-like) and CDS_243_Unigene_459 (vacuolar amino acid transporter 1) were random selections with high differences in the base mean values of control and stress samples, while CDS_17489_Unigene_28412 (UDP-glucuronic acid decarboxylase 2-like); CDS_26744_Unigene_43881 (cyclic nucleotide-gated ion channel 2-like); CDS_19742_Unigene_31773 (NRT1 PTR FAMILY–like) were salt stress responsive transcripts. Bai et al. [68] reported that UDP-glucuronic acid decarboxylase was one of the differentially expressed proteins showing the reduced expression while performing the proteomic analysis of salt responsive proteins in oat roots and transcript (CDS_17489_Unigene_28412) from this study also shows down-regulation in both DGE and qRT PCR data. Jha et al. [45] have demonstrated by their salinity stress experiments in *Arabidopsis* that cyclic nucleotide-gated ion channels (CNGCs) like CNGC1, CNGC19 and CNGC20 increase under salinity in roots, while transcript levels of CNGC19, CNGC3 and CNGC8 increased in shoots. DGE and qRT PCR data of salinity stress experiments in this investigation also show an up-regulation of “cyclic nucleotide-gated ion channel 2-like” transcript (CDS_26744_Unigene_43881) in the leaves. The last transcript “NRT1 PTR FAMILY (NPF)–like” (CDS_19742_Unigene_31773) shows up-regulation in the DGE as well as the qRT PCR results which is also reported by Taochy et al. [69] that NPF2.3 transporter from *Arabidopsis* expresses constitutively for NO(3)(-) translocation to the shoots under salinity stress.

### Transcripts having antagonistic regulation

The transcripts that are up-regulated or down-regulated have been clearly provided in the DGE data, however, upon analysis it was observed that some transcripts were found to be up-regulated in one abiotic stress while down-regulated in another. Such types of transcripts with an antagonistic behavior have been discussed here. CDS_7254_Unigene_14466 transcript which is annotated as “xylosyltransferase 1” was found to be down-regulated in cold stress and up- regulated in flood and salinity stresses. Baldwin et al. [70] have also reported that the xylosyltransferases are ascribed to the improved tolerance against cold in the pea frost genotype that might have resulted due to the increased production of the galactan and arabinan side chains acting as the gelling elements providing protection against frost. Hence, death of *O*. *tenuiflorum* plant in 72 hours under cold stress could easily be related to the down-regulation of this xylosyltransferase transcript (CDS_7254_Unigene_14466). On the other side, Nanjo et al. [71] mentioned that xylotransferases constitute the flooding stress indicator proteins which help to trigger plant defense against the flooding stress. Therefore, up-regulation of xylosyltransferase transcript (CDS_7254_Unigene_14466) under flooding stress could easily be associated to the survival of plant under flooding stress for upto a month. Transcript CDS_34490_Unigene_53496 showing similarity to the “probable leucine-rich repeat receptor kinase At1g68400” was down-regulated in cold and up-regulated in drought stresses. As reported by Liao et al. [72], a leucine rich repeat receptor-like kinase (OsLRR2) gene isolated from rice was able to sustain cold and drought treatments when up-regulated. Possibly this could be the reason that the down-regulation of this LRR transcript (CDS_34490_Unigene_53496) leads to death of the plant under cold stress while its up-regulation promoted tolerance against drought stress. One common transcript (CDS_38353_Unigene_58711) which got up-regulated in flooding stress and down-regulated in drought stress was predicted to be an “uncharacterized protein LOC105169226” and another transcript (CDS_31348_Unigene_48902) was also an “uncharacterized protein LOC105172555” which was up-regulated in flooding stress and down-regulated in salt stress. The role of such transcripts needs to be worked upon in order to define its definite function in the abiotic stress tolerance among the plants. Interestingly on the other side, there were six transcripts that were observed to be down-regulated in flooding stress while the same were up-regulated in drought stress. Out of these six transcripts, CDS_969_Unigene_2245 was annotated to be “phosphomethylpyrimidine chloroplastic isoform X2” which helps in synthesis of 4amino-2-methyl-5-phosphomethylpyrimidine taking aminoimidazole ribotide as a substrate [73] but the exact role of this transcript in abiotic stress cannot be defined without further confirmations and validations. Second transcript CDS_10439_Unigene_19668 was similar to “BTB POZ and TAZ domain-containing 1 protein” which has been accredited to resistance related genes against *Botrytis cinerea* in *Arabidopsis thaliana* by Hao et al. [74]. Third transcript CDS_15938_Unigene_27513 got annotated as “GRIP and coiled-coil domain- containing 2-like protein” referred as golgins due to the localization in the outer membrane of the trans-Golgi network [75]; however, its role in the biotic or abiotic stress has not been documented. Fourth transcript CDS_29256_Unigene_45959 showing identity with “alpha-1,4 glucan phosphorylase L-2 chloroplastic amyloplastic-like protein” was described for its ability to persist transient water shortage and not for starch degradation which is one of the characteristics of glucan phosphorylases [76], hence, its up-regulation in drought stress could easily be correlated with the drought tolerance of *O*. *tenuiflorum* plant in the present study. Fifth transcript CDS_13712_Unigene_24313 exhibited similarity to the “two-component response regulator-like APRR9 protein” and Li et al. [77] have described its role in cold endurance but there are no reports of its involvement in the flooding or drought stresses. The sixth transcript CDS_20119_Unigene_33253 again was predicted to be the “uncharacterized protein LOC105177106” that showed down-regulation in flood stress while up-regulation in drought stress. One transcript (CDS_14745_Unigene_25836) that got down-regulated in drought and up-regulated in salt stress showed similarity to the “pathogen-related–like protein” and such proteins have multiple roles in the biotic as well as abiotic stress tolerance [78]. On the contrary one transcript (CDS_11261_Unigene_20868 annotated as “21kDa” showed up-regulation in drought stress while down-regulation in salt stress, but no definite role of this 21kDa protein has been documented in the literature for its involvement in plant stress.

### Transcription factors

**Fig 7:**
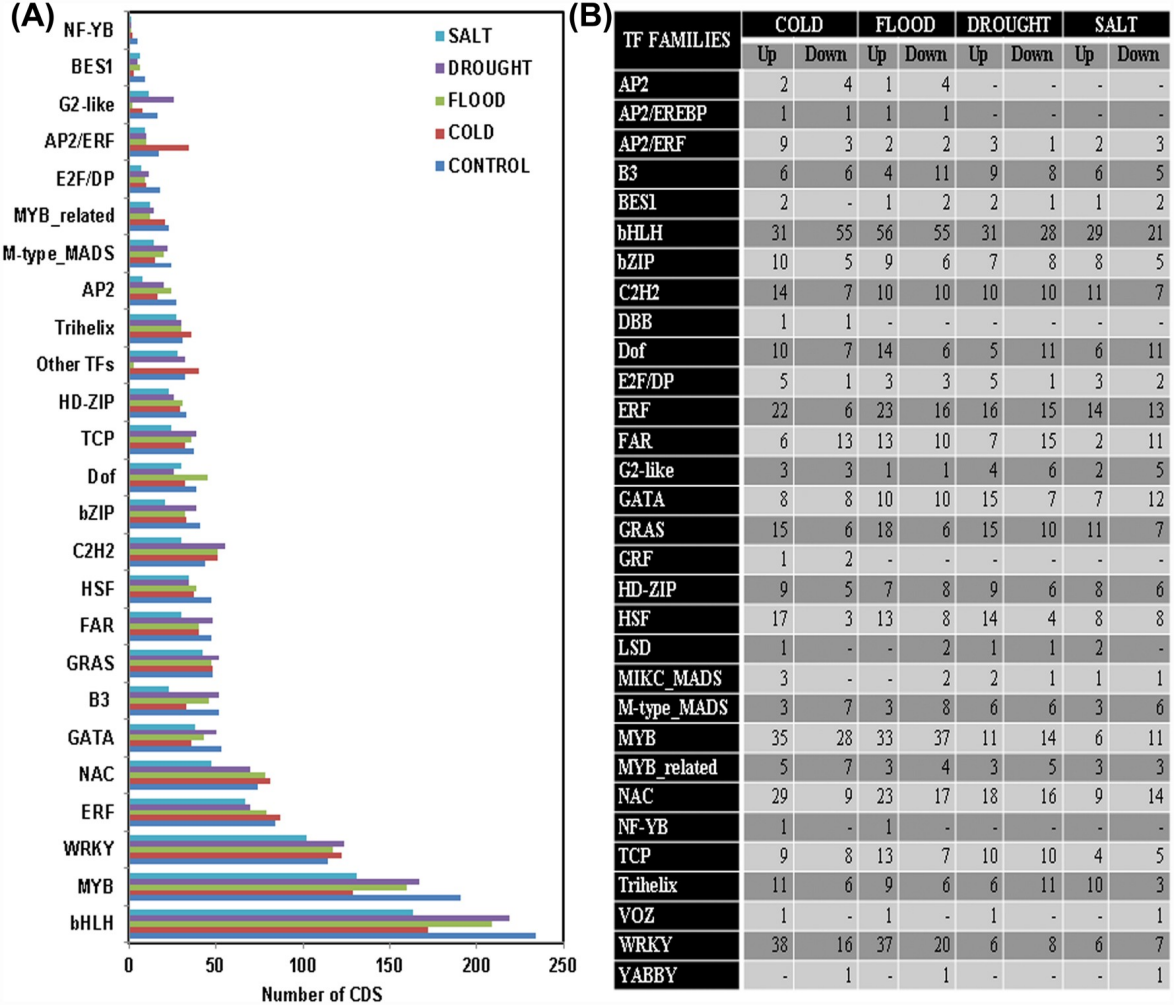
Transcription factor families’ distribution among four abiotic stresses. (A) Bar diagram representing the number of transcripts related with TF families. (B) The number of transcripts representing up-regulated and down-regulated TF families in each abiotic stress.

Numerous genes get triggered as a consequence of abiotic stresses at the level of transcription, and as a result gene products help in providing stress tolerance by producing the crucial metabolic proteins, thereby regulating the downstream genes [79]. These triggers or the regulatory genes could be identified and utilized further for the generation of stress tolerant plants. The emerging trend in stress biology research has now been the engineering of these regulatory genes rather than manipulating a functional gene. Various transcription factors (TFs) that belong to *MYB*, *AP2/EREBP*, *WRKY*, *bZIP*, *NAC*, *etc*. families are observed to be involved in different abiotic stresses which have been manipulated to develop stress tolerance in plants [80]. In the present investigation, a total of 31 TF families got annotated from among the four abiotic stresses. These were A*P2*, *AP2/EREBP*, *AP2/ERF*, *B3*, *BES1*, *bHLH*, *bZIP*, *C2H2*, *DBB*, *Dof*, *E2F/DP*, *ERF*, *FAR*, *G2-like*, *GATA*, *GRAS*, *GRF*, *HD-ZIP*, *HSF*, *LSD*, *MIKC_MADS*, *M-type_MADS*, *MYB*, *MYB_related*, *NAC*, *NF-YB*, *TCP*, *Trihelix*, *VOZ*, *WRKY*, and *YABBY* (Fig 7A). In the DEG analysis it was found that *WRKY*, *MYB*, *bHLH*, *NAC*, and *ERF* transcription factor families were the most affected (Fig 7B). It has also been reported that *MYB/MYC*, *WRKY* and *NAC* regulons play a crucial role in abiotic stress related gene expression in rice [81]. Reports of the subsistence of an intricate regulatory system between the abiotic stress signals and expression pattern of the corresponding responsive gene was also evident in *Arabidopsis* [82]. Engineering of these genes in order to regulate the downstream stress response genes likely appears to be a potential attempt for developing various stress (drought, salt and cold) tolerant transgenic plants in comparison to the distinct functional genes [83]. Since there are many reports on the engineering of the TFs for developing transgenic stress tolerant plant varieties, information of the TFs generated from the NGS sequencing of 4 abiotic stress samples in *Ocimum* leaves could be a source of some new TFs with direct/ indirect roles in developing of the tolerant plant varieties.

### The effect of different abiotic stresses on the phenylpropanoid biosynthesis in *O*. *tenuiflorum*

**Fig 8:**
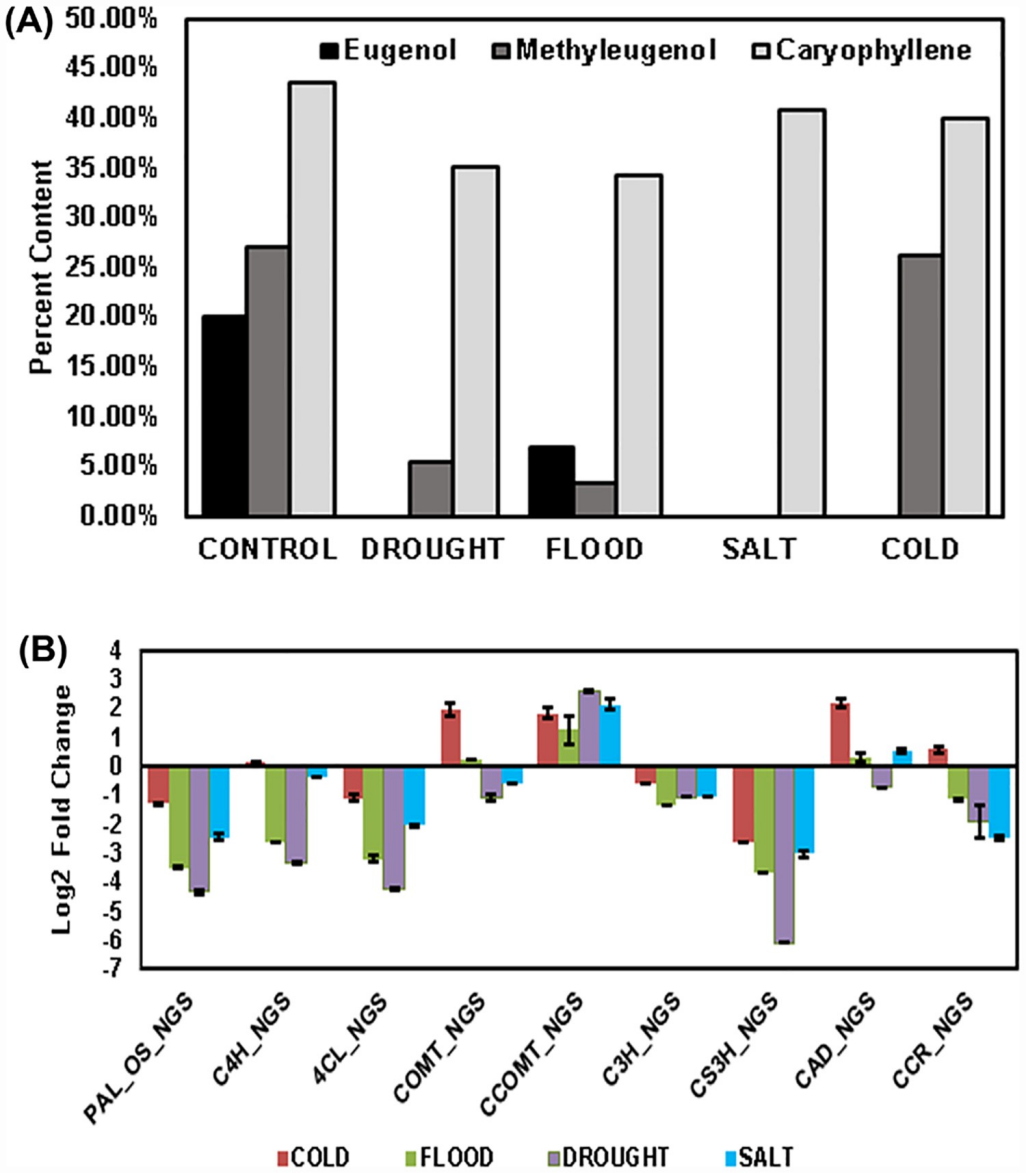
Metabolite profiling and pathway gene expression analysis. (A) Graph showing the effect of the four abiotic stresses on the key metabolites. (B) qRT-PCR analysis of the phenylpropanoid pathway genes in all the stress samples. Analysis was carried out in triplicates, and standard deviation is represented with error bars. **Abbreviations**: *PAL* (Phenylalanine ammonia lyase), *C4H* (Cinnamte 4- hydoxylase), *4CL* (4-Coumarate ligase), *COMT* (Caffeic acid O-methyltransferase), *CCOMT* (Caffeoyl CoA O-methyltransferase), C3H (Coumarate 3- hydroxylase), *CS3’H* (pCoumaroyl shikimate 3′-hydroxylase), *CAD* (Cinnamoyl alcohol dehydrogenase), *CCR* (Cinnamoyl CoA reductase).

Phenylpropanoids are a source of numerous secondary metabolites that play important roles in plant responses against abiotic and biotic stimuli [84]. Polymers of phenylpropanoids (tannins, suberins or lignins) have been reported to impart strength and firmness to the angiosperms and gymnosperms towards environmental or mechanical harm (like- wounding, drought, etc.) [85]. Evidences of the secondary metabolite accumulation in plants under various biotic and abiotic stresses could be reviewed from the literature [86, 13]. Various stresses have a great influence on the phenylpropanoids class of compound tending to increase their accumulation [13]. The incidence of coexisting biotic and abiotic stresses impart an additional level of intricacy, as the reaction to these are mainly organized by various signaling pathways which may either interact or inhibit each other [87, 88]. While investigating the outcome of an abiotic stress with concurrent effect of an herbivore or pathogen, both negative and positive interactions were recorded based on the type, timing, and severity of each abiotic stress. For instance in the drought stress treated common beans as well as sorghum plants were found to be more susceptible to the charcoal rot fungus *Macrophomina phaseolina* [89, 90] and many such examples are reviewed by Atkinson and Urwin [91]. Therefore, when the plants undergo environmental stress they also get exposed to the pathogen attack especially on leaves, on the contrary, the opposite incidence has also been reported [91]. As a result, there is an accumulation of the certain class of phenylpropanoid compounds, like- isoflavonoids, coumarins, furanocoumarins, stilbenes, aurones etc. [92]. However, when there is wounding in the plant, leaves accumulate tannins, lignin, suberin, the wall bound phenolic acids [92] and when there is UV-irradiation, high light, nutrient deficiency or low temperature accumulation of different flavonoids and phenolic acids has been recorded in the leaves [93]. However, there are no reports on any kind of the decrease in the phenylpropanoid compounds which has been observed in the present study. *O*. *tenuiflorum*, is a rich source of eugenol, followed by caryophyllene and methyleugenol. When the plants of *O*. *tenuiflorum* were subjected to the four abiotic stresses (cold, flood, drought and salt), the eugenol and methyleugenol contents were found to be decreased in all the four stress conditions. However, no significant change was observed in the caryophyllene content (Fig 8A). Bettaieb et al. [94], while investigating the effect of drought on polyphenol composition and antioxidant activities in aerial parts of *Salvia officinalis* L. also found that under severe water deficit (SWD) of 25% field capacity there was an increase in the biosynthesis of some phenolic acids, however, a significant decrease in vanillic, *p*-coumaric, cinnamic, dihydroxybenzoic, *trans*-cinnamic, caffeic, ferulic and cinnamic acids was observed. Though, the phenylpropanoids tend to increase in response to biotic/ abiotic stresses [92], however, it has also been reported that the prolonged and severe stresses lead to the reduction in polyphenols [94]. Hence, reduction in eugenol and methyleugenol in the present study could easily be correlated as it has also treated the plants with prolonged as well as severe abiotic stresses. Since, *p-*coumaric (COU), *trans-*cinnamic (CIN), caffeic (CAF) and ferulic acids (FER) are reported to be decreased under SWD [94] which are the main substrates leading to the eugenol and methyleugenol biosynthesis, hence, reduction in both these compounds in the present investigation could easily be related. Similarly, Mandoulakani et al. [95] also observed a decrease in the eugenol content while investigating the effect of drought stress on the expression of key genes involved in the biosynthesis of phenylpropanoids and essential oil components of *O*. *basilicum*. But they observed an increase in the methyleugenol and methylchavicol contents at the drought condition of 50% field capacity which is a moderate stress. In addition to the essential oil profiling and quantification, the gene expression levels of *PAL*, *C4H*, *4CL*, *COMT*, *CCOMT*, *C3H*, *CS3H*, *CAD*, and *CCR* were quantified in leaves (Fig 8B). The results revealed that the stress conditions decreased the expression of *PAL*, *C4H*, *4CL*, *COMT*, *C3H*, *CS3’H* and *CCR* with some exceptions in cold stress. On the other side, *CCOMT* and *CAD* transcripts showed an increased expression except in drought stress where *CAD* also had lower expression in comparison to control. The decrease in eugenol content could be attributed to the fact that the lower expression of the initial phenylpropanoid pathway genes (*PAL*, *C4H*, *4CL*) would lead to the reduced metabolite content as also reported by Rastogi et al. [21] where RNAi of *4CL* gene in agroinfiltrated *O*. *sanctum* (or *tenuiflorum* L.) plants led to eugenol reduction. *CAD* and *CCOMT* transcripts taken in the expression analysis showed an increased expression which might be due to the reason that the selected transcripts are the isoforms which belong to the lignin biosynthetic pathway and as per the reports, there is an induction of lignins under stress conditions, like wounding and low temperatures [92]. There are many reports of studies analysing the effect of various biotic and abiotic stresses on the secondary metabolites of *Ocimum* species [96–102]. But the present investigation is the first report which gives a comprehensive overview of the transcriptomic changes occurring in a very useful medicinal plant, *O*. *tenuiflorum* in the current scenario of the global climatic change.

## Conclusion

In this study, main objective was to get an overall view of the effect of abiotic stresses on the transcriptome and the metabolites of the plant, therefore a comparative transcriptome as well as differential expression analysis of control vs. cold, control vs. flood control vs. drought and control vs. salt abiotic stress treated plants was performed. Among all the four stresses, the plant was most vulnerable to the cold stress as the plant dies after 72 hours of the stress treatment and also the major constituent of the plant i.e. eugenol got decreased to almost negligible. However, the plants were able to sustain upto 30 days of severe flood, drought and salt stresses. The present investigation for the first time, gives an insight of the transcriptomic changes occurring in a very important medicinal plant *O*. *tenuiflorum* under the four different abiotic stresses. The differential gene expression analysis resulted in the identification of differentially up-regulated and down-regulated genes exclusive to each abiotic stress were also recognized which may prove to be significant in exploration of stress tolerance mechanism in *O*. *tenuiflorum* against environmental changes by their functional characterization. Ultimately, present investigation resulted in the identification of several putative abiotic stress tolerant genes which could either be exploited in breeding stress tolerant *O*. *tenuiflorum* through pyramiding or *via* transgenic plant generation. In addition, the putative genes identified in this study could also be used for finding their orthologs in other medicinal and aromatic plants while exploring for stress tolerance in them.

## Supporting information

S1 Dataset Transcript annotation file. (XLSX)

S2 Dataset DGE with annotation Control Vs. Cold. (XLSX)

S3 Dataset DGE with annotation Control Vs. Flood. (XLSX)

S4 Dataset DGE with annotation Control Vs. Drought. (XLSX)

S5 Dataset DGE with annotation Control Vs. Salt. (XLSX)

S6 Dataset KEGG pathway analysis data. (XLSX)

S7 Dataset Enrichment analysis. (XLSX)

S1 Table List of primers used in the study. (DOCX)

S2 Table Statistical summary of RNA sequencing and assembly. (DOCX)

S3 Table Summary of unigene and CDS statistics. (DOCX)

S4 Table Commonly up-regulated and down-regulated transcripts in all the four abiotic stresses (Cold, Flood, Drought and Salt). (DOCX)

S5 Table Sequences of the CDS discussed in the manuscript. (DOCX)

S6 Table Annotations of the transcripts used in Heatmap generation. (XLSX)

S7 Table Summary of coding sequences (CDS) predicted from unigenes and their annotations. (DOCX)

S8 Table GO distribution for CDS. (DOCX)

S9 Table KOBAS analysis. (XLSX)

S1 Fig qRT- PCR for determining the optimal stress conditions for selection of samples for transcriptome sequencing. (PPTX)

S2 Fig Distribution number of coding sequences (CDS) and unigene according to their length. (A) Cold, (B) Flood, (C) Drought, (D) Salt, and (E) Control. Blue bar = Unigene, Red bar = CDS.
(PPTX)

S3 Fig KEGG pathway distribution of CDSs in all the four abiotic stress samples and control. (PPTX)

S4 Fig GO enrichment analyses of Molecular Function (MF) terms using REVIGO. (A) Significantly enriched transcripts in MF GO terms related to cold treatment. (B) Significantly enriched transcripts in MF GO terms related to flood treatment. (C) Significantly enriched transcripts in MF GO terms related to drought treatment. (D) Significantly enriched transcripts in MF GO terms related to salt treatment. (E) Significantly enriched transcripts in MF GO terms related to control. Circles indicate the GO terms (Plant GO slims) clustered on the basis of semantic identities to other GO terms in ontology (larger circles depict more general terms, while adjacent circles are strongly related). Size of the circle is relative to the GO term frequency where the colors indicate the enrichment in form of log10P-value from the AgriGO results (blue lower, red higher).
(PPTX)
