# Supplementary material for: Ocimum metabolomics in response to abiotic stresses: Cold, flood, drought and salinity
Source: PLoS One. 2019 Feb 6;14(2):e0210903. doi: 10.1371/journal.pone.0210903 (PMC6364901; doi:10.1371/journal.pone.0210903)
Supplement: S1 Table — (DOCX) [file pone.0210903.s008.docx]

**S1 Table.** List of Primers used in the study.

| **S.No.** | **PRIMER NAME** | **5'-3' PRIMER SEQUENCE** | **Primer Source** |
| --- | --- | --- | --- |
| 1 | Jasmon_ZIM_SyRT_F | GCATCCAGAGGCGGTATGTT | *O. sanctum* transcriptome data (Rastogi et al., 2014) |
| 2 | Jasmon_ZIM_SyRT_R | CAAGAATCTGGCTAGGGTTGCT | *O. sanctum* transcriptome data (Rastogi et al., 2014) |
| 3 | K.channel_SyRT_R | CGGAAGGAGAGATGATGAAAGG | *O. sanctum* transcriptome data (Rastogi et al., 2014) |
| 4 | LEA_SyRT_F | CCTCATAGTGGATGTGCCTGTGT | *O. sanctum* transcriptome data (Rastogi et al., 2014) |
| 5 | LEA_SyRT_R | TCGGGATCTCTCCGGTTTT | *O. sanctum* transcriptome data (Rastogi et al., 2014) |
| 6 | Lipid.trans.prot_SyRT_F | GCTGCCATGTCATGCAACAC | *O. sanctum* transcriptome data (Rastogi et al., 2014) |
| 7 | Lipid.trans.prot_SyRT_R | CCGCCGTACATCACGAAGTT | *O. sanctum* transcriptome data (Rastogi et al., 2014) |
| 8 | MAPK_SyRT_F | TGCCAGCCTCGGGTTTC | *O. sanctum* transcriptome data (Rastogi et al., 2014) |
| 9 | MAPK_SyRT_R | CGTAATGTGGGAGCTGCCTAA | *O. sanctum* transcriptome data (Rastogi et al., 2014) |
| 10 | MAPK2_SyRT_F | TGCGCCGGGAAATCC | *O. sanctum* transcriptome data (Rastogi et al., 2014) |
| 11 | MAPK2_SyRT_R | GGCAGCGCACCACGTTA | *O. sanctum* transcriptome data (Rastogi et al., 2014) |
| 12 | MAPKK_SyRT_F | CCCAAATCTGCCACCAAGTC | *O. sanctum* transcriptome data (Rastogi et al., 2014) |
| 13 | MAPKK_SyRT_R | TCGCGGTGGATGATTTTGT | *O. sanctum* transcriptome data (Rastogi et al., 2014) |
| 14 | MAPKK1_SyRT_F | AGAACTCCCCAATCTCCTCCTT | *O. sanctum* transcriptome data (Rastogi et al., 2014) |
| 15 | MAPKK1_SyRT_R | TCAGCAGTAAATCGCCATCGT | *O. sanctum* transcriptome data (Rastogi et al., 2014) |
| 16 | MAPKKK3_SyRT_F | CATTTCCTCGAGCCTTTGCT | *O. sanctum* transcriptome data (Rastogi et al., 2014) |
| 17 | MAPKKK3_SyRT_R | TTCTGCCCGAGTGCATTTC | *O. sanctum* transcriptome data (Rastogi et al., 2014) |
| 18 | MyB_SyRT_F | TCCGGCACTGCTCTACCACTA | *O. sanctum* transcriptome data (Rastogi et al., 2014) |
| 19 | MyB_SyRT_R | ACTTTCTCCCACATGTCGAACTC | *O. sanctum* transcriptome data (Rastogi et al., 2014) |
| 20 | MyB1_SyRT_F | CGGTGGGCCAACCATTT | *O. sanctum* transcriptome data (Rastogi et al., 2014) |
| 21 | MyB1_SyRT_R | CTCTTCCTCCGGCGAGAAG | *O. sanctum* transcriptome data (Rastogi et al., 2014) |
| 22 | MyB2_SyRT_F | AGGATGATAGGTTGATTGCCTACAT | *O. sanctum* transcriptome data (Rastogi et al., 2014) |
| 23 | MyB2_SyRT_R | GCGGCCTTGGGAAGAGA | *O. sanctum* transcriptome data (Rastogi et al., 2014) |
| 24 | MyB4_SyRT_F | GGCGAACCGACAACGAGAT | *O. sanctum* transcriptome data (Rastogi et al., 2014) |
| 25 | MyB4_SyRT_R | ATTCCACGGCTCACGAGTTT | *O. sanctum* transcriptome data (Rastogi et al., 2014) |
| 26 | MyBC05_SyRT_F | TCAAGAGAGGCAACATGTCTGATC | *O. sanctum* transcriptome data (Rastogi et al., 2014) |
| 27 | MyBC05_SyRT_R | CAACGACCACCTGTTCCCTAA | *O. sanctum* transcriptome data (Rastogi et al., 2014) |
| 28 | MyB_Mixta_SyRT_F | ATCCAAGCTCGTGTCCAACAT | *O. sanctum* transcriptome data (Rastogi et al., 2014) |
| 29 | MyB_Mixta_SyRT_R | GGCGGCGGTGGATGA | *O. sanctum* transcriptome data (Rastogi et al., 2014) |
| 30 | MyB_Mixta2_SyRT_F | CCCGATATCAAGAGAGGCAAGT | *O. sanctum* transcriptome data (Rastogi et al., 2014) |
| 31 | MyB_Mixta2_SyRT_R | CTAGAAGGGCATGGAGTCGAA | *O. sanctum* transcriptome data (Rastogi et al., 2014) |
| 32 | MyB-P1_SyRT_F | AGACCTCATCGTCAAGCTCCAT | *O. sanctum* transcriptome data (Rastogi et al., 2014) |
| 33 | MyB-P1_SyRT_R | CAACCGCCCAGCTATCAGA | *O. sanctum* transcriptome data (Rastogi et al., 2014) |
| 34 | MyC-GP_SyRT_F | GTTGCCTGGGAGGTCGTTT | *O. sanctum* transcriptome data (Rastogi et al., 2014) |
| 35 | MyC-GP_SyRT_R | AGAAGAGTTTGGTATCAGCAAGATGA | *O. sanctum* transcriptome data (Rastogi et al., 2014) |
| 36 | MYC-RP_SyRT_F | GGAAGACACTGGTCGCAACA | *O. sanctum* transcriptome data (Rastogi et al., 2014) |
| 37 | MYC-RP_SyRT_R | CAGATTTCCACGCGTTTGAGA | *O. sanctum* transcriptome data (Rastogi et al., 2014) |
| 38 | NaCa.excanger_SyRT_F | TTCCTGGCAGCCACATATCTC | *O. sanctum* transcriptome data (Rastogi et al., 2014) |
| 39 | NaCa.excanger_SyRT_R | CCCCGGTCCGAGGATCT | *O. sanctum* transcriptome data (Rastogi et al., 2014) |
| 41 | NAC2_SyRT_F | GGGACTTCCACCCGGATT | *O. sanctum* transcriptome data (Rastogi et al., 2014) |
| 42 | NAC2_SyRT_R | TGGTTGCGGAGGTAGTACACAA | *O. sanctum* transcriptome data (Rastogi et al., 2014) |
| 43 | PLC_SyRT_F | GCCACCACATTGCGAAGTT | *O. sanctum* transcriptome data (Rastogi et al., 2014) |
| 44 | PLC_SyRT_R | GGAGAAGAGGTAGTGGTGGAAGTC | *O. sanctum* transcriptome data (Rastogi et al., 2014) |
| 45 | PLD_SyRT_F | GGGTACGGACGTGCACTGT | *O. sanctum* transcriptome data (Rastogi et al., 2014) |
| 46 | PLD_SyRT_R | TCCTGAACAAAGCTTCCACCAT | *O. sanctum* transcriptome data (Rastogi et al., 2014) |
| 47 | Pro.transporter_SyRT_F | GGCGATGCTTCCCTTCTTC | *O. sanctum* transcriptome data (Rastogi et al., 2014) |
| 48 | Pro.transporter_SyRT_R | CAAGAGGAATGCACCCAAGTG | *O. sanctum* transcriptome data (Rastogi et al., 2014) |
| 49 | SmHSP.4797-5192_SyRT_F | TGAAGGCGAGCATGGAGAAC | *O. sanctum* transcriptome data (Rastogi et al., 2014) |
| 50 | SmHSP.4797-5192_SyRT_R | TTAACGGCGGACTTCTTAACCT | *O. sanctum* transcriptome data (Rastogi et al., 2014) |
| 51 | SmHSP-16.6CI_SyRT_F | GGCTTCAGGTTGCGGTTCTA | *O. sanctum* transcriptome data (Rastogi et al., 2014) |
| 52 | SmHSP-16.6CI_SyRT_R | CATGGCCGCCTTCACTTC | *O. sanctum* transcriptome data (Rastogi et al., 2014) |
| 53 | SmHSP-17.4BCII_SyRT_F | CAGAAGCTCGTCAGGAAATTCC | *O. sanctum* transcriptome data (Rastogi et al., 2014) |
| 54 | SmHSP-17.4BCII_SyRT_R | CGCACTTGGCGGTGATG | *O. sanctum* transcriptome data (Rastogi et al., 2014) |
| 55 | SmHSP-17.9CI_SyRT_F | CTTCGATCCATTCTCGTTGGA | *O. sanctum* transcriptome data (Rastogi et al., 2014) |
| 56 | SmHSP-17.9CI_SyRT_R | ACGGCGGTGGAGAAAGG | *O. sanctum* transcriptome data (Rastogi et al., 2014) |
| 57 | SmHSP-25.1CP_SyRT_F | GCCCGGCATGACAAGAGA | *O. sanctum* transcriptome data (Rastogi et al., 2014) |
| 58 | SmHSP-25.1CP_SyRT_R | GGCCTTCACGACGAGCAT | *O. sanctum* transcriptome data (Rastogi et al., 2014) |
| 59 | SmHSP-35.9_SyRT_F | AACAGAAGAGAAAAATTTGGAACCA | *O. sanctum* transcriptome data (Rastogi et al., 2014) |
| 60 | SmHSP-35.9_SyRT_R | CTCCCCCCACAATCATCATC | *O. sanctum* transcriptome data (Rastogi et al., 2014) |
| 61 | WRKY_SyRT_F | ACCGCCGACCCCAACT | *O. sanctum* transcriptome data (Rastogi et al., 2014) |
| 62 | WRKY_SyRT_R | TTGCCGATAATGGAGGAGATTG | *O. sanctum* transcriptome data (Rastogi et al., 2014) |
| 63 | WRKYI_SyRT_F | CCCACCTCTGCATCAAACAA | *O. sanctum* transcriptome data (Rastogi et al., 2014) |
| 64 | WRKYI_SyRT_R | GCAGTCGAGCTGACCGAAGT | *O. sanctum* transcriptome data (Rastogi et al., 2014) |
| 65 | WRKYII_SyRT_F | TCTAGGACCAGTTCCGCTACAAC | *O. sanctum* transcriptome data (Rastogi et al., 2014) |
| 66 | WRKYII_SyRT_R | CGAAACCCGACCCGAATC | *O. sanctum* transcriptome data (Rastogi et al., 2014) |
| 67 | ABA8'hyd_SyRT_F | CCCACCCGGCACCAT | *O. sanctum* transcriptome data (Rastogi et al., 2014) |
| 68 | ABA8'hyd_SyRT_R | GGGATTTTGGGAGTAGAGTTGGA | *O. sanctum* transcriptome data (Rastogi et al., 2014) |
| 69 | AP2_SyRT_F | AGGCGGCCCTCAGAATG | *O. sanctum* transcriptome data (Rastogi et al., 2014) |
| 70 | AP2_SyRT_R | CCACTTCCCCCACGTTCTCT | *O. sanctum* transcriptome data (Rastogi et al., 2014) |
| 71 | ATPDNAPIF1_SyRT_F | GTTATGGAGGGACAGGAAAAACA | *O. sanctum* transcriptome data (Rastogi et al., 2014) |
| 72 | ATPDNAPIF1_SyRT_R | CAATGTGTCCTTTGCTCCTCAA | *O. sanctum* transcriptome data (Rastogi et al., 2014) |
| 73 | Betaine_ald_SyRT_F | AGGTGTGAGCGGCTGACTAAG | *O. sanctum* transcriptome data (Rastogi et al., 2014) |
| 74 | Betaine_ald_SyRT_R | GCATGGTTGCGAGCAGTTC | *O. sanctum* transcriptome data (Rastogi et al., 2014) |
| 75 | bZIP_SyRT_F | CCTCAACTTCGCCTCTGAAAA | *O. sanctum* transcriptome data (Rastogi et al., 2014) |
| 76 | bZIP_SyRT_R | GATCTTAGGCGGTCGGTTAGC | *O. sanctum* transcriptome data (Rastogi et al., 2014) |
| 77 | CaCmPk_SyRT_F | TTCCGGCGGTGAGCTTT | *O. sanctum* transcriptome data (Rastogi et al., 2014) |
| 78 | CaCmPk_SyRT_R | AGCAGCCTCAGCTTCGTTGT | *O. sanctum* transcriptome data (Rastogi et al., 2014) |
| 79 | cdka1_SyRT_F | ACAGGGCTCCAGAAATACTCCTT | *O. sanctum* transcriptome data (Rastogi et al., 2014) |
| 80 | cdka1_SyRT_R | CGACCGACCACACATCCA | *O. sanctum* transcriptome data (Rastogi et al., 2014) |
| 81 | cdkI3_SyRT_F | GCGCCGGTCGAGCAT | *O. sanctum* transcriptome data (Rastogi et al., 2014) |
| 82 | cdkI3_SyRT_R | GAGCATCCGTGCACATCCTT | *O. sanctum* transcriptome data (Rastogi et al., 2014) |
| 83 | cdkR_SyRT_F | TTTTGATGATACATACGAATACAGACATG | *O. sanctum* transcriptome data (Rastogi et al., 2014) |
| 84 | cdkR_SyRT_R | GGCGATTCTTGGGAAGCAAT | *O. sanctum* transcriptome data (Rastogi et al., 2014) |
| 85 | cdPK1_SyRT_F | CCGACAATGATGGGAAGGTAA | *O. sanctum* transcriptome data (Rastogi et al., 2014) |
| 86 | cdPK1_SyRT_R | AGCCAGCTGAGAACCCACTTT | *O. sanctum* transcriptome data (Rastogi et al., 2014) |
| 87 | Chaperonin21_SyRT_F | GAGAAGCCTTCGATTGGAACTG | *O. sanctum* transcriptome data (Rastogi et al., 2014) |
| 88 | Chaperonin21_SyRT_R | TGTTGCCCTCCTCATCAAGAG | *O. sanctum* transcriptome data (Rastogi et al., 2014) |
| 89 | cold_sip_SyRT_F | AGCCGGAACACGGACAGA | *O. sanctum* transcriptome data (Rastogi et al., 2014) |
| 90 | cold_sip_SyRT_R | CGCCTCCGATCTTATCTTTGA | *O. sanctum* transcriptome data (Rastogi et al., 2014) |
| 91 | COR_SyRT_F | TGCTGCAGGAACACATTCGA | *O. sanctum* transcriptome data (Rastogi et al., 2014) |
| 92 | COR_SyRT_R | AGAGGCCACTGCTCTTTGTGA | *O. sanctum* transcriptome data (Rastogi et al., 2014) |
| 93 | CRBF_SyRT_F | TGAACTTCGCCGACTCAGTGT | *O. sanctum* transcriptome data (Rastogi et al., 2014) |
| 94 | CRBF_SyRT_R | CGAAGAACCTTCGCGTCAGT | *O. sanctum* transcriptome data (Rastogi et al., 2014) |
| 95 | Cu_chaper_SyRT_F | ACTGCCTTCTGGGAAACTGAAG | *O. sanctum* transcriptome data (Rastogi et al., 2014) |
| 96 | Cu_chaper_SyRT_R | CTGGCGCTGGTGTGTTTG | *O. sanctum* transcriptome data (Rastogi et al., 2014) |
| 97 | DEAD_Box_SyRT_F | CGAACACTGGCTTTGCGTTA | *O. sanctum* transcriptome data (Rastogi et al., 2014) |
| 98 | DEAD_Box_SyRT_R | CCTGTTGTGTTCTGTCACCATGA | *O. sanctum* transcriptome data (Rastogi et al., 2014) |
| 99 | Dehyd_indP_SyRT_F | GCCTCGAAGCGCTACCAAT | *O. sanctum* transcriptome data (Rastogi et al., 2014) |
| 100 | Dehyd_indP_SyRT_R | GCATCAAAGCTATCCCATTTCAT | *O. sanctum* transcriptome data (Rastogi et al., 2014) |
| 101 | Dehydrin1_SyRT_F | CGAGTTCGATGAAAAAGCTAAGG | *O. sanctum* transcriptome data (Rastogi et al., 2014) |
| 102 | Dehydrin1_SyRT_R | CTAGAGCTTTTGGATCGGTGAAG | *O. sanctum* transcriptome data (Rastogi et al., 2014) |
| 103 | Dehydrin2_SyRT_F | GGAGGGAGAGAAACCGAACAT | *O. sanctum* transcriptome data (Rastogi et al., 2014) |
| 104 | Dehydrin2_SyRT_R | CCTGTCCGGCCTGTTCAT | *O. sanctum* transcriptome data (Rastogi et al., 2014) |
| 105 | Dble_WRKY_SyRT_F | CTTCAGCAGACCCCAAAGCT | *O. sanctum* transcriptome data (Rastogi et al., 2014) |
| 106 | Dble_WRKY_SyRT_R | CCAGCAGGAATTTCGTGGTT | *O. sanctum* transcriptome data (Rastogi et al., 2014) |
| 107 | Drought_indP_SyRT_F | AGGGCATCGGGAAGTACGT | *O. sanctum* transcriptome data (Rastogi et al., 2014) |
| 108 | Drought_indP_SyRT_R | GCCTGCATGAGAATCGTGGTA | *O. sanctum* transcriptome data (Rastogi et al., 2014) |
| 109 | EF1_alpha_SyRT_F | GCCCATGGTGGTGGAGACT | *O. sanctum* transcriptome data (Rastogi et al., 2014) |
| 110 | EF1_alpha_SyRT_R | CGCATATCACGAACAGCAAAG | *O. sanctum* transcriptome data (Rastogi et al., 2014) |
| 111 | EF1_gamma_SyRT_F | GAAAATACCGTGTCATTCGTCACT | *O. sanctum* transcriptome data (Rastogi et al., 2014) |
| 112 | EF1_gamma_SyRT_R | GCGGGCAATGTCCATACG | *O. sanctum* transcriptome data (Rastogi et al., 2014) |
| 113 | ERF1_SyRT_F | TCCGAGCCCCAGCCTATAA | *O. sanctum* transcriptome data (Rastogi et al., 2014) |
| 114 | ERF1_SyRT_R | TCTGTAGCGCGTCCCTCTCT | *O. sanctum* transcriptome data (Rastogi et al., 2014) |
| 115 | Ferritin_SyRT_F | CCAGCGTCTCTCAAGCATCTC | *O. sanctum* transcriptome data (Rastogi et al., 2014) |
| 116 | Ferritin_SyRT_R | TGATGGCGGCCTCACAGT | *O. sanctum* transcriptome data (Rastogi et al., 2014) |
| 117 | HSP70_SyRT_F | TCCGTCGTACGTCGGTTTC | *O. sanctum* transcriptome data (Rastogi et al., 2014) |
| 118 | HSP70_SyRT_R | GCGACCTGATTTTTAGCAGCAT | *O. sanctum* transcriptome data (Rastogi et al., 2014) |
| 119 | Hypoxia_resp_SyRT_F | CATTGACTCTTGCTGCCTTAGCT | *O. sanctum* transcriptome data (Rastogi et al., 2014) |
| 120 | Hypoxia_resp_SyRT_R | TTGGCACCGGACTTGTGA | *O. sanctum* transcriptome data (Rastogi et al., 2014) |
| 121 | Inducer_CBF_SyRT_F | AGAGGAAGGGCGATGACATG | *O. sanctum* transcriptome data (Rastogi et al., 2014) |
| 122 | Inducer_CBF_SyRT_R | AACAACTGATCATCCGAATCATAGTT | *O. sanctum* transcriptome data (Rastogi et al., 2014) |
| 123 | K.channel_SyRT_F | CCCTCGGCGCCAGAA | *O. sanctum* transcriptome data (Rastogi et al., 2014) |
| 124 | NAC1_SyRT_F | CGCCGGCCACCCTATC | *O. sanctum* transcriptome data (Rastogi et al., 2014) |
| 125 | NAC1_SyRT_R | TGGGTCGAATTTGTAGAGATCGA | *O. sanctum* transcriptome data (Rastogi et al., 2014) |
| 126 | PAL_OS_NGS_F | TCCCAACGAGTTCACATCTTGAT | *O. sanctum* transcriptome data (Rastogi et al., 2014) |
| 127 | PAL_OS_NGS_R | TTGGCTAATCCCGTGACTA ACC | *O. sanctum* transcriptome data (Rastogi et al., 2014) |
| 128 | C4H_NGS_F | TTGGTCCGGGAGTCCAAATA | *O. sanctum* transcriptome data (Rastogi et al., 2014) |
| 129 | C4H_NGS_R | TCACGGCCTGAAGGTATGG | *O. sanctum* transcriptome data (Rastogi et al., 2014) |
| 130 | 4CL_NGS_F | TTTGCGAATGCCAAACACA | *O. sanctum* transcriptome data (Rastogi et al., 2014) |
| 131 | 4CL_NGS_R | TGGGCAGGGTTATGGAATGA | *O. sanctum* transcriptome data (Rastogi et al., 2014) |
| 132 | CCOMT_NGS_F | ATGGCAGAAAATGGTGAGCAGCAAA | *O. sanctum* transcriptome data (Rastogi et al., 2014) |
| 133 | CCOMT_NGS_R | TCAGATGATGCGGCGACACAGG | *O. sanctum* transcriptome data (Rastogi et al., 2014) |
| 134 | C3H_OS_F | CTCCTTCCCGCCATTTTCCTCCTC | GenBank Accession no. HM990156 |
| 135 | C3H_OS_R | CGCCATTTACAAGTCCACAGCAATACGCT | GenBank Accession no. HM990156 |
| 136 | CS3H_NGS_F | GCCCGAGAGGTTCCTTGAG | *O. sanctum* transcriptome data (Rastogi et al., 2014) |
| 137 | CS3H_NGS_R | TCCTTCCAGCACCAAATGG | *O. sanctum* transcriptome data (Rastogi et al., 2014) |
| 138 | CAD_NGS_F | ATGGGCAGTTTGGAAGTG | *O. sanctum* transcriptome data (Rastogi et al., 2014) |
| 139 | CAD_NGS_R | TCACTGATAAAGCTTGCTCC | *O. sanctum* transcriptome data (Rastogi et al., 2014) |
| 140 | CCR_NGS_F | GCGGCTGAGGCCAAAGT | *O. sanctum* transcriptome data (Rastogi et al., 2014) |
| 141 | CCR_NGS_R | GATCCATGTAGATTGCACCGATT | *O. sanctum* transcriptome data (Rastogi et al., 2014) |
| 142 | Actin_OS_F | AGATTCCTCCAGCAAATCTTTCTC | *O. sanctum* transcriptome data (Rastogi et al., 2014) |
| 143 | Actin_OS_R | CTTTCTGGTGGAACAGCATCAA | *O. sanctum* transcriptome data (Rastogi et al., 2014) |
| 144 | CDS_30339_Unigene_49530_F | CGGTTTTCAGAATTGAGGATTACA | Present investigation COLD stress transcript |
| 145 | CDS_30339_Unigene_49530_R | TCAGAATTCGATTCCACCAAGA | Present investigation COLD stress transcript |
| 146 | CDS_31178_Unigene_51816_F | TGGCAGTCAACCGGTCTTTAC | Present investigation COLD stress transcript |
| 147 | CDS_31178_Unigene_51816_R | ATCGGCTCCACTTGGTTTATTAGT | Present investigation COLD stress transcript |
| 148 | CDS_30375_Unigene_49627_F | GTGGCTGCTGCTGGACATTA | Present investigation COLD stress transcript |
| 149 | CDS_30375_Unigene_49627_R | GTGATGATACCATTAACGACTCCAA | Present investigation COLD stress transcript |
| 150 | CDS_5730_Unigene_11635_F | GCAGAGTTAGGAAACATAGCTGCAT | Present investigation COLD stress transcript |
| 151 | CDS_5730_Unigene_11635_R | GCCAGATACGTCCCAGCAA | Present investigation COLD stress transcript |
| 152 | CDS_8224_Unigene_16350_F | GAGCTCATGTCCCTTCTCGAA | Present investigation COLD stress transcript |
| 153 | CDS_8224_Unigene_16350_R | CCCTTTCTCCCACCAAAAGC | Present investigation COLD stress transcript |
| 154 | CDS_13778_Unigene_24943_F | GCGGAGGTTACGGGATGTT | Present investigation COLD stress transcript |
| 155 | CDS_13778_Unigene_24943_R | TCGCGTCGACTATGTGATCAG | Present investigation COLD stress transcript |
| 156 | CDS_10777_Unigene_20262_F | GTTTGAGGCATTGAGGAAAGAGA | Present investigation COLD stress transcript |
| 157 | CDS_10777_Unigene_20262_R | TTGCTATCTGCCATCTTACTCAGTTT | Present investigation COLD stress transcript |
| 158 | CDS_31087_Unigene_51711_F | GAAAATGAAGGCGTGGTCTCA | Present investigation COLD stress transcript |
| 159 | CDS_31087_Unigene_51711_R | TCCGACACGAGCTCCAACA | Present investigation COLD stress transcript |
| 160 | CDS_18226_Unigene_31486_F | TGGCTGCCATTGTTGATGAG | Present investigation COLD stress transcript |
| 161 | CDS_18226_Unigene_31486_R | TGAACATGCATTGTGTGTTGGA | Present investigation COLD stress transcript |
| 162 | CDS_3941_Unigene_7884_F | AATGCAAGGTCGAGAAAAATTCA | Present investigation DROUGHT stress transcript |
| 163 | CDS_3941_Unigene_7884_R | CAGTGATGTTGGGATAGGTTCCT | Present investigation DROUGHT stress transcript |
| 164 | CDS_8563_Unigene_15857_F | GCGTCCTTGTCCTTGAGCAT | Present investigation DROUGHT stress transcript |
| 165 | CDS_8563_Unigene_15857_R | AGATCGGTTCCACCAGATTCA | Present investigation DROUGHT stress transcript |
| 166 | CDS_13123_Unigene_22628_F | AGCTCCGTTCCTACCCTCTCA | Present investigation DROUGHT stress transcript |
| 167 | CDS_13123_Unigene_22628_R | TGGTCGGGCGCTGGTAT | Present investigation DROUGHT stress transcript |
| 168 | CDS_5336_Unigene_10313_F | AGAATTCTCAAAACAGCATTTCACA | Present investigation DROUGHT stress transcript |
| 169 | CDS_5336_Unigene_10313_R | AATGACAATCAGATGGGAAACGT | Present investigation DROUGHT stress transcript |
| 170 | CDS_109_Unigene_262_F | ACAGGTTGGATCCGACTTCAA | Present investigation DROUGHT stress transcript |
| 171 | CDS_109_Unigene_262_R | CGTTGCAAGAGAGCTGTTTTAAATT | Present investigation DROUGHT stress transcript |
| 172 | CDS_17616_Unigene_29085_F | CTGAGTGGTGGAACCTAGTAACCA | Present investigation DROUGHT stress transcript |
| 173 | CDS_17616_Unigene_29085_R | TGGTGCTTGCTCAGCCAATA | Present investigation DROUGHT stress transcript |
| 174 | CDS_32039_Unigene_49003_F | GATCGGAAACGGAGTCTTGCT | Present investigation DROUGHT stress transcript |
| 175 | CDS_32039_Unigene_49003_R | TCCCGGCAAAGGATCCA | Present investigation DROUGHT stress transcript |
| 176 | CDS_1099_Unigene_2369_F | CAATGAAATGGTACGCAGTGGTA | Present investigation DROUGHT stress transcript |
| 177 | CDS_1099_Unigene_2369_R | CGTTTCCGGC CACGTAAG | Present investigation DROUGHT stress transcript |
| 178 | CDS_1248_Unigene_2842_F | ATTTCGGCGGCATTTCTCT | Present investigation FLOOD stress transcript |
| 179 | CDS_1248_Unigene_2842_R | CATCTCCGCTGCAAATTCTCA | Present investigation FLOOD stress transcript |
| 180 | CDS_11465_Unigene_20544_F | TATCGGGAAACTGTCAATGATGA | Present investigation FLOOD stress transcript |
| 181 | CDS_11465_Unigene_20544_R | TGGACCGGTGAGCTGGTT | Present investigation FLOOD stress transcript |
| 182 | CDS_13332_Unigene_23307_F | CGCGAAACTCCCTCCAAA | Present investigation FLOOD stress transcript |
| 183 | CDS_13332_Unigene_23307_R | TTTGAGCTTGTGTTGCTGAGAGA | Present investigation FLOOD stress transcript |
| 184 | CDS_15913_Unigene_27149_F | CGTTGACCGCCATGATCAC | Present investigation FLOOD stress transcript |
| 185 | CDS_15913_Unigene_27149_R | CCGCGAGCTAGCATGATGA | Present investigation FLOOD stress transcript |
| 186 | CDS_23_Unigene_52_F | TTGGGATGGTTTGCACTTCA | Present investigation SALT stress transcript |
| 187 | CDS_23_Unigene_52_R | GCAACGCTGGCGGAAGTA | Present investigation SALT stress transcript |
| 188 | CDS_2064_Unigene_4074_F | AGCACGACGGAGATAGCGTTA | Present investigation SALT stress transcript |
| 189 | CDS_2064_Unigene_4074_R | CGTGAAGATGAAGTTGCCCTAA | Present investigation SALT stress transcript |
| 190 | CDS_1748_Unigene_3559_F | GCGGTGGAGACAAACAACCT | Present investigation SALT stress transcript |
| 191 | CDS_1748_Unigene_3559_R | CCGACGTACTCCTCACACGAT | Present investigation SALT stress transcript |
| 192 | CDS_26744_Unigene_43881_F | TGCCACGCACCGTGTTC | Present investigation SALT stress transcript |
| 193 | CDS_26744_Unigene_43881_R | CCCTCGGCTTAGGTTTTGACT | Present investigation SALT stress transcript |
| 194 | CDS_19742_Unigene_31773_F | ACCTCGGCCGCTACAAAAC | Present investigation SALT stress transcript |
| 195 | CDS_19742_Unigene_31773_R | ACCAGCATCCCCAGAAAGG | Present investigation SALT stress transcript |
| 196 | CDS_243_Unigene_459_F | TGCCCTTTTTCGCAGATTTG | Present investigation SALT stress transcript |
| 197 | CDS_243_Unigene_459_R | CGCCGCAACGCTCAA | Present investigation SALT stress transcript |
