# Supplementary material for: Ocimum metabolomics in response to abiotic stresses: Cold, flood, drought and salinity
Source: PLoS One. 2019 Feb 6;14(2):e0210903. doi: 10.1371/journal.pone.0210903 (PMC6364901; doi:10.1371/journal.pone.0210903)
Supplement: S2 Table — (DOCX) [file pone.0210903.s009.docx]

**S2 Table. Statistical summary of RNA sequencing and Assembly**

|  | **CONTROL** | **COLD** | **DROUGHT** | **FLOOD** | **SALT** |
| --- | --- | --- | --- | --- | --- |
| **Statistical Summary of RNA seq** | | | | | |
| No. of reads | 36,072,101 | 39,107,564 | 28,431,775 | 34,968,694 | 26,784,969 |
| No. of bases | 5,431,476,645 | 5,890,619,093 | 4,304,268,054 | 5,265,397 | 4,054,867,908 |
| Total data in Gb | 5.43 | 5.89 | 4.3 | 5.26 | 4.05 |
| **Assembly Summary** | | | | | |
| No. of transcripts | 76,049 | 64,894 | 68,251 | 65,566 | 53,702 |
| Total transcript length (bases) | 81,134,796 | 59,706,350 | 66,987,941 | 69,780,486 | 41,689,045 |
| N50 | 1,683 | 1,471 | 1,530 | 1,713 | 1,137 |
| Maximum transcript length | 9,563 | 9,089 | 7,817 | 13,338 | 7,233 |
| Minimum transcript length | 201 | 201 | 201 | 210 | 201 |
| Mean transcript length | 1,066 | 920 | 981 | 1,064 | 776 |
