# Supplementary material for: Ocimum metabolomics in response to abiotic stresses: Cold, flood, drought and salinity
Source: PLoS One. 2019 Feb 6;14(2):e0210903. doi: 10.1371/journal.pone.0210903 (PMC6364901; doi:10.1371/journal.pone.0210903)
Supplement: S3 Table — (DOCX) [file pone.0210903.s010.docx]

**S3 Table. Summary of Unigene and CDS statistics**

|  | **CONTROL** | **COLD** | **DROUGHT** | **FLOOD** | **SALT** |
| --- | --- | --- | --- | --- | --- |
| **Summary of Unigene statistics** | | | | | |
| No. of transcripts | 64,603 | 55,891 | 58,018 | 55,956 | 46,235 |
| Total transcript length (bases) | 64,996,770 | 48,399,386 | 53,890,742 | 55,576,645 | 34,994,323 |
| N50 | 1,629 | 1,404 | 1,484 | 1,639 | 1,131 |
| Maximum transcript length | 9,563 | 9,089 | 7,817 | 13,338 | 7,233 |
| Minimum transcript length | 201 | 201 | 201 | 201 | 210 |
| Mean transcript length | 1,006 | 865 | 928 | 993 | 756 |
| **Summary of CDS statistics** | | | | | |
| No. of CDS | 40,560 | 32,731 | 36,502 | 34,650 | 27,811 |
| Total CDS length (bases) | 39,565,842 | 29,986,521 | 34,387,218 | 35,706,219 | 21,710,463 |
| Maximum CDS length | 9,018 | 7,137 | 7,542 | 12,954 | 4,152 |
| Minimum CDS length | 297 | 297 | 297 | 297 | 297 |
| Mean CDS length | 975 | 916 | 942 | 1,030 | 780 |
