# Supplementary material for: Ocimum metabolomics in response to abiotic stresses: Cold, flood, drought and salinity
Source: PLoS One. 2019 Feb 6;14(2):e0210903. doi: 10.1371/journal.pone.0210903 (PMC6364901; doi:10.1371/journal.pone.0210903)
Supplement: S4 Table — (DOCX) [file pone.0210903.s011.docx]

**S4 Table.** Commonly Upregulated and downregulated transcripts in all the four abiotic stresses (Cold, Flood, Drought and Salt)

| **UPREGULATED** | | |
| --- | --- | --- |
| **S.No.** | **Transcripts** | **Sequence_Description** |
| 1 | CDS_721_Unigene_1749* | F-box At3g07870-like |
| 2 | CDS_39398_Unigene_61545* | cation H(+) antiporter 18-like |
| 3 | CDS_22436_Unigene_36554 | kynurenine--oxoglutarate transaminase 1-like |
| 4 | CDS_28478_Unigene_44904 | glucan endo-1,3-beta-glucosidase 3 |
| 5 | CDS_34668_Unigene_53753* | PREDICTED: BRCA1-associated protein |
| 6 | CDS_39667_Unigene_62026 | PREDICTED: uncharacterized protein LOC105177651 |
| 7 | CDS_32387_Unigene_50467* | potassium transporter 6-like isoform X2 |
| 8 | CDS_12972_Unigene_23286 | 2-oxoglutarate malate translocator |
| 9 | CDS_18101_Unigene_30530 | PREDICTED: uncharacterized protein LOC105171345 |
| 10 | CDS_12160_Unigene_22167* | serine acetyltransferase 5-like |
| 11 | CDS_6781_Unigene_13621 | scarecrow 14 |
| 12 | CDS_3510_Unigene_7685 | trafficking particle complex subunit 2 |
| 13 | CDS_2772_Unigene_6354* | EIN3-binding F-box 1 |
| 14 | CDS_21223_Unigene_34825* | probable phosphatase 2C 49 |
| 15 | CDS_2079_Unigene_4705* | zeaxanthin chloroplastic-like |
| 16 | CDS_9187_Unigene_17758 | pre-mRNA-splicing factor 38B-like |
| 17 | CDS_25256_Unigene_40366* | E3 ubiquitin- ligase XBAT34 isoform X2 |
| 18 | CDS_16786_Unigene_28744* | stem-specific TSJT1-like |
| 19 | CDS_34870_Unigene_54054 | PREDICTED: uncharacterized protein LOC105955154 |
| 20 | CDS_13730_Unigene_24334* | sodium transporter HKT1-like |
| 21 | CDS_1214_Unigene_2685 | PREDICTED: uncharacterized protein LOC105167009 |
| 22 | CDS_123_Unigene_296 | ocs element-binding factor 1-like |
| 23 | CDS_18118_Unigene_30556* | aspartic ase 2 isoform X2 |
| 24 | CDS_4696_Unigene_10048 | elicitor-responsive 3-like |
| 25 | CDS_963_Unigene_2236* | lipase-like PAD4 |
| 26 | CDS_39892_Unigene_62731* | F-box PP2-B10-like |
| 27 | CDS_14008_Unigene_24746* | cyclic nucleotide-gated ion channel 2-like |
| 28 | CDS_12189_Unigene_22188* | 26S proteasome non-ATPase regulatory subunit 2 homolog A-like |
| 29 | CDS_5853_Unigene_12054 | PREDICTED: uncharacterized protein LOC105970915 |
| 30 | CDS_1678_Unigene_3880 | PREDICTED: uncharacterized protein LOC105156082 |
| 31 | CDS_39313_Unigene_61024* | UDP-glycosyltransferase 73D1 |
| 32 | CDS_38924_Unigene_60033* | cytosolic sulfotransferase 12-like |
| 33 | CDS_6330_Unigene_12818* | vacuolar amino acid transporter 1 |
| **DOWNREGULATED** | | |
| **S.No.** | **Transcripts** | **Sequence_Description** |
| 1 | CDS_11592_Unigene_21315 | PREDICTED: midasin |
| 2 | CDS_27156_Unigene_43001 | PREDICTED: uncharacterized protein LOC105157989 |
| 3 | CDS_26797_Unigene_42479* | coiled-coil domain-containing 150 |
| 4 | CDS_20547_Unigene_33835* | flavanone 3-dioxygenase-like |
| 5 | CDS_1507_Unigene_3270* | methylthioribose kinase |
| 6 | CDS_37441_Unigene_57434 | ribonucleoside-diphosphate reductase small chain-like |
| 7 | CDS_24654_Unigene_39576* | GDSL esterase lipase At4g18970-like |

*Stress responsive transcripts
