# Supplementary material for: Ocimum metabolomics in response to abiotic stresses: Cold, flood, drought and salinity
Source: PLoS One. 2019 Feb 6;14(2):e0210903. doi: 10.1371/journal.pone.0210903 (PMC6364901; doi:10.1371/journal.pone.0210903)
Supplement: S5 Table — (DOCX) [file pone.0210903.s012.docx]

**S5 Table.** Sequences of the CDS discussed in the manuscript

| **Sequences of transcripts used for DGE validation using qRT-PCR** | |
| --- | --- |
| CDS_26744_Unigene_43881 | GCGCACGTGTGGCTGCAGTTCCGGCTGGCGTACGTGTCGAGGGAGTCGCTCGTCGTCGGCTGCGGGAAGCTCGTGTGGGACCCACGTGTCATCGCCGCTCACTACCTCCGCTCCCTCAAAGGCTTCTGGTTTGATGTTTTTGTCATTCTGCCCGTGCCTCAGGCAGCGTTCTGGTTAGTAGTGCCACGGTTGATCCGAGACGAGCAGATAAAGCTGATAATGACCATCCTCCTCCTCATCTTCTTGTTCCAGTTCCTGCCTAAGGTCTACCACAGCATTTGCTTGATGAGAAGAATGCAGAAAGTGACAGGCTACATCTTCGGCACAATTTGGTGGGGTTTTGGCCTCAATCTCATAGCATACTTCATCGCTTCTCACGTTGCTGGTGGATGCTGGTACGTTCTTGCCATACAGCGCGTAGCATCGTGTCTGAAGCAGCAGTGTGATAAAAGTCGAACTTGTAACCTGTCGCTGTCCTGCTCGGAGGAGGTCTGCTACCAGTTTCTGTTGGGAGCAGGGAATTTGGACACTCCCTGTGGAGGCAACACCACGGTCGTTAGGAAGCCGTTGTGTTTGGATGTCAACGGGCCATTTCAGTACGGTATATACAAGTGGGCTCTTCCCGTTGTCTCGAGCAATTCCGTGGCTGTGAAGATTCTGTACCCTATTTTTTGGGGATTGATGACTCTGAGCACTTTTGGCAATGATTTGGAGCCGACGAGTCACTGGTTGGAAGTGATGTTCAGCATATGCACTGTGCTTAGTGGCCTAATGCTCTTCACTTTGCTGATAGGTAACATTCAGGTGTTTCTGCATGCTGTTATGGCAAAGAAACGGAAGATGCAGCTGAGGTGCCGCGACATAGAGTGGTGGATGAAGCGGAGGCAGCTGCCGTCGGAGCTGAGGCAGAGAGTGCGGCGGTACGAACACCAACGATGGGCGACGTTGGGGTGTGACGATGAGATGGAATTGATTCAAGACTTGCCTGAAGGGCTCAGAAGGGACATCAAACGCTTTCTTTGCCTCGATTTGATTAAAAAGGTACCCATTTTCCACAGCTTGGATGACCTAATCCTCGACAACATATGCGACCGCGTTAAGCCTCTCATTTTCTCCAAAGATGAAAAGATCATCCGAGAAGGAGACCCGGTGCCACGCACCGTGTTCATCGTGCGTGGCCGCGTCAAGAGCAGTCAAAACCTAAGCCGAGGGATGGTGGCCACGAGCACACTTGAGCCGGGCGGCTACTTCGGGGACGAGCTTCTCTCGTGGTGCCTCCGCCGCCCTTTCGTCGACAGACTCCCGGCTTCATCGGCGGCGTTCACCTGCATCGAACCGACAGAAGCATTCGCTCTTGAAGCTCGCGATCTCAAGTACATAACCGATCACTTCCGCTACAAATTCGCCAACGAGCGGCTCACCCGCACGGCCCGATACTATTCCTCCAACTGGAGGACCTGGGCTGCGGTTAACATCCAGTTGGGTTGGCGCCGTTACATCGAGAGGACGAGACGGGCCATGAGCCACCAGGCCGTGGGCGATGACAGTCACCGGCTTCTCCGGCAGTACGCCGCCTTCTTCATGTCGATTAGGCCTCATGATCATCTTGAATGA |
| CDS_1748_Unigene_3559 | CTGGCGGTGGAGACAAACAACCTCCAAGGCTGGAAGTTGGTGCCTGAATCGTGTGAGGAGTACGTCGGCAACTACATGCTCGGCAAGCAGTACCGCCACGACTGTGAAGTGGTGGCGGACGCCGCCATTGAGTACGCCAAGAGCGTCAAACTCGTCGGCGACGGCAAGGATATTTGGGTGTTTGATATTGATGAAACCACGCTTTCTAATATTCCTTACTATGCCCGCTCTGATGTCCAATTTGGGGCAATAGCATACAACGACACAAAATTCAACGAGTGGGTGGCGGAAGGGACCGCGCCGGCGGTGCCGGCGATCCGCCGCCTCTACAAGACGGTGTCATCTCTTGGATTTAAAACCGTATTCATTTCGGGAACGGCCGACAGGTTCACACAAATAAGGATAAAAAATTTAAAGAAAGCTGGTTATAGCAACTGGGAAAAACTCATCCTCAAGGGAGAAAGTGATCATAGTTCAGCGGTGGACTACAAATCCGAGAAACGAACAGAGCTAGTGAACGAAGGTTACAGAATCGTGGGAAATATTGGAGATCAATGGAGTGATTTGATCGGCACTCAC |
| CDS_19742_Unigene_31773 | TGGAGAAAAACGAAATCCCCGCGGCGAAAAAAGAGCCGAATTACAGAGGAGTTAAGGCCATGCCTTTCGTCATAGGGAATGAGACGTTTGAGAAGCTTGGAACGATTGGAACATCGTCGAATTTGTTGGTTTATCTCACGACGGTTTTTAATATGAAGTCCATAACGGCTTCGAATGTGATCAACGTCTTCAACGGGACCTGCAACTTCGGAACGTTGGTCGGAGCTTTCGTCTCCGACACCTACCTCGGCCGCTACAAAACCCTCGGAATCGCCTCCATTTCATCCTTTCTGGGGATGCTGGTGCTAACAATGACGGCAACATTCCCGAGCCTCCGCCCGCCAAAATGCGGATCCGGTGACAACGATCAATGCATCGGCGCTTCATGGGGGCAGCTGATCTTCCTCTTCAGCGCATTCGTTTTCCTCGTGATGGGTGCGAGCGGGATTCGCCCTTGCAACCTGGCGTTCGGGGCGGACCAGTTCAACCCGAACACGGAGTCGGGCAAGAGGGGCGTGAACAGCTTCTTCAACTGGTACTACTTCACCTTCACCTTCGCGATGATGGTGTCGCTCACGGTGATCGTGTACGTGCAGACGAGCGTGAGCTGGGCCATCGGGCTGGGCATCCCGGCCTTCCTCATGTTCCTCTCCTGCTTCTTCTTCTTCGTGGGGACGCGGATTTATGTGATGGTGAAGCCACAGGGCAGCCCCATGACTAATGTGGTCAGAACGTTGGTTGTCGCGTTCAAGAAGAGGAAGCTGCAGCTGCCGGAACTCCCGCCAGAGTCGCTGTTTGATTACCATTCGCCGGATTCCGTCAACTCCAAGCTTGCTTACACTGATCAGCTCAGGTGTCTTAACAAAGCGGCCATCATAACGGCCTCCGACAAAATAAACGACGACGGATCAGCCGCCGACCCATGGAGCCTCTGCAGCGTGCAGCAAATCGAGGAGATCAAATGCGTCATCCGGGTGATCCCAATCTGGATCTCCGGCATCCTATACTACGTCGTTTTAGCCCAAATGCAGACCTACCTCGTCTTCCAAGCCCTCCAAACCGACCGCCGCCTCACCCGCGGCAGCACCTTCCAGATCCCGGCCGCCACCTACAACGTGTTCGCCATGATCAGCCTCACCATCTGGATCCCCGTCTACGACCGCCTCCTCGTCCCCCTCCTCAGGCGGATCACCGGCAAAGAGGAGGGCATCACCATGCTCCAGAGGATCGGCATCGGGCTGTTTCTCTCTATCTTTACTATGTTGATTTCGGGCGTTGTGGAGGCCCACAGGAGGAATCTGGCCCTCACTCGCCCGACAATAGGGGTGGTCCCCCACAAGGGGGCAATTTCCTCTATGTCGGGCAACTGGCTGATACCCCAGCTGGCGCTGGCTGGGGTGTCGGAGGCCTTCGCTGTGATCGGGCAGGTCGAGTTTTTCTACAAGCAGTTCCCCGAGAATATGAGGAGCTTCGCGGGGTCCTTTTTGTTTTGTGGGTTTGCGATTTCGAGCTATTTCACGAGCTTCCTGATCTCGGTCGTTCATAAGAAGTCGAGGGTCGGGGAGACGAGCAACTGGCTGGCGGAGGATTTGAATAAGGGGATGTTGGATTATTTTTTCTACCTTGTTGCGGGGTTGGAAGCGTTTAATTTGGGGTATTTTTTGCTTTGTTCGAAGTGGTATAGGTATAAGACTGTTGAGAGTAGACAAGTGGAGGTGAGTATGGAGAAGATCGATCCACAAAAATCTGTCTAG |
| CDS_243_Unigene_459 | CTCAGACTTATACGCACATGTGCCAAAATTCACTCACAAAAACACACACTCACACACACTTCAAGAAATCCTTCAATTCTCCGATGAAGGCTGAAGCTGCAGACGGTGAACTCTCCATCGGAATACCGCTTTTGGCCGGCAATGGTGCCGGCGGCGCGGCAGAATCTGCTCAACACGGCGGCCACCGCCACAACTCGGGCTCCACGTCTTTCTTCAAAACCTGCTTCAACGGCCTCAATGCTCTATCAGGCGTAGGCATTTTATCAGTGTCTTATGCACTATCGTCTGGTGGATGGTCAAGTCTGATGCTCCTCTTCCTCATCGCTGCCTGCACTTTCTACACCTCCTTACTAATCCAGAGATGCATGGATTTGGACTCCAACATAACCACCTACACCGACATCGGCGATCGAGCCTTTGGCCCCAAAGGCCGAGCCCTCGTCGCAGTAGCCATCAACGCGGAGCTCTATCTAGTCGCCACTGGTTTCCTAATCCTCGAAGGAGACAATCTCCACAGCCTTTACCCCGATTTTCATTACGAAATCGGGGGCTTCTTTATAGGCGGACGTGAAGGCTTCATCTTGATCGTCGGCCTCGTAGTTCTCCCCACTGTCTTGCTCGACAACATGAGCATCCTTTCCTACATCTCCGCTACTGGAGTTGTCTCTTCTTTTATACTAATCGCTTCCATTTTGTGGGCTGCTGTTTTCGACGAAATCGGGTTTACAAAACAGGGGACTCTGTTTAAATACAGAGGAATTCCCACTGCCGTTAGTTTGTTCGCATTTTGCTACTGCGCTCATGCAGTTTTCCCAACTTTGTACACTTCCATGGCCGACCAAAAGAAGTTCTCCAAGGTTATGGTGGTTTGCTTTGTGGTGGGGACGATGAGCTACGCTTCAATGGCGGTTTTAGGTTACCTGATGTTCGGCGATGAGGTTGAATCGCAAATCACATTGAATCTCCCGACAAATAGGGTTAGCTCGAAGGTGGCGATCTACACGACGTTGGTGAATCCGTTGGCGAAATACGCGTTGATGGTGACGCCGATTGTGGAGGTTGTGGAGAGGCGTTTTGTGAGGGTGCACCCTGTTTTGGTTAGGTGTGGTTTGGTGGTGAGCACTATTATGGTGGCGTTGGCGGTGCCCTTTTTCGCAGATTTGATGTCCCTTGTGGGCGCGTTTTTGAGCGTTGCGGCGTCGATTCTGCTGCCGGGCCTATGCTACGTGAAGATCTCCGGCATTCACCGGAGATTCGGGGCGGAGTTGGTGGTGTTGTGTGGGATTGTGTGTGTTGGGGGCGTTGTTCTTGTGGTCGGTACTTACACGGCTCTCTTGGAGATTCTTCATCATTATTTCGTTTGA |
| CDS_23_Unigene_52 | CGTCGATGAACAACGATCTACGGACAGCATTCAATAATCTCCAACAGGAGTTCCCCACTGTCACTATACTAGCTGCCGATTTCTACACTGCTGCAAGAGTAATCATTAATCAGACATCTGTTCTAGGTCCCGGTGGGAATCCGGCATTGAGATCTTGTTGTGGAATTGGAGGAAGATATAACTACGATAGCAAAAGGTTTTGTGGAAGCTTTAATGTGCCCGTTTGCCCTAACCCTAACAACTATGTCTATTGGGATGGTTTGCACTTCACCCAAGAAGCTTATCGTCGTATCGTCAACATGCTACTTCCGCCAGCGTTGCCACTACTCAACTGCACTACAACGTAA |
| CDS_2064_Unigene_4074 | GAGTGTTCAAAGTTTGGATGCATAACAACAAGAACACATTCGGTAAAAAAGACCAACCTCCGCCGCCGCAGCCGCCGCTGCCCAATTTATCTAACAGCACTACTGAAATCTCATCAGCGCCGGCGCCAACAACTAACAGTAACGGCGTTAACTATTGCGACGTGGGCGCCGCCGGCTTCCGCCACCAGCACGACGGAGATAGCGTTATGAAGGACGATGATGTTCGCAGAGAGGAATTAGGGCAACTTCATCTTCACGGTCATGCTCATGCTCTTGGTATTAATGGATCTTCTTCTTCTTCT |
| CDS_1099_Unigene_2369 | CCGGACTATTGCCCTTGCATGGAGATTCTCTACATATACGTAACTCCACGACACTCAACAATGCACCAAAAAAACATGTCATCCCTTCAATCTTCATGCACGGTGCTCTCCACCGACCCCAAACACCGGCTCTCCCCATTTCCACCCACCAAGCCCTCCCACCTCCTCCCCATCCCACGGAGGCGCCACCACCTCCACGTCTCATGCAGCGGCGGCGCCGACCAAAAACCCCCCTCTGAAGGAAAGCTCGACCGCCGAAACGTCCTCCTCGGCCTCGGAGGCCTCTACGGCGCCGCCAACCTCATCTCCGACCCCAAAGCCTACGCCGATCCCATTCAGCCACCCGATCTGAGCAAGTGCGGCCCCGCTTACGAAGGCAAAACCGGCGACCTCCTCGACGTCAACTGCTGCCCCCCCATCTCCTACAGCATCATAGACTACAAACTCCCGCCGGTCACCCACCTCCGCATTCGGCCGGCGGCGCACAAAATCGACCCACAGAGCTTAGCCAAGTATGAAAAAGCAGTCCGTCGCATGAAAGAGTTGGATAAGACCGATCCCAACGATCCACGTGGCTACACTCAGCAAGCCAATATTCACTGCGCTTACTGCAACAGCGCCTACAAGCAAGTCGGATATGAAGATAAGGACATATCCATTCATTATTCCTGGCTCTTCTTCCCCTTCCATAGATGGTATTTATATTTCTACGAGAGGATTCTGGGGAGTTTGATCGACGATCCCACCTTCGCCTTGCCCTTCTGGAACTGGGACAACCCTCCCGGCATGACGCTGCCGCGGATTTTCGACAACTTCAAATCCCCGCTCTACGATGAGAATCGAAACCCCTGCCACCGGCCGCCGGCGGTGGTCAATCTTGCCTTGAACAACGACATAACTGATGATATACAGAAGGTGACAAATAATCTGAGCGTAATGTACAATGAAATGGTACGCAGTGGTATATCGGCGACCGATTTCATGGGGCAGCCTTACGTGGCCGGAAACGATCACCCTCCGCCGCCGCAAGGAGGGACGTCGGAGAGAGGTTCCCACACCAGCGTCCACGCCTGGGTCGGAGATCCCGATAACCCTTACCATGAAGATTTGGGGAATTTCTACTCCGCCGGGAGAGACTCCGCCTTCTACTCCCATCACGCTAACGTCGACAGAATGTGGACGATTTGGCGGACTCTGAAATCTCCTCCCGCCGTGAAAGACATCAAAGATCCCGACTATCTGAACGCCGCCTTCTTATTCTACGACGAGAACAAGCAGCTCGTCCGCGTTAAGGTCGGCGACTGCGTCGACAACAGGGCGATGGGATACGACTACGAACAAGTCGATATTCCGTGGCTGAACTATACGCCGCCGAGGAGAGCGGCGAGAGCGAAGATCCAGGATATATCGAAAGGCGTGGAGAGCGCGAAGAAGATTTTCCCTCTGAAGCTGAACAAGATAACTAGGGTTTTGGTGTCGAAGACGAAGATCGGTAAAGCTGATGAAGTGCTGGTGCTGGAGGATATACAGACTGATCCGAAGCAGCTGATTAAGTTCGACGTGTACATCAACGATGAAGACGACGCGCCGAAGGAGGTGTACAAGGCGGAGTACGCGGGGACGTTCGCGCAGCTGCCGCACAGGGCGCACAGCATGAAGAGCAAGGGGTCGTTGAGCTTTGATCTGAAGGATGTGTACGAGGACATTGATATCGGCGACGATGATACGGTGGTGGTCACCATCGTTCCAAGATTCCGCCACCGTGATATTACCATTGGTGGCATCAAAATCATCGAGGCGCCGACTAAAGCCGCCGCTTAG |
| CDS_32039_Unigene_49003 | TGAAATCCCATGCTTTTTACTTCTTCCTTCTGCTACAGTTGTTCCCAATCACAGCCGTCGACGAGCCCGTGTTCACGGCCGCAGAACTTATTCTCCTCAACTGCGGCGCCGACGCACCCTCCGAAGACGTAAGTAATCGGAGCTGGGACAGTGACGACCGTTCGAAATACACTCCAACAAACGCCGCCGCCAGGTCTTCTACATCCACAGCTTTCCAAATGCACCCCGCCGTTTCTGCAGTCCCTTATCAGACGGCTCGTGTTTTCCGTTCCCCTTTCACCTACACTTTTCCGATCACCGCTGGCCAGAAATTCCTCCGCCTTTACTTCCACCCCGACACCTACTCCGACTACAACACCTCTCAATCTTTCTTCTCCGTCACCGCGAACGACTTCACTTTAGTGAGCAATTTCAGTGCTTTCCTTTATTCATTTAATTCTCCGGCACCCGCTTTCTCCAAGGAGTTCATCATCAATATTGACAGTTCTCAGACGCTGAGATTGATATTCTCCCCGAATCCGAACTCTTTTGCTTTCGTCAACGGAATCGAAATTGTTTCGATTCCGGATGAACTCTACTTCAGGGGAAACGATGTGCCAATAAAGAATGTCGATGCGAACAGTGTGACGTATCTAGAAAACAGTAAGGCCCTGGAAAATCTTTACCGGCTGAACGTTGGCGGCGGCCGCGGCGTTGCAATTCAGAATGACAGCGGGCCTACCCGCGGAATGTTTCGGGCCTGGTCCAGTGATTACGATTACATATTGGGCCCTGACTATGGTTACACTCCTCACCTTGACGTTCCGATCACCTACACGAGTAAAACTCGGTCCTACAGTGCTCCGCCAATAGTATACACAACTGCTAGAGTGACCGCAAATTACAGTGTTGATCTGCAGTGGTCCTTTCCCGTTGATTCTGGGTTTTCCTATCTCCTCAGATTCCATTTCTGTGAGTTCACATTGGACGTGACACAACAAAACCAGAGGGTGTTCACCATTTCTATCAATGGTGAGGTTGCGGAAATTCAAGTCGATGTGATAAAGTGGGCCGACGGCCCCAAGATTCCGATTTTCGTAGACTACATAAAAATGATTCCTGATGACGGCCGTCGCGGGAAGAAAAACCTGCAGCTATCTTTGACTCCCACCATGGAAACCAGTCCAGAATATCATTCGGCAATTTTGAACGGCTTAGAAATATTCAAACTCTGTGATCGGAAACGGAGTCTTGCTGCCGCCAACCCTGAACCGACGGTGGATCCTTTGCCGGGAGTAGAGATCCCGACACCCACGACGAAAAGTGGAGGATCTTCTGTGATTTACGCTGTCACAGGGAGTGTTATCGGGGTTCTTGCGGTGGTTGCAGCCGTGAGTTTCTTGATATTCCGGCGAAGGCGGAAAGCTGAGTCCACAAGTGTTGCAAACAAATTGTCGATGCCTGTCGATTTATGCAGATATTTCTCCATTGACGAGATCAAAACCGCCACCCGCAACTTCGACGACAGTTCTATCATCGGCAGGGGAGGATTCGGCAACGTCTACAAAGGGCTCATCGACAACGACGCCGCCACCGTCGCCATCAAAAGACTCAACCCATCTTCCAAACAAGGCGCTCACGAGTTCCTCACGGAAATCGGGATGCTGTCGAGGCTGCGCCACCTCCACTTAGTCTCACTCATGGGCTACTGCGACGGCGGCGGCGAGATGATCCTGGTCTATGACTACATGGCCCACGGCTCCTTACGCGACCATCTCTACAACTCGGAAAACTCGCCATTAACATGGAAGCAACGCCTCGAGATCTGCTTGGGTGCTGCGAAAGGACTGGACTATCTCCACACCGGCGGTAAGCACACCATCATCCACCGAGATGTCAAGTCCACCAACATCCTACTGGATGAGAAATGGGTGGCCAAGATGTCCGATTTCGGCATCTCCAAAGTTGTCCCCACAGCAGGCCTGGACACACCTGTCAGCACCGTCGTCAAAGGAAGCCTTGGTTACGTCGACCCGGAATACATCCGGATGCGACAGCTGACGAACAAGTCCGACGTGTACTCATTTGGGGTGGTGCTGTTCGAAGTTTTATGTGCAAGGCCCGTAATGATGCCGAGCCTACCAAGAGAGCAAGTGAACTTAGCAGAGTGGGCTAAATTCTGCTTCAGAAAAGGAACACTTGAACAGATAATTGATTCTAATCTCAGAGGCAAGATCGCACCAGAATGCTTGAGCAAATTTGCAGAAACAGCCGTAGCTTGCCTCAAGGACAGAGGGGTTGATCGACCATCAATGAACAACGTCGTATGGAGCCTGGAATTCGCAATGCAGCTCCAAGAATCAGCTGAGAGCACGAAGGGGAATGATACCGGCGATGGGTTCGCGGGCATGAGCCCACCGCTGCCACTGCTTGGGATTGATGAGTTTGACGCTACGGATGAAGAAGCTTCATTTACCAGATCTACCGAGCTTGCGGCATCAAAGAGCAGCGAATTCTCCACTGCCAGCGGTGAAAGACTCAAATCTGGCGATGTTTTCTCTGAGATTATCAACCCATTAGGACGATGA |
| CDS_17616_Unigene_29085 | TGGCTCTTGTATCCGAAGGAAGGTCGTCGCTGAATCCGAACGCGCCGCTATTCATTCCAGCGGCGGTGCGGCAGGTGGAGGACTTCTCGCCTGAGTGGTGGAACCTAGTAACCAGCGCCACTTGGTTCCGCGATTATTGGCTGAGCAAGCACCAGGAGGAGGATATTTTCGGGGAGGAGACAGATGGAAACGACGTCGTTGAGTTGCCTGATAACTTCGATCTCGGCGTCGATGAAGATATTCTGAATATGGAAGCTCAGTTTGAAGAATTTCTCCAGTCTGCTGAAGTTCATGACTACCATGGTGGTATGCCTGCCAGAGGAATTACTGAAACTGCTCATGGTAAGAACACCAGAGCCCTGGTGAAGAATCTGAGCATGCCGAAGGAGAGGGGCCTCGTGGGAATGAGGAATTGGGAGAAGCCAGCCAAGATTGTGAGCCCCAAGTGCACCCCCAGATTGATCCAGCAACCTCGTTGA |
| CDS_109_Unigene_262 | TGTCGTCGAGGGGAGGGACGCCGCAGCAAAATCCGCAGCTGCAGCGGCGGATTACGCGAACGCAGACGGTGGGGAATTTAGGGGAGTCGGTGTTTGACAGTGAAGTAGTGCCGTCGTCTTTGGTGGAAATCGCCCCCATTCTTCGAGTTGCCAATGAGGTGGAACCTAGCAATCCGCGTGTTGCCTACCTCTGTCGGTTTTATGCATTTGAGAAGGCTCACAGGTTGGATCCGACTTCAAGTGGACGCGGCGTTCGCCAATTTAAAACAGCTCTCTTGCAACGTCTAGAAAGGGAAAATGATCCTACC |
| CDS_13123_Unigene_22628 | ATCCATCAACTCACGGTGTTTACGAGGAAGGAATCGACGAGATATGCAAAATAGTCCACGACAACGGTGGCCAAATTTACATGGATGGAGCTAACATGAATGCTCAGGTTGGTCTAACGAGCCCCGGCTTTATCGGTGCTGATGTTTGCCATCTCAATCTTCACAAAACGTTCTGTATTCCTCATGGAGGAGGCGGGCCTGGGATGGGACCTATCGGCGTGAAGAAGCACCTAGCTCCGTTCCTACCCTCTCATCCGGTTGTGAGCACCGGAGGTATACCAGCGCCCGACCAGTCTCGGCCACTCGGCACAATCTCGGCCGCGCCTTGGGGATCGGCGCTCATATTGCCGATATCATACACGTACATTTCCATGATGGGATCAAAGGGCTTAACTGAAGCATCTATGACGGCCATCTTGAATGCAAACTACATGGCCAAGCGTCTCGAGAAGCACTACCCGATTCTCTTCCGTGGAGTGAACGGGACAGTGGCTCACGAGTTCATCGTAGATCTGAGAGGGTTTAAGAACACAGCTGGAATCGAGCCTGAAGACGTTGCCAAGCGTCTCATGGACTACGGATTTCATGGCCCCACAATGTCGTGGCCGGTTCCGGGGACGCTCATGATCGAACCAACTGAAAGTGAAAGCAAGGCCGAGCTAGACAGGTTCTGCGATGCTCTTATCTCTATTAGAGAGGAGATTTCTCAGATTGAGAAAGGAAAAGCTAACATCCACAACAATGTTTTGAAGGGAGCTCCTCATCCACCATCAGTGCTGATGGCCGATGATTGGACGAAACCGTACTCGCGGGAATACGCTGCCTACCCTGCCACGTGGCTCAAGACGGCCAAGTTCTGGCCGTCGACAGGGCGTATTGACAACGTGTATGGCGATCGCAAC |
| CDS_8563_Unigene_15857 | GCAACATGCATGATGCGTCCTTGTCCTTGAGCATTCATACAACTTGTGCTCGCTTGATGTTGAATCTGGTGGAACCGATCTTTGAGAAAGGTGTCGATCAGACATCAATGGATGAGGCACGGGTTTTATTGGGTCGAATATTGGATGCTTTTGTTGGAAAGTTTAACACCTTCAAGCGTACTATCCCTCAGCTATTACAAGAAGGAGAGGAGGGAAAAAATCGGTCTACTTTGAGGTCAAAACTTGAAGCGCCAGTACAGGCAGTGCTTAACTTGCCGATGTCTGTGGAGCATCCCAAGGAGGTCAATGATTGTAAGCATCTGATAAAAACGTTAGTTATGGGAATGAAGACAATAATATGGAGCATCACCCATGCACATATACCAAGATCACAGGTTTCACCCTCCACGCATGGTACTCCTCAGCAAGTGCTTGCGTCGACTTCATCAGGTTCTTCTGTACCTCAGCCTTTTAAGGGAATG |
| CDS_5336_Unigene_10313 | CAACTTCAACTTCTCGATATAGTTTCTCTGTTTCAGTCCTTGAGCTTTATAAAGATCAGATAACTGATTTGCTTCTGGAAACTGGAAGTGCTCAACCAAAGGTCGACATTGGGTCATTGGGTTGTGTTATAGAACTTGTCCAGGAAAAAGTTGAGAACTCTATGGAGTTCAACAGAATTCTCAAAACAGCATTTCACAATCGTGGAAGTGATACCTTGAAGTTTAACGTTTCCCATCTGATTGTCATTATACACATATACTATACAAACTTGATCACTGGCGAGAATATATACAGC |
| CDS_3941_Unigene_7884 | TAGGAATGCAAGGTCGAGAAAAATTCACCTTTGAGGATATGCTCTGTTTCCAGAAGGAACCTATCCCAACATCACTGCTGAAAATAAACAGTGATCTTATTAGTAGAGCAGTTAAACTATTCCAAGTTATCCTGAAGTATATCGGGGTTGATTCCTTGGATCGAGTTTCTCATATGAGCTTAGATGCACGCATTGAGCTTGTCAGCAAACTATACAAACACTCTTTGAAGTATTCTGAACTCCGAGATGAACTCTTTATGCAAGTCTCAAAGCAGACAAGAAATAATCCTGATAGACACTCTTTGGTTAGGGCATGGGAG |
| CDS_13123_Unigene_22628 | TATCCATCAACTCACGGTGTTTACGAGGAAGGAATCGACGAGATATGCAAAATAGTCCACGACAACGGTGGCCAAATTTACATGGATGGAGCTAACATGAATGCTCAGGTTGGTCTAACGAGCCCCGGCTTTATCGGTGCTGATGTTTGCCATCTCAATCTTCACAAAACGTTCTGTATTCCTCATGGAGGAGGCGGGCCTGGGATGGGACCTATCGGCGTGAAGAAGCACCTAGCTCCGTTCCTACCCTCTCATCCGGTTGTGAGCACCGGAGGTATACCAGCGCCCGACCAGTCTCGGCCACTCGGCACAATCTCGGCCGCGCCTTGGGGATCGGCGCTCATATTGCCGATATCATACACGTACATTTCCATGATGGGATCAAAGGGCTTAACTGAAGCATCTATGACGGCCATCTTGAATGCAAACTACATGGCCAAGCGTCTCGAGAAGCACTACCCGATTCTCTTCCGTGGAGTGAACGGGACAGTGGCTCACGAGTTCATCGTAGATCTGAGAGGGTTTAAGAACACAGCTGGAATCGAGCCTGAAGACGTTGCCAAGCGTCTCATGGACTACGGATTTCATGGCCCCACAATGTCGTGGCCGGTTCCGGGGACGCTCATGATCGAACCAACTGAAAGTGAAAGCAAGGCCGAGCTAGACAGGTTCTGCGATGCTCTTATCTCTATTAGAGAGGAGATTTCTCAGATTGAGAAAGGAAAAGCTAACATCCACAACAATGTTTTGAAGGGAGCTCCTCATCCACCATCAGTGCTGATGGCCGATGATTGGACGAAACCGTACTCGCGGGAATACGCTGCCTACCCTGCCACGTGGCTCAAGACGGCCAAGTTCTGGCCGTCGACAGGGCGTATTGACAACGTGTATGGCGATCGCAAC |
| CDS_15913_Unigene_27149 | ATGGCGCTTGAAGCTCTCAACTCTCCCACAACTCCGCCGCCGCCGCCGTTTCAATTCAGCACCGCCGCCTCCTACCTGGAGCCTTGGTCTAAAGGCAAGCGATCCAAGCGCCCTCGCAGCGTTGACCGCCATGATCACCCCACTGAAGAAGAGTACCTCGCTCTCTGCCTCATCATGCTAGCTCGCGGCGGCGCAGCCTCCACCTCCGCTGCCGCCAAGAACTTGCAGCCCCAAACCCACCTCCCACCACCGCCACCGCCGACGGATACTGCTAAGTTGGTCCACAAGTGCTCTGTTTGTAACAAGGCTTTCGGCTCCTACCAAGCGTTGGGCGGCCACAAGGCCAGCCACCGCAAGCTCGCCGGCGGTGGTGGTGACGAACAGTCCACCACCTCCGCCTCCGCCGTCACCTCGACCGCTTCCGGCAGAGGCGGCGGGAGGATCCACGAGTGCTCCATCTGCCACAAGTGCTTCCCCACTGATCAGGCCTTGGGGGGCCACAAGCGCCGCCACTACGAGGGCGCGGCGAGTTCTTCGGAGGGGGTTGGTTGTACCGTTAGGCATCGGGATTTCGACCTGAACTTGCCGGCTTTCCCTGATTTATGGCAGAGATTTGGCGGCAAAGACGAAGTTGAAAGCCCCCACCCGGCGAAGAAAGCACGTTTTTCGCCGGCGAAATTGGAAGTTGTTTGA |
| CDS_13332_Unigene_23307 | TGCAGTGGACTAAACTACGAGAAGCTCTCTTCGCAAGCGCTCGACCACCTCACACGCAACGCGAAACTCCCTCCAAAAACCGCGGCCCAAGCCATCGTCTCTCAGCAACACAAGCTCAAAAGCTTGCTTCAAGACATCAACACCGGGTCCTCATCCAGCCCTATCAACGAGGGCTCGAAGCAGATCGTGCTATACGCGAAGAAGTTCAATCTCACAGACGAAAACGAGAGGCTGAAAGCCCATCTTCAAGGCATGCAATGGAGAGTGGTGGAGTTGGAAAAAGTGTGCAGGAAAATGCAAGTGCAGATGGCAAAGATGATGAAATCAAGAATGGCAAACAATAG |
| CDS_11465_Unigene_20544 | CAGAAGACCCCATCTTCGAGAAAGCCGGCCGGTCAGGCTACATGACCGGCTCGATCTCCCCTGCCGTCTGCCGCCTCGACCGCCTCACCACCCTCGTCGTCGCCGACTGGAAGGGCATCTCCGGCCAGATCCCCTCCTGCATCACCTCCCTCCCCCACCTCAGAATCCTCGCCCTCGTCGGAAACAAAATCTCCGGCGAAATCCCCGCCGACATTGGCAAGCTCAGCCGCCTCACAGTCTTCAACGTCGCCGACAACCAAATCTCCGGTTCTCTGCCGCCCTCCATTGTTGAACTCAAGAGCTTAATGCACTTGGAGCTCAGCAACAACAAGCTCTCCGGCGAAATTCCGGCCGATATCGGGAAACTGTCAATGATGAGCCGGGCTTTGCTGGGACGGAACCAGCTCACCGGTCCAATCCCGAGTTCGCTAGCTAATATATATAGGCTGGCTGATTTGGACTTATCGATGAACCAG |
| CDS_1248_Unigene_2842 | TGGGAAAGAAGAGTGGACATTCTTGGTTAGATGCCGTCAAGAATGCTTTCCGATCTCCTCCTACCAAAGACGGTGACAAGAGGTGCAGCAGAAGAAGAGACGACAACGAACCGGAAGAAGAGGAAAAAAGAAGAGAAAAGAAGAAATGGTCATTTCGGCGGCATTTCTCTCTGGAGACGAAAATTCAGCACAACGTTGTGAGAATTTGCAGCGGAGATGAGGATGTTGTGAGGAAGGTGCAGCCCAAAATGGAGTATCCGGCGGCGTCGGAGCAGAGGAGTGCTTTTGAGGTGGCTATGGCG |
| CDS_10777_Unigene_20262 | AATTTAGCTCTACAGTTTGAGGCATTGAGGAAAGAGAATCAAACATTGCTTATCCAGGTGCAAAAACTGAGTAAGATGGCAGATAGCAATGATGTTGAGGAGATCGACAAGAACCAAATCAAATTAGATATTAATAGTCATGAGATATGCATGCCGTTGGGCGGCGAGGCGAACGGAAGAGTAGATTACTTGGAAGACGATGATGCTTTGAATATGGCTCAGATTGCTGAAAGTTCTTTAGCTTCACCTGAAAATGGATGTAGCTTAGCATCTTGTAATTTTCTTGAAAATACTGCACAGTGGTGGGAGTTCTAG |
| CDS_8224_Unigene_16350 | ACCAACAATCCATTTATTAATTTTCATGCTATAAACTTCCAACACAACTATATCGCTATAATGCACCCTCTCACTACACATCACTTGCACCAAATTTTCAAAGAGCTAGATAGAAATAAAGATGGCCTTGTTAGCATAGATGAGCTCATGTCCCTTCTCGAAAGGTCTGGCCTGCACGTTAGTCAAGTCGAGCTCGAGCTTTTGGTGGGAGAAAGGGCCCTCAACTCTTTTGATTTTCTATTCTTTTATCAATCTTTGATCAGCAAAAGTGTTGGAGATGAAAGAGTGGGAGCAAGTGATGAAATATTGGAGAGTGATCTTAGAAAAGCATTTAGGGTTTTCGATTTGGATGGTGATGGATTCATTTCTTGTGAGGAACTTGAAATTGCATTAGGTAGATTAGGGTTGTTGGAGAGGTGTTGTAATCAAAATTGTATGAAGATGATTGGCGTGTACGATGCTAATTCCGATGGGATGTTAGATTTCGAGGAGTTTAAGGAAATGATGTTTCGGGATTCATTTCATTAG |
| CDS_5730_Unigene_11635 | GCTTGCAAAGAACATAAGCTTGCAGAGTTAGGAAACATAGCTGCATGTAGAGTGTTGAACATGAGGCCGCGCGTTGCTGGGACGTATCTGGCGATGGCGAGCATATATGCAGCTAATGGTAAGTGGGGAGACTTTGCTAGGATAAGAAAACTGTTGAAAGGAATGGGGAGCAAGAAAGAGGTGGGGCGAAGTTGGATTGAGGTGAAGAATGAGATTTATAGTTTCGTTGCAGGGGATAAGCTTGGGTCCCATATTGATTGGGTTTATGAGGCTGTAGATATGCTTGGACTACATATAAAAGACATAGAATATAAC |
| CDS_30339_Unigene_49530 | TGGAGGTGGCAGCCCAGAGATTGTTCCATGCCCAAATTTAAGGCAAGATTATTGCTTGAGAAGCTGAGGAACAAGAGATTGATGTTTGTTGGTGATTCGTTGAATCGGAATCAATGGGAATCAATGATTTGTTTGGTTCAATCCATAGTCCCACCTGGCAGGAAGACCTTGAACAAGAATGGCTCTTTATCGGTTTTCAGAATTGAGGATTACAATGCAACAGTGGAGTTTTATTGGGCACCATTCTTGGTGGAATCGAATTCTGATGATCCAAATATGCATAGCATCTTGAATAGAATAATCATGCCTGGTTCAATCAAGAAGCACGGC |
| CDS_31178_Unigene_51816 | ATGGCATATGGCAGTCAACCGGTCTTTACTGCTTTGGAGGAAGAGAAAGATGAATCCTTAACTAATAAACCAAGTGGAGCCGATTGTGGTGACTGGATCCCTGGATCATACAGTTCTGTAGATGATATTATGTCTCTTCCATTTGTTTCACTGGGTCATGAGCAATTTCCTGGACGCAATATACAAGAATATGAAATGCTGCCTTCTGACCTGAGCGACAGAGGACATGATGCTGATTGTCTGATGAGCCCAGCTGCCACTTCCATAGGTTATGGCATATTAGATATTCCCATGAACTTCACCCCACCAATGTGTGAGACTAGAACAGGGTATCCAATAAAGACACATGGAGCAGATGCTCAAAACAGTAGAATTAATAGGTTCCAAATGAGTGGACAATTTCCTCCTTCACATTGCAGCACATCTATGTTGCCACTTGTGCATAATTTTGGC |
| CDS_30375_Unigene_49627 | ACCCACATCTGCGCCGCCACGAGCATGCTGACGTGGCTGCTGCTGGACATTACTTTGTGCGGAAAGTCTTCAGCCATTGGAGTCGTTAATGGTATCATCACCGGCCTCGTTTGCATCACGCCTGCAGCTGGAGTAGTAGAGTGTTGGGCAGCCATGTTGATGGGCTTAATCTCCGGCAGCCTACCCTGGTACACCCTCACCCTACTCCCTAACAAGCTCCGCCTCCTCCGCCGCGTCGACGACACCTTCGCCATCTTCCACACGCACGCGGTCGCCGGAGCTCTCGGCTCCATCCTCACCGGCCTACTCGCCGTCCCTAAACTCTGCAAACTCTTTTACAACTCGGACAACCACGTCGGCCTAGCTTACGCCATCGGGGCCGGCCGCACCTCCGCCGGGCTGCGCCAGATAGGGCTGCAATTCGTCGGACTCGTGTTCGTCGTGTTCTTGAACTGTGTTGGCACCACCCTCATTTGTCTGTTGATCAAGGTTTTTGTTCCCCTTAGGCTGAGTGAGGAAGAACTGAAAGTTGGAGATTATGTTGGTGGG |
| CDS_31087_Unigene_51711 | CAGCTGCCAAACCAGGCTGCAAAGCAACAATCAAGAACATTTCTTACTACACAGATAAACATTTTCAAAAATCCAGAGAAATATATCAATCAAAAGCAAGAAAAGAAAATGAAGGCGTGGTCTCAAAGCGTGTACGGTGGACCCGAGGTGTTGGAGCTCGTGTCGGACGTCGCCGTCCCAGAGCCGAAAGACGATCAGGTGTTGATTAAGGTGGCGGCGGCGGGGCTCAACCCAGTCGATTACAAGAGAAGAGGAGGAGTTTTTGGGAGCTATGATTCACCTCTTCCAATTGTCCCGGGTTTCGATGTGGCTGGAGTGGTGGTGAAAGTTGGGAGCAAAGTGGAGAATTTCAAGGAAGGAGACGAAGTTTATGGAGACATCATTAATCCATATGAAGGAATGAAACAATTGGGAACTTTAGCTGAGTACACAGTTAGTGAGGAGAAGTTGTTAGCCCACAAGCCCAAGAATCTTGATTTTGTACAGGCCGCTTCGCTCCCGCTCGCGATCCAAACAGCATACGGTGGCCTCGAGAGTCGAGATTTCTCGGAAGGGAAATCGATACTTGTTCTTGGAGGAGCCGGTGGGGTTGGATCCCTAGTGATTCAGCTTGCAAAACACGTGTTCGGTGCTTCAAAGATAGCTGCTACTGCCAGCACGGCAAAGTTGGATTTTCTGAAGAGTTTGGGTGCTGACGTGGCTATTGACTATACCAAGCAGAATTTTGAGGACTTGCCTGAAAAATATGATTTAGTTTACGACACTATCGGGCAACCACAGAAGGCCGTAAAAGTCTTGAAAGAAGGAGGTTCTGTAGTGGTTATTGCTGTTGGGTTTGCTCTTGACCCACCTGCCTTTTCTTATTCACTATCTGCGACTGGAAAATACTTGATGAAGTTAAACCCATATTTGGAAAGTGGCAAGGTGAAGCCGGTGATTGACCCTAAAGGCCCGTTCCCCTTCGATAAGACTAAGGAAGCTTTTGCTTATCTTGAGACAGGCCACGTTACTGGAAAGGTGGTCGTGCATCCGATTCCCTAA |
| CDS_18226_Unigene_31486 | ATGGAACCGATAGGGATCCAAGATGGATCATTTGTGCACAGATATCTGGTCGATGATCAACACGTAGCAGAAGGGAGGATACACGTGTTGAAAAATGAGGATGAGTACATCAAGGCCCTTCGACAAGGCCCGGCAGGCGGTGGGGTGGCTGCCATTGTTGATGAGCTCCCTTACGTCGAGCTCTTCCTCTCCAACACACAATGCATGTTCACCACCGTTGGACAGGACTTCACCAAGAACGGATGGGGATTCGTAAGCTCTTTCAAAATTCCCAACTCGGTCCCTGATTATCGAGATTCAAGCATCTAA |
| **Sequences of commonly upregulated transcripts in all stresses** | |
| CDS_963_Unigene_2236 | ATGCTGTCTGAGCATCCCATGAAGAAATTCTGTACTCTCTTCTTCAGGGCTGAGCTGAAGACAGAAGAAGAGCAGTTGCAGGACAGCATCCGGGTTATCAAGAATAACGAGCTTACCATTCCCAGCACAGAAGATGAAGACCCCACAAGGCCTATAAGGGCTCAGCTCAACAATACTCGCAACAGTTTCCAGCAGCAAACTCGTGCATCCCTTGAGATAGCACGCAACATGACTCGATACAGACAGTGTGTTTCTCATGACAGACAGCAGGAAATTGGATGCCACGTTTGTGTCGGGCAGGTAGAACTGTGA |
| CDS_123_Unigene_296 | ATGAAGAGGGAGCAGCACATTAAGGATTTGAATGATCAGATCACATATTTCACAACAAGGAGAGGTGAGATAGTGCAGAGAACCAACGAAATCGAGCAGCGGTACGTGGCAGTTGAAGCGGAGAACAGAATGTTGAGAATGCAGGGTGAAGAGCTGAAGAAGAGGCTGAGATTACTGGAAGAAATGCTGGTTTCTTACAACGGCGGTTATAGTTGTAGCAATAATGCTGTTGTGGAAAACAATGATGATGATTATTTTGCGATGGATGTTTTGCAAGATCCTGATCTCAAGCCATGGCTGCACCAACCATTTCAGTCTCAAACTGTT |
| CDS_21223_Unigene_34825 | ATGTCTCAAGATCACAGGCCTAATTATGCATCAGAGAGGAGGCGCGTGGAAGAATTGGGCGGATTCATCGATGATGGTTATCTCAACGGCGTTCTATCCGTTACTCGAGCTCTGGGAGACTGGGATATGAAGTTACCGCGCGGTTCTTCCTCGCCTCTCATAGCAGAGCCCGAGTTTCGGCAAATCACTCTAACCGAAGATGATGAGTTCCTCATAATTGGGTGTGATGGGATATGGGATGTGATGTCTAGTCAGCAAGCGGTGAGCCTCGTGCGTCGAGGCCTCCGACGACATGATGACCCCGAGCAGTGTGCAAAGGAGCTCGTGATGGAGGCGCTTCGGCTCAACACCTACGACAATCTTACAGTGATTATAGTCTGTTTTACGTCCATGGATCAAATGGAGCAGCCATCGACGCGGTCGAAGAGGCTGAGGTGCTGCAGCCTCTCTGCGGAGGCTTTGTGCAGCCTACGGAGTTTCTTGGACGGAAGCAGTAACCGGTGA |
| CDS_13730_Unigene_24334 | ATGACTTCGAATGCGATATTCATGACACTGAAGTTGAGAGGGTCCTCTTTCATGGTTTTCCTCTCCGTGATGCAGATGAGCAGAACAAATATTGCGAGATACGATATGTGTGAAAATACTTTGTTTCTCTTTCTCCCTTCTCCTTCAGATTTCGATTTATCCTTCTCAATACATCATGAGGATGAAGAATACCAAAATGGCCGGAGAGATGGTGGAGAGATCAACAACTGTCTCACCGGAATGCCGAGCGTTGACGCACTGAAAAATGATGGCGATTACTTTCTGGAAACCATTCAGGCCATCTAA |
| CDS_6330_Unigene_12818 | ATCGGATTTGGCGAGCAGGGAACCTTGTTTAACTGTAAAGGGATTCCTACTGCTATTAGTCTTTATGCATTCTGTTATTGTGCTCATCCAGTTTTCCCTACTTTGTACACCTCCATGAGAGACCAAAAGAAGTTCTCTAAGGTTATGTTCATATGCTTTTTGGTAGGGACAATGAGCTATGCATCAATGGCCGTGGTGGGTTACTTGATGTTCGGAGTTGCGGTTGAGTCTCAGGTAACATTGAATCTTCCGACGAATAGAGTTAGCTCGAAAGTTGCTATCTACACCACACTGGTGAACCCATTGGCTAAATATGCTTTGATGGTGACTCCAATTGTGAATGCTCTAGAGAGGCGTTTCTTGATGTTGCACCCCATGTTGATTAGGACATGTTTGGTAGTG |
| CDS_2772_Unigene_6354 | ATGTTTGAGCAAGATTCCACTGTGACTGATGTCAGTCTGGGGCAGTTTGTTGCGATTGCGATTAGGCCTTTATCTGTGATAGCAGCGCAACGGCAAAGGTCAATTTTCTCAAGAGAACGGCACCCATTTGCTATGTCACATAATCCTTCGTCCGATACTGAAGATAGGTTCCACAGAGAAAGGACTCGCAAAGAAGGAGAACTGCAAGATATGGCCTTGAGACTGAGGTTTGTAAGTCTGCGAGTAGAAGCACTTCCTCTGATCGAAAGCTTCCCCAAACCTCCACGGCTCGCAGTTCCAACAGCAATCGATGCAAGTCTTATATCTGATGCTTTTTTCCCTTCCAAGCATCGAGAGAGATAA |
| CDS_34668_Unigene_53753 | ATGGGTTCTATTGTAGCTACAATGGCAAAAGATTTAAACCTACCGAGGTTGAGGTTTGTCATATATATTTTGTTCAATCAGTTGAGAACACTGACTCAGCAGAAATTGCCAGTATTCCGCCTGCTGGTTATACTGAATTGCCCTCCTGTCCTGTTTGTCTTGAGAGATTGGACCCAGATACTAGTGGAATACAGAGCACGTTGTGTGACCATTCTTTTCATTGTTCTTGCGTCTCGAAGTGGACATACTTGTCTTGCCCGGTTTGTCAACTTTGCCAGCAGCCAAGTGAAAAGCCAGCCTGTGCTATATGTGGAACCTTAA |
| CDS_18118_Unigene_30556 | ATGGATTCGGGGCTTATAATTTGTTGGGTTTTGGTTTTGATTGAGGTGGTGTGTGTGGTGAAGGGAAATGTGGTGTTTGAAGTTCACCACAAGTATGGCGGGAAGGTGAAGGCCCCCCTTGTAGTTCTCAAGGCCCATGATTCCCACCGCCATGGCAGACTTCTTGCCAACCTTGATTTTCACCTTGGTGGTGATGGGTCTCCCACCAACGCCGCGTTATATTACACGAAAATTACAATCGGCACTCCTCCGAAAGACTATCACGTCCAGATCATCACTTGCGACCAAGAATTTTGCTCCACCGTATTTAATAGCCCGAATCCAAACTGCAAGGCTGGAATGAATTGTGAATATGCTATTACTTATGGAGATGGGAGCAAAACTGAGGGTTACTTTGTCAGAGATAACTTCAAATTTAACCAAGTTACTGGAAACAAGCAAACAGCTGCAATGGACGGATCCATAATATTCGGGTGCTCTGCTAAACAATCTGGAGAGTTGGGTTCATCCGCTCAGGCTGTCGATGGGATTGTAGGATTTGGACAATCAAATACATCCATACTTTCACAGCTTGCTACATCTGGAAAGGCGAAAAGGATTTTTTCGCATTGTTTGGATGGTAAGGAAGGAGGCGGCATCTTCGCTGTTGGAGAGGTTGTTCAGCCACAAGTAAATAAAACGCCTCTAGTTCCAAATCAGAGTTCAAGTGGGCGATAA |
| CDS_721_Unigene_1749 | ATGAGCTGCCCCATGAAGACAAGCATGTCGAGATCGATTATGCACCACATATTCAAGACCCAAATAGCTCAAATCTTCCCACACTCCATTACTCAGTTTATAAACCTCTATCTGGGGGATTATACCAAGATCAACATAGGTTATTCTAACAACTTTATAGTCATAGCCATCCGGGTCGAACCCGAACCCGAGACACTGCGTGAAAGACCCGTACGAGTTGTAAATCATGTTGGGTTTCGGAAGAAAAACCGACCTTTTAACGGAGGGATTCCACAGGATAATCGTGTTCATGTAAAAAACTCGATCGTCAGAGAGGCAGAGCAAACCGTTGCAGCTGCCCACGATCGTGAAGAAGGAATTCATGCTCTTGAATGGGAATTCAAATGTTGTGTGGTGAGTGAATGA |
| CDS_32387_Unigene_50467 | TGAGAGAAATTAAGATGGCCGGTTTTGGTGATAGCAATCCTGGCTGCAGTTGTCGGGAGCCAAGCCATCATAACCGGAACCTTTTCGATCATAAAGCAGTGCTCGGCCCTAGGGTGCTTTCCGAGGGTGAAGATAGTCCACACGTCGTCGAACTTCCATGGACAGATTTACATCCCGGAGATCAACTGGAGTTTGATGATCCTATGCCTGGCTGTCACTGTTGGCTTCAGAGATACGAGATGCATGGGCAACGCTTCAGGTACGTCGATGTTGATAACAAAATGGGGCATCTCCAATTAGGCAGACTAACTCATTAG |
| CDS_2079_Unigene_4705 | GCCCCCGAAGAGATCAAACAAGTCTTCGAGAGAAGTGAGTTGAAGAACATGGTGTGTGCACCATTAAGATTCAGACATCCATGGGAAATGCTTTGGGGAAACATAAACAAGGCCAACATTTGTGTGGTGGGCGATGCGCTGCATCCCATGACGCCCGACTTAGGGCAGGGCGGCTGTACTGCTCTCGAAGAGAGCGTGGTTTTGGCCAGACTTTTGGCGAAGGCCTTCAAACAAGACAATAAGGATGAGGAGCATGAAAGAATCCAAAAAGCAATGGATGAGTTTGCCAAGGGGAGAAGATGGAGGAGCTTTGATCTCATTGCAACCTCGTATTTTGTAGGTTTGATGCAGCAGAGTGATGGGGTGGTGATGCATTTTTTGAGGGATAAGTTGATGGCTAAGTTCCTAGCTGGACGTATGTTGAAGAAGGCTACTTTTGATTGTGGAAAGCTTGTTGTTTCTTGA |
| CDS_12160_Unigene_22167 | TGAGCCTGGCAAGCGAGGAAACCCTTGTAATTAAGAAGACAGTGGGAAAAGGACGCGCAGGCGGGGTCACGGAAGCGAGCGGCGCGGAGGTCGGCGACGGTGGCGGAGCGGAGGGAGGGGTCGTTGGAGAAGATGTTGAGGAAGAGATCGTAGAGAAGCGTGGAAAGCAGAGTAGAGGAGCAGAGCTTGTTGCCCAAGTGGAAGGAGAGGGAGCGCTCCAAGGAGGAGTGAGACAGGATTGTGGAGTAGAGATAGCTCGCCAGAGCCGGCTCCGCTTCCGCGTCAACACGAGCCTCGGATTTGATCTGGGCCCACACCCATTCCTCCTCTTCCTCTGA |
| CDS_39398_Unigene_61545 | TGGATCCTAAGGTTGTCGGAGGCAGCGTTAAGGTGGATATTAACGATCCGAGCAGGCACGAGGCGAGGTCGGGCGATGAGGAATTTATCTCGGATTTTAAGGATAAGGTGGCGAAAGATGGGTCGATTAAGTTTGTGGATAGCGTGGTGAAGGATGTTGAGGAGGCGGTTAATGTGATTCGTGAGTATAAAAGTTGTAATCTTTTCCTGGTGGGCAGAACTTCTGAGGGGGAGCTCGTTGCTGCGCTTAACAAAACGAGTGAGTGTCCGGAGCTGGGGCCTATTGGTAATTTGCTGATGTCACCGGAGATGACGACGTCGGCGTCGGTGCTG |
| CDS_38924_Unigene_60033 | GTTGATGGAGACGGTGGTACAAGACACGAAAATGTCCCGGCAAAATATTCCGAAATACCTACAACTGGAAGACGCATTAAGCGAAGAATGCAAGAAGTTGATCACCACCCTACCCAGAGAAAAGGGATGGTTCACTTCATCCTATCTCTATCAATACCAAGGTTTCTGGTATCAACCCGATCATCTTCAAGCCGCAATATCATGCCGCAAACATTTTCAAGCTCAAAATTCCGATGTCATTCTCGTCACCTTTCCCAAATGCGGCACCACATGGCTCAAAGCCATCATCTTTGCCTTACTCAACCGTAA |
| CDS_39892_Unigene_62731 | CAGAATTTTGGTGGTCACAAAGATATGGCGGTATGGAATTCGGGTGAAATCCACGCGCTTCCAGAGGACTGCATTGCCACCGTACTGTCGCTCATCAGTCCTAAGGAAGCATGCCGGCTGTCTGCGGTCGCCGCCACTTTCCGCTCCGCCTCCCTATCCGACGCCGTTTGGGGCCGCCTCTTGCCTTCCGATTATTGCGACCTCATATCCCGCGCTGTCGACGGCAGTGATTCGCTGCTCGCTAAATTTCATTCGAAGAAGGACCTCTATCTCCACCTCTGTGATCATCCTATTGTCCTTGATTGTGGC |
| CDS_39313_Unigene_61024 | TGAGGGGTGTGGAGCTTCACATTGTGGGAGGTGAGGAGAGAGAAGCAGCACATGCCGTGGAAGACGATGCGCGGGACGCGGAATTTGCGGGCGGTGTGGGATGTCCAGGAGAGGCACTTGTCGGAGATTATGCAGGTGGGCGGGGTGTGGTGGGCGGCGAGATGCTGCTCGAGCGGCTGCTGCAGTTTGTCGAGGGCGGCGTAGAAGTTTCTGATGAGGTTGGGGGAGGGGAGGGTGTCGAGATTCTCGCAGCCGGGAGGGAGGCCGACTTCTTGGCAGGGGAATGGGATTTCGATGAGGTGGATATGGAGGCCGGCGGCGCGGGCGCGGTCGATGGTTTTGGCGAAGCGCGGGGTGTTGTGGGGGGTGGTGAGGATGCTGGTGAGGACCCCATGGTGGGCTAA |
| CDS_16786_Unigene_28744 | AGCAATATGGGCTTTCAAAAGTGAGCAATGAGGCCATGTTTGTGATCGAGGCCTACTGCACTCTAAGGGACAGAGGGCCCATCCCAGCCCACCAAGTTCTCAGTGATCTTGAGGGTAGTTTTGGGTTTGTCATTTATGACCACAAGGCTGGAATCGTCTTTGCTGCCAATGTAAGTGCAATTGGTGTCTGGTTCTCAATTCTTTGTATTGAAAAACTGAGTGAAATTTTGTGGGAATTTTGTTTGCAGTGTGCTGATAAATCAGTGAGCCTATTCTGGGGCATAGCTGTTGATGGCTCTGTTATGATATCTGACTATGTAGACCTTGTGAAAGCAAGCTGTGGAAAATCTTTTGCTCCATTCCCTGCAGGGTGT |
| CDS_12189_Unigene_22188 | TTGATTCTGCCAGACAGAATTTGGCAGCAACTTTTGTCAATGCATTTGTAAATGCTGGGTTTGGTCAGGATAAGTTAGTGACAGGTGAAGCTGGAGCATCTAGTTGTACTTCGTTACCAAGCTGGCTTTTCAAACATAATGAACACGGGAGGACAAGTGCTGTTGCAAGTCTGGGCATGATTTTGCTTTGGGATATTGATTCTGGACTTGCCCAACTGTTTACATATCACGACTCCGGTGACCATCACGGCGATACTCATGTCATTGCAGGGGCATTAATAGGAATCGGGATCGCTAACTGTAAAGTCTGGCATGAGCGGAATCCCGCTTATTCTCTATTGTCAGTAAATACGAGCAATGAAAATGCTTCCATAAAA |
| CDS_14008_Unigene_24746 | GTGGACCCGCTCTTCTTCTACTCTCTCTCGATCGGGCGCGGTGGCTCACCGAGCTTGTATTTGGACGGCAGTCTGGCGGTGGCGGCGACGGTGCTGCGGACGGGCGTCGACACCGTGCATCTCTGCCATCTGTGGCTGCAGTTCCGGCTTGCTTACGTGTCGAGGGAATCGCTCGTGATTGGCTGTGGCAAGCTCGTGTGGGACGCACGCGCCGTGGCGGCGCATTATATCCGGTCGCTCAGGGGATTCTGGTTCGATGTTTTCGTGATTCTCCCAGTTCCTCAGACCGTGTTTTGGTTGGTGGTGCCGAGATTGATCAAAGATAA |
| CDS_25256_Unigene_40366 | TGGCTATGGCCATTAGTGCCTCCCTTCAGTCTGAAAGACGAGGTCCACAAATGGGAGATGCTGAGAACAGTAGCAGCCATGAAAAGAGTTGTAGTAATGACAAGTCTTCTGCTGTGAAGTATCCATCAGTTGATATTGGCCGCGAAGGTTCGTCTTGCACAATATGCTTGGATGCTCCGTTAGAGGGGGCTTGCATTCCATGTGGACACATGGTGGGATGCATGTCTTGTTTGAATGAAATCAAGGCTAAGAAAGGGGGTTGCCCTGTATGCCGCACCCACATTCATCAGCTGCTACGGATTTATGCTGCCTAG |
| CDS_22436_Unigene_36554 | TGCTAACTGAGGTCGGATTTGAGATGTTGTTCAAGCCTCAGGGTTCGTTTTTCGTGTTTGCAGAGCTCCCCGAGAGCTGCTCTCTCTCCGATGTTGAGTTTGTGGAGGAGCTAATAAAGAGAGCAGGGGTCGTAGCAGTGCCGGGGGCCGGGTTTTTCCACATGAATGGATCGAGAGATTGTTGTGATGGCTCTCGTAGTCGTCGGTATGTTAGGTTTGCTTTTTGCAAAGCCAATGAAACTCTAGCTGCTGCCTCGGCCAAGATCAAGGGACTCGTGGACAGTAAAGGGCATCTCGAGATATTCCACGTTGGAAAAACTTGA |
| CDS_28478_Unigene_44904 | TGAGAGCCCGTGGATTGCAAGAATTTGAGCAACGGCACCATGACGGGCTCCCAAGATCGATTGAAGAAAGCTTGGGAGGGCGGAAAAGGGTCGAGGATTATCGAAGAAGCGTGTGGAGTGGAGACTTTTATGCGAGAGCCAAGATTGGAAGCCACAAGGGCAGAGTGGATAAAATTCATGGCAGAGACGAGGATAGGAGCAGCATTTGGGAGTGTTGTGAGAACTTCAGAGCCGACAGATATGGCAGTGATGTTGGTGGAAGGGACATAAGCAAGAATATTGCGAGATACCCAATTGGCTGCGGTGGCATTAGACTGCCCAATGCCTAA |
| CDS_39667_Unigene_62026 | TGAAGATTCAGAGGCCATGGACTCATCTTGCTGCGGAATATGGAGGAATCACAGAATCAAGAGGCTGCCATTCCCTTCAGACAGAATCTTGCAGGTGGTCCACTCTTCGTTGCAGGAGGAGGCCACTGTTACAAAGGCTTGGTTCATACCTGTTCTCGATCAACCACTTTCCTCCAATCGCTACTACATCATCAAAGCTAAAGGAAGACGCAAAGGTGAGGCCTATACATGCTCAAGAGAGGGAGATGTCGGAATGTGCTGCTTCAGGCGGCCGAGGGCGGAGTCGAGAACGAGGCCCTTTGATCACAGAGACAGGTACCAGCAGTTCGAGATTAGGCCATTTCATGGCGGCGGTTTCTTCG |
| CDS_12972_Unigene_23286 | TGTACGCCACTTGTAGAAGGAGCAGCTCCACCGGCCAGCTCACCGACAGCGACACCAGATATTCCGACACCTCATTCGACATCCACCGCACGATCCCCACATCATTCAGCTGCTCCGCCATCCCCACTACTACTGCAAACCAAGCATATGTGTCCCACGCCGATTTCTCGCTCAAGCAGTCCTCCCAATCCAGCACTCCTGTCACCAGGAGTATCGATAATCCCACCATTGCAGCTGCAACGTTGCTGATGCCCACCCCATCTCTGCGCATCGTACGTATAACTTGCACATCTGAGATTCGTCAAGAGTTTCTACTGTCTGTCTACTCTAGTCTAGTGTTACTATGCTTACCCGAATATCCAGAGAGAGACTGCAAGAAGCATTGTGGCGATCATGATGAATTCGTTGCTTGTGAGGGGACCCATCAGCTCTAG |
| CDS_18101_Unigene_30530 | TGAATAAGCTTGGGTGGAAGCAAGTTAGTTCTCAAGCCGAAGGAGAGAGAATGATATCAGCGATGGTCTTGGCTGCAGCTCTATCACCAAGCTCTGAACCAGAGGCACTGGATACAAAAAACGAGCATCGCATGTTCATAAGAAAAATCCTTGAGTTACAGGTGGAAGATGGTGGCACGGACTATTCTTCTCTTCAAGAAGGATATATATCTGTGAGTCCTCTAGCAGCTATATCCGCTGCAGAAATAGATAGTCAAGCATTCTTCGCAGAGTGGCTGCCTCGTCTCAATGAGTGTTTCTCTTCTTCAGTCTAA |
| CDS_6781_Unigene_13621 | AAGATAAAACCTGCATGTTTCAAGAATCTGCAGCTCTTGAAGCTGCAGAGAAGTCATTCTGTGAGGTTCTTGGAGATGGTTGTTCTCCTTCGACTGATAATCAGCCAGTCCCAGATTTAGATCAGAGTATAGAAAGCCCTGAGGAAAAGTTTCTTGAAGAATCAAGTTTTTTTAATGATCATAGTAATGTAGAATCTTTTTGCCCTGATTGGTTTTATGAGCCCAAGCATATTGCTGGTGATGCTGTTTCACAGTCATCTTACAGTTGTTCAAGTGGCAATGGTACGTTAAACGACGGAGTGGTTTATTCTCCTGTTAGTATTCTCAAGATCCCAGATGTGTTGAGTGATAAAGAGTCGGCAATGCAATTTATGAAGGGAGCTGAAGAAGCAAGAAAATTTCTTCCAAGTGGCAGTAGCTCAAGGGAAAAAGAGGCAAATCTGGCTGTAAAGTTGGAAAAGAAAAGTAACGAGTCTGTTGATAAAACAGCAAGCGGGAAGAAAAATCCTTCTGAGGAGAGGAGAGCCTGGGGTTGTTAG |
| CDS_3510_Unigene_7685 | TTTCGCTCTACATAAAAAGATCTCAGCTAAACATGATCGTCTGCGTCGCCGTCGTCGGCCACCAGAATAATCTGCTCTACATACAGAGTTTTACCAAGGCAGATGATGCGCTCAAGCTTCACCACATTGTGCACTGCTCTCTCGACGTCGTTGATGAAAGAGTGAACAACCCGAAAAAGTCTGGTCCGATAATAAACGAGACGTTTCTTGGTCTGCTAAACCCAACCGAAAATTACAAAGTGTATGGTTATGTGACCAATACCAAGGTGAAGTTCATTTTGGTAACAACAGATCCTGATGTAAGAGATGCTGATATGAGAGTGTTTCGAATCCGTTTCACATTCCGGGGAAGAAAATAA |
| CDS_9187_Unigene_17758 | TGGCTGAAATCAAGTCATCTGGGAGGCCAATTGATCAATTGCTGGAGAAGGTCCTCTGCATGAATATACTTTCTTCTGATTACTTTCGAGACCTTTTGAGATTGAAGACATACCATGAAGTGATCGATGAGATTTATGTTACTGTGACACATGTGGAGCCGTGGATGACTGGCAATTGTCGGGGGCCATCAACTGCTTTCTGCCTTCTTTACAAATTCTTCACCATGAAACTTACAGTCAAGCAGATGCATGGTCTGTTGAAGCATCCAGATTCTCCTTACATAAGAGCAGTTGGATTTCTCTACTTAAGGTATGTTGCAGATCCAAAAACATTATGGGGTTGGTACGAACCATACCTTAGAGATGATGAGGTAAGACATGTTCTGTTTCTTCTTGCCTATGTGTACTTCGAATGA |
| CDS_34870_Unigene_54054 | TGTTCAAATGGTTGAGCATCTGTCCAGCAAAAGTGGCTACAGTAGATTTTCCATTAGTTCCGGTGATAGCCAAAACCTTTGTGCAACTTGGGAGAACTTCAGAGATTGCAATAAAGATTCTAGAGAATAATTTTCCAGAGGAATTCCTGGGGAAACAACAATTAGATCTGCCTTTTCAAATAGCTCATGATCAAAGCGGCCAAGAATTGTCTTCAAATGGCCATCTTTTACATTTAAAAGTTTTCCCTCCAGCGGTTCCAGTGTGTCATTCTGATCAAGGGCGAGAACTGAAGCACCTCTAG |
| CDS_1214_Unigene_2685 | CCTGCGCGCCGCGTGTCTGCACGCGCTCAAGGAGCAGCTGGCGTGCCGCGAGTCCAGAAGCGTCTCCAAGGTTCTGATGAGGCAGATGGGGACGAACATGGAGGAGCGGTGGATGAGGGCGGTGAATCTCGCCATTACCAACTGGATTTGGGAGCTGCAGGCCGGGAAGGAGGTGGTGGAGACGCCGTCGCCCTTGTTCTCCTACTCCTTCTCCATGGTGGGATTGTGGAAAGTGCAGCTCTACTGCCCCGTGATCGCCATGGCCCTCGACAAGTCGAGCGGGCCGACCCCGGACGATCGCCTCGCCTTCTCACTCACTTACCACCAGCTGGAGGGAGTGATTCAGTTGAATCATAG |
| CDS_4696_Unigene_10048 | TGTTGTCAAGGATGAGCAATACTGTGGAGAGATTAAAATCGGCCTCACTTTCACTCACCAGAGGAGCAGAGACTGCGGGGTTGAAGACGAGGACCTTGGAGGATGGAAAGAATCATCCCTCGAGTAATGCTATGCTATTCAGTTTGAACAAGGTTTTCAGATTCAAGAATCACTTCATTTTATTGCAGCTATTTGCTGTTTTCATCACTAAAACCAACCTTTATCTGCAGCTATTTGCTGTTTTCACATGTAATTTTGCATCGTCTCTGGATTTTGACAGATTTCAAGAATCTGACTGCTTTTATATATATGCATCTCATTTTGGTGCTACCATCAGCTTCATCCAACTACAGCCC |
| CDS_5853_Unigene_12054 | TTGTTTATTCCAGTATTCCCGTGGAGATGGAATCACAACCACAACTCTCCACACACAATGCAGACATCGAGAACCCAAGTTGCTGCACACTGATTCACAGGGAGTTTGGAAGGTGGGCGGGAGTTGTCCGGAATTTGAATCAAATCTTGATGCGAATAGTGCCGCGAGCCAAGTGGATTCACGAGCAAAAAGTGAAGCATCATCACGCACTACAGCTTCTCACATGCTTGTGCGAGCATTTGGAGTCGTTGAGCGACATAGATGCTGCACATATTTACTCCCACGCCGTGGTGAAGGCGGCTGAGTTGGGAATTCACGAAGTTATAGAAGCCATCGTACAGAAATTCCCCGTTGCCGTTTATTCTGCCGTGCCTCCTAATTACCAATACTTCTTCCACATAGCCGTCGCAAACCGCCGCGAAAAAGTTTTCAATCTGATTTATCAGATGAGTGACCACAAATACCAATTCTCAAACATAAAAGACCTCTCCGGCAACACTATCCTCCACTTGGTCGCGAGTTTAGCGCCTCCGCACAAACTAAATCTAGTCTCC |
| CDS_1678_Unigene_3880 | TGCACACCAGGATCATTGCTCTCGTAGCTGCCACCGAGCATGACGGAGTTGTTGATAGCTTGAAAGTTGCTGTTGACGGCGTAGGCGGCGGCGTCATCTTCCTGCTCCGGCGGAGCGCCGGCGGCCTTCTCGTTGTCCACGTCACCGCGCATGGTGGCTCCGATGTTGCTTCCGGCGAGGGTGATGATCCTCACTCCTTGCTCGTCGTCGGAGGCCGAGCCCTCCGGTTTGTGGGGGCGGTGGAGGCTGCCGAGGCCGGTGGTGACGGCTGCGATCATGTCTCGGAGGTCTTGATCGAGATTCTTGTGGTTGTCGGCGCCGCCGTCGCTTGCGGTGGCGTTGTTTAG |
| **Sequences of commonly downregulated transcripts in all stresses** | |
| CDS_27156_Unigene_43001 | TGGATTTTTGGAACAGAGCTCGCAGCTTCGCCGATGAAGCTGCTAAGAAATCGCAGGAGCTCAGGCAGACCATCGCCACTACCAACCTATCCGGTGTCGTTTCCGAGGCATCCAAGCGCTCCAAAGAGCTCGCCCTCGAGGCCTCCAAGCGCTCAAAGGAGCTCGCCGTCGAGGCTCTCAAGCGCGCTGATCAGATCACTGCTCAGATTCCCCCTGCCGCCGCCGCCATTAATAGTTTAGTCGATTCCGCCGCTCCCAAGCCCGCCATTGACGACGCTGATCTCGACAAGTATGGGATTACTGATGAACTCAGGGAGTTTGTCAAAGAAATAACCATTAATACATTCCAGGATTTTCCGCTCGAAGATGACTCAGAGATGTCTGATATTCCTACGGTTTCAAATGTCCAACAGGATCTCACAGAGTGGCAAGTAAAGCATGCAAAGCTGGTTCTCTTAAATGTGAAGGAAGTTTCGAAGCTTAGGTATCAGCTATGTCCAAGAGTGATGAAGGAGAGGAAATTCTGGAGAATCTACTTCATTCTCGTTAACAGTCATGTTGCACCATATGAACAGCGCTACATGGATGATGCAAAGATTAGAAGTGCTGAAAAGCTAAAAGATACTGACGTGAAGGAAGTTTCATCATGTGAAACATCTTCTAAAATAATGGATGAAGTTGTTAAACCTAAAAGTGATGATACAAAAAAGCCTTTTGAACAAGATCTGGATGCATTTCTTCTTGGTGATCTTGAGGATGGTGATGATGGTACAGATGATGGTGACAATGGCTTTGATGATGATTTTGACAAGATATAG |
| CDS_20547_Unigene_33835 | TGGAAACGAAAGTCATTAGTGGAACACAGTTCACTAGCCTCCCCAGTAATTATATCCGTCCCGAATCTGAGAGGCCAAAGTTATCTGAAGTTGCTGATTGTGAAGATGTTCCCGTCATTGATTTGGGCTGCGAAGATCGTAGCCTTATAGTCAAACAGATCCGTGATGCTTGTCGAGAATACGGGTTTTTCCAGGTGATCAATCATGCAGTGCCAAAAGACATAGTGGACAATATGGTGGAGGTGGCTCATGAATTCTTTAGTCTATCTGTGGAGGAGAAGATGAAATTATACTCTGATGACCCTTCCAAAACTATGCGACTCTCTACGAGTTTCAACGTTAAAAAGGAGACCGTTCACAACTGGAGAGATTATCTCAGGCTTCATTGTTACCCCTTAGAGAAATATGTGCCTGAATGGCCATCCAATCCCTGCTCTTTCAAGGATATCGTAAGCACATACTGCAAAGAAGTTCGGGCTCTGGGATTCAGATTGCAAGAGATCATATCGGAGAGCTTAGGTTTACACGGAGACTGCCTCAAGAATGTATTGGGAGAACAAGGGCAACATATGGCCATCAACTATTATCCTGCATGCCCACAACCAGATCTGACTTATGGATTACCCGCTCATACAGATCCGAATGCCCTCACCATTCTCCTTCAAGATTTACAAGTATCGGGTCTTCAGGTTCTTAAGAATGGGAAATGGTTAGCAATAAAACCCCAGCCTGATGCTTTTGTCATCAATATTGGTGATCAAATCCAGGCATTCAGTAATGGGAAGTATAGAAGCGTGTGGCATCGAGCTGTTGTAAATTCAAACAAAGCTAGGCTCTCGGTTGCTTCATTCCTCTGCCCATGCGATGCAGCAAACATTAGCGCTCCAAATGAACTCACAATTGGTGATGATCAAGCGATATACAGAGATTTTACATATGCCGAGTACTACAAAAAGTTCTGGAGCCGGAACCTGGACCAGGAGCACTGCCTGGAACTATTCAAGAATTAG |
| CDS_1507_Unigene_3270 | TGGCTTCCGACGGTTTTCAGCCTCTCGACGAGAAGTTACTGGTGGCGTACGTGAAGGCAACTCCATCTCTCGCCGCCAAGCTTGGAACTCATTTCGATAAGCTCGAAATCAAGGAAGTTGGCGACGGAAATCTCAACTTTGTTTACATTGTCATTGGATCATCAGGATCTCTAGTCATCAAGCAGGCTCTTCCATATATTCGTTGCATAGGAGAGTCGTGGCCAATGTCAAAAGAACGTGCCTATTTTGAAGCATCAGTTTTGCAAGAACATGGTCGCTTGTGTCCGGATCACGTTCCTGAGGTTTACCACTTTGACAGGACGATGTGTCTGATTGGTATGAGGTACATTGAACCGCCTCATATCATATTAAGAAAAGGATTGATTGCTGGCGTTGAGTATCCATTGCTTGCAGACCATATATCAGAATATATGGCACGGACACTGTTCTATACCTCACTACTGTATCTGACCACTCTTGATCACAAAAGTGCTGTTGCTAAATTTTGTGGAAATGTGGAGTTATGCCGACTTACTGAACAGGTAGTGTTTTCAGACCCGTACAAAGTGTCTGAATATAACCGTTGGACAACTCCTTATCTTGATGGTGATGCTAAGGCAGTTCGGGAGGATAACATTTTGAAACTTGAAGTTGCTGGGCTGAAATCTAAATTCTGTGAGAGAGCCCAAGCTCTTTTACATGGTGATCTCCACACAGGGTCAGTCATGGTCACTCCTGATTCAACTCAAGTGATAGATCCTGAATTTGGTTTCTATGGGCCAATGGGATTTGATATTGGAGCTTTTATTGGAAACCTGATTTTAGCCTATTTTTCACAAGATGGACATGCGGAAAAGGGTGCAGATCGTAATCTATATAAGAAGTGGATCTTGAAGACAATATTAGATACTTGGAACCTCTTCTACACAAAGTTCACTGCTCTTTGGGATAAACACAAGGATGGTTCAGGTGAGGCATATCTTCCAGCAATTTATAACAATCCAGAACTCCAGTTGCTCGTAAAGAAGAGATACATGGAAGATCTTTTCCATGATTCTCTTGGATTTGGTGCTGCCAAAATGATAAGGAGAATTGTTGGGGTGGCTCATGTTGAAGATCTGGAATCAATTAAAGAGGCGAGCAAGCGTGCTGAATGTGAGCGTAGAGCTCTGAACTTTGCTAAGACCCTTCTGAAGGAAAGGAAAAACTTCCACAACATAACCGAAGTTGTTTCTGCAATTGAGGATCTCGACAAATAA |
| CDS_37441_Unigene_57434 | CTCAAATTCATCCCTCCTCAGTCCTCATCTCAACAATTCTCCATTTCTCTTCTCAAATTCCATCAAATCTCATCTCTAAAATGCCTTCAATCCCAGAGGAACCCCTCTTGGCCCCTAACCCCGACCGCTTCTGCATGTTTCCGATCCAATACCCTCAAGTCTGGGAGATGTACAAGAAGGCCGAGGCCTCCTTTTGGACGGCGGAGGAGGTAGATCTCTCGCAGGACCTCCGCCACTGGGAATCCCTCACCTCCGACGAGAAGCATTTCATCAAACACGTTCTGGCCTTCTTCGCTGCCTCCGACGGCATCGTTTTGGAGAATCTCGCCGGCCGGTTCATGAAGGAGGTCCAGATCTCCGAGGCGCGCGCCTTCTATGGCTTCCAGATCGCCATCGAGAACATCCATTCCGAGATGTACTCTCTCCTGCTCGAGACCTACATCAAGGACTCAGAGGAGAAGAGCCACCTATTCCGCGCGATCGAGACCGTCCCCTGCGTCGAGAAGAAGGCCAATTGGGCGCTGAGCTGGATCGACGGATCTGAGACGTTCGCGGAGAGGCTGATAGCCTTTGCGTGTGTGGAGGGGATTTTCTTCTCCGGCAGCTTCTGCGCGATATTCTGGCTGAAGAAGCGCGGCCTCATGCCCGGGCTAACGTTTTCGAATGAGCTGATCTCACGAGACGAAGGTCTGCATTGCGATTTCGCTTGTTTGCTGTACAGCTTGTTGAAGATGAAGCTTAGCGAGGAGAAAGTGAGGAAGATTGTTGGTGATGCTGTTGCTATCGAGCGGGAGTTCATATGCGATGCTCTGCCTTGTGCGTTGGTGGGGATGAATGAAACGTTGATGTGTCAGTACATTGAGTTTGTGGCGGATAGATTGCTGAATTCATTAGGGTATGGGAAGATGTTCAATGTGCAGAACCCTTTTGATTGGATGGAGTTGATCAGTTTGCAGGGGAAGACAAACTTCTTCGAGAAAAGGGTTGGTGAGTATCAGAAGGCGTCTGTGATGTCGAGCTTGAACGCCAATGGGGGCGATTCTCATGTGTTCAAGTTGGATGAAGATTTCTGA |
| CDS_24654_Unigene_39576 | TGCCTTATCGTAACATAATCACCATCACCTGCCTATTTCTACTGCTAAATGCTGTTGTTGGTGCACCACAATTTCCATGTTTCTTCATATTCGGGGATTCATTGGTGGACAACGGCAACAACAACCATCGCGAGACCACCGCCAAAGTTAACTACCTGCCTTATGGCGTTGATTTTCCGACCGGCCCCACCGGCAGATTCTCCAACGGCCGAAATATTGCAGATGCCATCGCTGAGAAGTTGGGGTTCAATAAATCAGTTCCTCCATTCGCCACTGCAACGGATGAGGACCTCCTCCTAGGCGTCAATTATGCCTCCGGTGGATCGGGGATTCTCGATGATTCCGGATCCATCTTCGGTGACATTTCAACGTTCAACGAGCAATTAGCGAATCACGAGGTCGCGATTTCGAGACTGGCGGCAAAATTCGGAACCAAAACAGAAGCACACAAACACCTCAAGTCGTGCCTGTACTACGTCGGAATCGGAAGCAACGATTACCTCGGCAACTACCTGCCCAAATACTATGCCGCCGCCGCTAAATACACCCCCGAGCAGTTCGCCGCCGTCGCCATCGAACAATACTCCAAGCAGCTGCAAAGGCTCTACAACGCCGGCGCCAGGAAGATCGCCGTCGTCGCACTCGGCCAGATCGGCTGCATCCCGCAGCAGATCCTCATGTACGGCTCCGGCTCCGCGCCCTGCGTCGAGACCACAAACGACGCCGTTCTGATGTTCAACGGCGAGCTCCAGCGCCTCGTCAAAGACCTAAACGTCCAATTCCCAGACGCTCAGTTTCACTACACGGCCGATATCTCCGACGAAGCACCGTCGTACGGTAATATAAAAGTTGTGACCGAGCCGTGCTGCGAGGTGTCGGAGGAGAGACTGGGGCACTGCGTGGAGGGAGGGGAGATTTGCAGAAACAGAGATGAATACTATTTCTGGGATTGGTATCATCCATCTGAGGCCGCCGCCTTGTTGTCGGGAGCAAGTCTGTACAAATCCATGTCTCCATTGTTTACTGATCTTGTCGATATTTTGTGA |
| **Transcripts showing Antagoanistic behaviour** | |
| CDS_7254_Unigene_14466 | TGAAGAGTGGTAGCCAGCATTGTGGGGCCTCTGTAAGTTACCAAATTAGCCTTCTTCACAATCCAAACATTCCCAACTTGAGCAAAGACAGGATTAGCCGACACGAATCTGGCGATCTCGCGGTGGTCTTGGAGGGGGGCGGCGGCGTCGAGGTGGATGAGGTAGTGGTTGGCGGGGTGGTAAAGGGCGAGGATGAGGCGCTTGAGCTTCATGGTGTCGCCGGAGGAGGCGGAGATGAGGTAGGCGAAGGTGGCGTTGTAGGGGTTGCTTCCAACGATGAAGGTGGTGGGTGGTTTGAGGGTGGTGATGGGATTAGTGGATTTGGAGGGGGTAAAATTGTAAAGGAGGGAGAGGAGGAGGGAGGTGGTTAG |
| CDS_34490_Unigene_53496 | ACTTGTTTTGGCTGCTTCATGGTATATTTCATCAATTCTTCCCTCATTCACATTTTCATTCATCTCGCATACACCTAATTATGTCACACATTTGTGAATTAATGTTTGGATTAGGCAACAGAGGACCGGGAAGAACACCACTAGATTGGACAACAAGACTGAAAATTGCAGCAGGGGCAGCTCGAGGAGTAGCCTTCATCCACTCATCACTTAAACTCGCCCATGGCAACATCAAGTCCACAAACGTACTCATCGACAAGGATGGCAATGCAAAGGTCTCAGATTTTGGCCTCTCTCTTCTTGCATCGCCTTCCTCAATCCCAAAATCTAACGGCTACCGCGCCCCTGAGGGATCATGTAAACCAACACAAAAGAGCGATGTCTACTCCTTTGGCGTGCTCTTGCTGGAGCTGCTCACGGGGAAATGCCCGGAAAGCAGCGGGCCGGGTGTGATGGTGGTGGATTTGGCGAAGTGGGTGCAGTCGGTGGTGAGGGAGGAGTGGACGGCGGAGGTGTTCGATCTGGAGCTGATGAGGTATAAGAATATTGAGGAGGAAATGGTGGGGTTGTTGCAGATTGGGATGGCCTGCGCTCAAGCTTCACCAGATCAGAGACCCAACATGAACTTGGTGGTCAACATGATAGATGATTTGCGGCCATCATCTCAACAACCTCTTCTTGCTCAAGAGCTTTGA |
| CDS_38353_Unigene_58711 | TGGCGGCTGTAGCGATAATGGATCTGATGAGAAAAAACCCGAGCTTTGGAATGGGAGGGCAGACGTTTAATTCAAGAGGGCTATTCGCCGGAAAGCTAGACGCCTCTGCCGCTTCCTACGCCTTCACCCTTCCTTTCGCCTTCGGTGCATTACTTGGTGGTGGAGCGGTGCAATATTCCTATTGCGATGCTGGCGCCACATTGGGCGAAGATTACCTTTCTAGTATTAGGTCTGCATCTGGAAAAATCTTCCAAAGCGATGCACTTAAATACAGTACCAAGCAGTATAATATTCAGTTGAAGCCGATACTCTCAGCTTTTCATTGGAAAACTCTTGCGCTGACATCACTGAGGTCGTTTTCGTTGTTCTATCTTCCTCTTCTAGAACCCCACTTATCCATGGAAGATGAGGAGGATGATTTTCTAAGCGATGATACAGCAGAAAGTCGTATAGACTTGACTATTCCTTTCAAGAGATCAGTGAAACAGATAGTGATGGAGACAAGTATAGTAACCACACGGCGCCTTCTTGAAAACCTTTCAGTTTATTACATCTCGCAACGCATGGCGTGGAAACTTGTCAAGGACATTCCGAAATCAGCCCTTCGGAAGGCTGGAAGAGGAATGCCGACTTTCAAATACATATACTGTGTGAGCAGAACCACTTTTAGAGGACATTTTCTTGGAGTTTTAGCTTCGTGGCTGGTCCACGTTGGCATCGATGTCTATCAGTTCCTTTCATCTGGATATAAAGCCAGACGAGGCGCAGACACAGTTGACACAAAGAAACGAGTTATAATTCTTGGACGGCAGGTTTATGGTGCCACTGTGAGGGGTTGTGCATCGCTCCTTTTTGCTTCGGTGGGGGCCGGAATTGGAGCAACTTTGACTCGTCCTTCAGCTGGACAATGGATTGGGAGTGGCATTGGGGACTTGGCTGGACCTATTGTTGTGGCCTTTTGTTTTGAAAGAGTTCGTGCTGATCTCTGA |
| CDS_969_Unigene_2245 | TGGCATCGATGCAAACTAGTCTGGCGACGGCTGTGTGTAACAACAGAAATTGCACTACTTCATCGAAATTTCTCAACTCCTCCTTTCTCCATGGAACTGACGTTAATGGACAGTTTATGGGCATACGACGAAAGGAAGCCTGTCATGCTTCCTTTTCAGGGCCCAAAGCTGCACTTACCTTCGACCCCTCGACTTCTAACCAAGATAAAGTTAAGATGCGAAAGCCGACTGCTGATCCAAACGCCCCCGATTTCCTCCCGCTTCCATCCTTCGAGGAATGCTTCCCTAAAAGCTCCAAAGAATACATGGAAGTCATTCACGAGCCATCTGGGCATGTGATGCATGTTCCATTTAGGCGCGTCCATCTTTCAGGAGACGAGCCACATTTCGACACTTACGATACCAGTGGCCCTCAAAACATCAGCCCGCGCATTGGACTTCCGAAGCTGCGGAGGGAGTGGATTGACCGAAGAGAAAAGTTGGGTGGACCAAGGTACACTCAGATGTTCTACGCGAAGCAGGGAATCATCACCGAAGAGATGGCGTTCTGCGCTGCTCGCGAGAAACTCGACCCGGAATTTGTTCGATCCGAAGTTGCGCGTGGCCGCGCAATCATCCCGTCGAACAAGAAGCACCTCGAGCTCGAGCCAATGGTGGTGGGACGCAATTTCTTGGTGAAAGTAAATGCCAACATCGGCAACTCCGCGGTCGTAAGCTCAATAGAGGAGGAAGTCCACAAACTGCAGTGGGCAACAATGTGGGGCGCTGACACTATCATGGATCTTTCGACTGGACGCCACATCCACGAAACCCGCGAGTGGATCCTACGCAACTCCGCTGTACCCGTTGGCACCGTGCCCATCTACCAAGCCCTCGAGAAGGTGGACGGGATCGCCGAGAATCTCACCTGGGAAGTCTTCCGAGAAACACTGATAGAGCAAGCTGAGCAGGGTGTTGATTATTTCACGATCCACGCGGGAGTCCTCCTTCGTTACATCCCTCTAACCGCTAAGAGGATGACAGGAATCGTCTCTCGCGGTGGATCCATCCACGCAAAGTGGTGCCTAGCTTACCACAAGGAAAACTTTGCATATGAGCACTGGGATGAGATACTGGACATTTGTAATCAATACGACATAGCCTTGTCGATTGGCGATGGATTGAGGCCCGGATCTATATACGATGCTAACGACACTGCTCAGTTCGCGGAGCTCTTGACGCAGGGAGAGTTAACGCGGCGAGCGTGGGAAAAGGACGTGCAAGTGATGAATGAAGGGCCTGGACATATCCCGATGCACAAGATCCCCGAAAACATGCAGAAGCAGCTCGAGTGGTGCAACGAAGCGCCTTTCTACACGCTCGGGCCTCTGACAACCGACATCGCGCCTGGCTACGACCACATCACTTCCGCCATTGGAGCCGCCAACATCGGAGCTCTCGGAACCGCACTTCTCTGTTACGTCACTCCCAAAGAGCATCTCGGCCTGCCTAATCGCGATGACGTGAAGGCGGGTGTTATAGCCTACAAAATAGCCGCGCATGCTGCGGATTTGGCGAAAAAGCACCCTCACGCGCAGGCTTGGGACGATGCGTTGAGTAAGGCGAGGTTTGAATTTCGGTGGATGGATCAGTTTGCGCTGTCGTTGGACCCCATGACGGCGATGTCGTTCCATGATGAGACTTTGCCGGCTGAGGGGGCGAAAGTGGCGCATTTCTGCTCCATGTGCGGGCCTAAGTTTTGCTCGATGAAGATAACCGAGGATGTGAGGAAATACGCGGAGGAGCATGGGTATGGGAATGCCGAGGAGGCCGTGAAGGAAGGTATGGATGCCATGAGCGCACGATTCCTGGCTGCCAAGAAGACTGTGAGTGGAGAGCAGCATGGTGAGGTTGGAGGGGAGATCTACTTGCCTGAGGAGTATGTGAAATCTAAGGCAGTTTGA |
| CDS_10439_Unigene_19668 | CAATTCGTTTCATTTATTGCAATCCGCACACGCAAAAAGCACAGCAAATATCATTTTCCAGGAATTCTTTTTCATTTTTTCGATTTAATTTAATTATCTTAATTATGGTTCAATTGAAGAAGAAGCTTTCCGGCAAAATGAGACTGAAGTATACTCCGCCGTACGCCGGCGCCGGCGACATGCCGGAACCGGATATCCACGTGGTTACCTCCGCCGGCCAGCGGATTCCGGCTCACTCGAAGATCTTGGCATCAGCATCGCCGGTGCTGGAGAGCATTATAGAGAGGCCGCAGAAGCACCGGAGCACCGAACGGAGGATCACGATCTCCGGCGTACCGTACGACGCCGTTCACGTCTTCCTCCAGTTTATGTATTTCTCGAAGTGTAGCGAGGAGCAGCTGGAGCAGTATGGAATTCACCTACTAGCTTTGTCGCATGTATACTCGGTACCACATCTGAAGCAGATAGTTACAAAAGACTTAGCCAAGAGATTGAGCGTTGGAAACGTAGTTGATGTGCTTCAGCTAGCAAGATTGTGCGACGCGCCGGATCTTTACCTCAAGTGTATGAAATTAGTACACAATAAGTTCAAATCCGTTGAGGAGACTGAGGCGTGGCAGTTCCTGCAAGATCACGATCCCCATCTCGAGCTCCAGATTCTAGAGTTCATCGATGAAGCTGAATTGAGGAAGAAGAAGACGAGGAGGCATAGGCAGGATCAGAGTTTGTATATGCAATTGAGTGAAGCAATGGACTGTCTCGAGCATATATGTTCGGAGGGATGCACGAGCGTTGGCCCGTACGACATGGATCCGGACAGGCATAAGGGCCCGTGCAGCAAGTTTTCGACGTGCCAAGGAATCCAGCTTCTGATCAAGCATTTTGGTGCTTGTAAGAAGAGGGTTAACGGAGGGTGTTCGCGTTGTACGCGGATGTGGCAGCTGTTTAGGCTGCATTCTTCGATTTGTGATCAGCCCGATGAATGCAGAGTGCCCCTTTGCAGGCAATTCAAGTTGAAGGCACAACAAGATCGAAGAGGGCACGATCCACGGTGGAGATTGCTCGTGAGGAAAGTCATCTTGGCCAAAGCAATGTCGTCTCTATCACTACCGAAGAGGAAGAGGGAAGATGAGCCGCGAATGGCGTTGCACGGGAAAGGGGGGAGAAGCTTCACACTGTGA |
| CDS_15938_Unigene_27513 | AGAAATATGCCGACCTAGAGAAGGAGAATGAAACCATGCACTTTCAAGTGGAAAAACTAAAGGTTTCATTGGGCGAGGAGAAGAAAGAATGGATAGTCTCTCAGCTGCTAAGTGAGACTAGAATGGCTGGACTTGAAAATGAGATCAATCGCCTGCAAGAAGAAAATAAATCGAAGAAGAAAGAATCCAAACAGGAAATTGAAAAGGCTCTAAAAGCACAGTTTGAAATATCCATATTGCACAAATTCATAAAAGATATGGAAAACAAGAACTGTGCTCTCATCATCGAGTGTCATAAACATGTCGAGGCGTCTAATTTGGCTGAGAAAGTGATATCAGAGCTTGAGAATGGAAGTCTTGAGCAGCAGGTGGAAACTGAACTCTTATTGGATGAAATCAGAAGACTGAGATTGAGCATATACCAGATTTTCGTGTCGCTGGAGGGCAGTCCAGATTCAGCACCTGAAAACAAGGTTGAAAACGAGCGAACTTTGGTGAATCATATTCTGGAATGTGTCGAAGATATGAAATGTGTTATCTCCAAGAACAAGGATGAAAGGCTGCAGCTTGTTGTAGAGAATTCAGTTCTGGAAGCACTCCTTGAGCAATTGCAGTTTAAGGGCATGGAAATTGAGTTACAGAAGCTAGACTTGGATCAGGAGGTGAAAATCGCCGCCGAGAAACTTGCCACTGTGAACAGTGAAAAAGAAGAGCTCTTAGAGATAAACAGGAAATTAAAATCGGATGCAAGCAAGGGCTATGAGGCTGCTGCCATTCTTCAGGCTGAAATGGGGAGACTTGGTGCTGAACAAGGTGATCTGCAAACAGCTTATAATGCATTACAGGATGCATACTCTAGAGCAAAGCAGGAAAATGAATGTTTGTTGAAGAAGTTCTATGGTCTAATCGAGGAGAAAAGTCGGGTGGATCAACTAAATGATGATATTCTCTTGGATTTATTGGCAGCCATCAATCAATCTAAAGTTCTAAAGAGCTTTGCGGTTGAGAAAATCATGGAATTAAGATCGCTTCTCGAAGATCTGAACAGACAACGTGATGTGAACAGTTGCCTCGAAAGAGAAATGAGTCAGTTGGGGGCCAAGCTGGAACTACAAAAGACTGAAAATATAGTCGTTAAAGATGTTGTGTGTAGCATGGAGAGAGAGATGCAGGAGATCAAAGAATGTAATGATCAAATGAAGCAGGAAATAGCAAATAGCACAGAGATCTTAACGGAAACAAAAGCAAACCTTTTTGATACAGCAATGCAGCTTGAAGCTACCGAAAAGCTGAACTCTACGTTGTTCAGAACAGTAGCGGAGCTGAGGATTGACATTCAGGAGTCACAGCATGTAAGAGAAGATCTGGAGAAGAACATGGTCCAGTTGTCTGAAACTAATTCTTCGCAGATGAAGGAAATTCAGAGCCTTCACAAAGTCAACAAAAATCTAGAGTCTGAACTTGTCTTACTTCATCGAGAAATTGAAGAAAATATAGTGAGAGAGCAGACTCTGAGTATTGAGCTCCAAGGCATGAACAACGAATTTGAGCTATGGGAGGCCGAAGCAGAAACCTGTTGTTTCGATCTTCAGGTCTCTTCTATCCAAGAAGTTCTGTTGAAAAATGTGGTACAGGAGCTCACTGGGGTGTGTAAGACTCTCGAGCACAAACATGTTGCAAAAACATTGGAGATTGAGCAAATGAAAGGAAAGTTTAGTCTCATGGAGAATGAAATTGACAGATTGAAATCCCAAGTTTTCGCATATGCTCCAGTTATTGCTTCCCTAAGAGATGATATAACACTCATAGAGCATAATGTGCTGCTTCATTCAAAGCTGAAAGCTACCCCCAGTCAAGGGAAAGAGTTTCTGGAATTTGTAGTACATCCTAATGAAGCCACATCTCAGACACTTTGGGAGGATCAATCCTTTCGTAGTTTACAGGATTTACAAAAGAGAGTCGAGGCTGTTGGAAAGTTGATGGAAGAAACAAATGGACCTGTATTACAAAGAAGATCGAATTCGAAGAGCAAACAAGCAAATGATTCTCCCAGGTTGCAGATGAAAAGCAAAGCTTATGAAGTCCGAAATATGATGTTGATGAAAGATATTCCTCTTGACCAAGCCTCTGATAGCTTCATGCAAAACAAAAGCAATGCTAGCTCTGATGATCCAATGCTTGAGCTTTGGGAAACTGTAGAAGATGGAAACAGAGATCAAACAACCAGTGAATCTCTTATGATGCCGTACAATTTTAAGGGAAGGGACAGAGCTTATGATCAAGCTGAATACATCAAGGGAAAGTCTTATCTCCCATCCACTGATTCAGATATGGAGAAAGAGTTGGGTGAGGATAAGTTGGAGTTGTCGTCTAGATTCCCCGAGCCTAATCCAGAATTGGACGACGAAACCATCTTGGAGAGACTTGCATCTGATGCTGAGAAGCTAGAAAGTCTTCAGACAATACTGTACAACTTCAGAAGGAAAATAGAGACAAGCAAGAAGAGTAGAAAGGCTAAGAAGATCGATTTTGATGCAGTTAAGGGACAGTTGCAGGAAGCTGAGGATACTCTTGCACATTTGTTTGATTTGAATGTTCAGCTAGTGAAGAACATAGAAGAGTGCCCTCCAGATGAAATGGTGTCGCTGAGAATGAAGGAGACTGTAAAAACATGGAGGATTAAGGTGATGGAACAGGCACAAAAAGGGTCTGAAAGGTTGGACATGCTGCACCTAGCCGTTCAAAGGATTCAGTTAGTATTGTCGAAACTGGAAGACGAAGGCAAGATTAGCAAAGGGAATAATAAATTCTTGAGGAGCAGAAATGTCATTTTGAGGGACTTCATTTACAATGGAAAGAGAAATAGTGGAAGACGGAAGAAGAGTCCAAACTGTGGTTGTTTCAAACAATCAACTAGTGGAATTGAAGCAGGTAGAGTTCATTACTTC |
| CDS_29256_Unigene_45959 | TGGAGGCTTCAGCATTGTGGATTTCAAACTCAGCTTCTATATCCAGCGTTGCTTTGCTGAGCAGTAACAGGAGATGGAAATGGCAGTTCGCATTCAAGAAAAGCTGGCCCTCCATCGACTCTCGCAGATGCTTTCTTGTGAAGAATGTGGCTAGCAAGAAAACAACCAAGACACTTGAGGACCCTTCTCTTGAAGAAGAACAAGGAGTTGCATTAGATGCTCTAAAGCCAGATTCTGCATCTGTGGCGTCAAGCATAAAGTATCATGCCGAGTTCACGCCATCATTCTCTCCTGAGCTTTTTGACCTTCCAAAGGCATACTATGCCACCGCTGAGAGTGTTCGCGATATGCTCATTATAAACTGGAACGCGACCTATGATTACTACGAGAAGATGAATGTAAAGCAGGCATATTATCTGTCCATGGAGTTTCTCCAGGGTAGAGCTTTACTGAATGCAGTAGGTAATTTGGAGCTCACGGGTGCTTATGCAGAGGCATTGAAAAAACTCGGCCACAGTTTAGAAGATGTAGCAAGACAGGAACCGGATGCAGCATTAGGTAATGGAGGATTGGGGAGGCTCGCATCTTGCTTCTTAGACTCCATTGCGACGCTTAATTACCCAGCTTGGGGTTACGGGCTCAGATACAGATATGGACTATTTAAGCAGCTCATCACCAAAGATGGTCAAGAGGAAGTTGCTGAAGATTGGCTTGAGATGGGGAATCCATGGGAAATAGTGAGAAATGATATCTCTTATCCAGTTAAATTCTACGGAGAAGTCATAGAAGGCCCTGAAGGAAAGAGAGAATGGATTGGAGGAGAAGACGTCGTTGCTGTCGCCTATGATGTCCCAATACCAGGATATAAAACCAAAACTACCATCAACCTCAGGCTGTGGTCTACGAAAGTTGCTGCAGAAGTTTTTGATTTACAGGCATTTAATTCTGGAGATCATCCAAGAGCATATGAAGCCTTGAAAAGGGCTGAAAAGATTTGCTATGTTTTATACCCGGGAGATGAATCATATGAGGGAAAGACGCTTCGATTGAAGCAACAGTATACTCTCTGCTCAGCTTCTCTCCAGGACATTATATCAAGATTCGAGAAGAGATCAGGGGCGTCGATAGATTGGGAGAAGTTTCCTGAAAAGGTTGCTGTACAAATGAATGATACTCATCCTACTCTATGTATACCTGAGCTGATAAGAATATTGGTAGATGTCAAAGGACTTAGTTGGGATGAAGCCTGGGGAATTACAAAGAGAACTGTGGCTTATACTAACCACACTGTTCTACCTGAGGCTCTTGAGAAATGGAGTTTGAGTCTTCTTGAAGAACTTCTTCCACGGCATGTTGAAATCATTAGAATGATTGATGAGGAGCTAATAAATACTATAATAGAAGAATATGGTGCTGAGGATCTCGAGTTGCTGAAACAAAAGCTGAAGGAAATGAAGATCTTGGATAATGTCGAACTTCCTTCTTCTGTCATCGATTTACTCGTTGAAACACAGGAAAACCTCGCCAAAAACTTGGTCCAAGAAGAAGAAGAGGAGGAGGAGGTGTGTGTTGTGTTGGAGTTGTGTTGTATTGCAGTGAGCAAAGTGGAGTGTGGTGGCCACACGCCT |
| CDS_13712_Unigene_24313 | TGATGCCTCAAATGTATTGTGTACAATCGAACCAGTCACCTTTGCCTAGTCCTGGCATGGCTATCAACTCGCAGTCTTTCACTTACTTAAATCCATCAGACCGTCAACCCACAAATTCTCCAAATTTTCAGAACCTTGTTGATCAAAGGATTAGTTGCACCATGGATCAGATTGATAATAAACAAGGCCAGAAGCTGGAAAATTTGGAGGATAGGGGGCGTGTTTCTTTCACCAATGATCATCAGAGTCCTAACAGTGGCTTTTATAGTGGCTATACAAGTCACCATCAAAGTGTTGGTTCTGGTGACAATGGCAAGATAAACTCAATCTCAGTCGTCAAGACCACATCAGAAGCTGCTAGCGAAGAGGGGCTTCATGTTCAACAGAGTGCCTCTCATCGAACCATGCAAAGAGAAGCTGCTCTAACAAAATTCAGACTGAAGAGGAAGGAAAGATGCTTTGAGAAGAAGGTGCGGTATGAAAGCAGAAGGAAGCTGGCTGAGCAGCGTCCTCGCGTGAAAGGGCAGTTTGTGCGTCAAATGCCTAATGAGCCTCAACCTGATTAG |
| CDS_20119_Unigene_33253 | AGAAGAATAAAATATGGATCTATATTTTCAAGAGGTCAATTTCTTCTGTAAATAATAAAGCGACACTACTGAAATATCTTCCTCCCAAGACTAGAAATATCTCAGCATCCCCAAAGACTAACATTGAGAAGACAAAAGAAAACAACAAAATGTGGGCAAACTCTCTTTCGATTCATCAACAATTCTTGAACCCCAACCCTTTCAGAACCCATCAAGATTTCATATTCAAGAATCCCATCTGCTGGAGGAGGCAGAGCTCAGTTTCAGCGCGATGTTGTCTGAAAAAACCAACAGAAAGAAAAAACTATTATGAATTGCTAGGAGTTTCAGTCGATGCGAGTGCTCAAGAAATCAAGCATGCTTACAGAAAACTGCAGAAGAAATATCACCCAGATATAGCTGGTGAAGAGGGTCATGAGAGCACACTCATTTTGAACAAAGCCTACAAGGTATTACTGAGAGATGATCTTAGAAGAGAGTACGACAAATCGATTGGCCAAATTCGGGTAGGGATTGATAGGAGTGTGTTTGGTAGTGTCTGGAAAGAGCCCTTGAGGCCTCAAGCTTTATTTGTCAACGAAAATGCTTGTGTAGGTTGCTGGCAATGCGTGCACCATGCAGGTAATACGTTCACCATGGATGAAGCTTCTGGAACTGCACGGGTCAAGACTCAATATGGTGATGATGATACGCAGATTGAGATGTCGGTCGAGTCATGTCCGGTGAACTGCATCCACTGGGTTGATTCAGAAGAACTAGGAGTGCTTGAGTACCTCATCAGACCTCAGCCAAAGGTGGGATATGGCATATACGGACAAGGATGGGAGAGACCCGCAAATGTATTCATGGCTGCCAAATCCTTCAACAAAGAATTGAAGCGGAAAGAAGAAAGCCAGCAGAGACAGGGAAAATCAAGGGAAGAGGAAGAAACTCCTGCTCAGGCAGAGGCCCGTCAAAATGCATACAAGGAACTGAAGAATGGAAGATTCACCCGATTATGGAGCTGGATGAGGCAAAGCATTAACCAATGA |
| CDS_14745_Unigene_25836 | TGGCATCTTCAGACAAATACCGATCCTTCTTGAACGAAGACGATGTCAAGTACATAAAATGGAGGTATGGTTCCGTTCCCAACTACGACATTGTCAACAAGCTCTTTGAAGAAGGCAGAACCAAGGTATGGCCTCCTGGGTCTCTGGAAGATAAGGTGCAGAATCTTGTGAAGACATGGGAAATGGAGATGTTCCACAAAGTCGATTTCCATGACTATAAATCAGTGGATGCTCACAAGTATAGATTCAGTCTAAATGGAAGGAAAGCATTGTCTCTGGAAGAGAAAAGGAAGCTGGGAGGAGGCTACATCTCATTGCTGCAAACATCCTTACCGGAGAAGTTCAGATGCTTCAATCCAGCAGAGGAGACGGTGGACTCGGCCCACCGGGCTTTCACCACCACGTTTCCTCGAGGGTTCGCCCTGGAGATCCTCGAGGTCTTCTCGGGCCCACCGGTGATCGTCTACAAGTTCAGACACTGGGGGTATATGGAGGGCCCCTTCAAAGGCCATCCACCAACTGGTGAATTGGTGGAGTTCTTTGGAATGTCCATTTTTGAGGTGGATGAGAATTCCAAGATTGTGAGAGTTGAGTTTTTCTACGATCGAGGGGAACTGCTTGGAGCTCTGGTGAAAGGATCAAGCTCTGAAGGTGGTGTTGCTGAAACGTCAGCTTCGGACTGCCCCTTTCTGAGGAACACTGGATAG |
| CDS_11261_Unigene_20868 | TGGAAAGACACTCATCAAAATCCACAACAGTAGCCCTTCTAATTCTCCTAGCCATCATCTCCTCCACCACCAACTCAGGCTGCGCCGCCGCGAGACCCGCAGCCGCCTCCACCAACACCGAGTTCATCCGATCATCCTGCTCCACCACCACCTACCCCACCCTCTGCTACTCCTCCCTCTCCTCCCACGCCGCCCTCATCCAGCAAAACCACAAGCTCCTCGCCGGCACCGCCCTCTCCCTCAGCCTCGACACCGCCCGCTCCACCGCCGGCGACATGGTGAAGCTCTCCCGCTCCGCCGGCATGACGCCGCGCGAGGCCGCCGCCATGCGCGACTGCGTGGAGGTGCTGGCGGACTCCGTGGACGAGCTGAGGAAATCCATGGACGAGATGAAGGGGATGACGGAGTCGAATTTCGGGATGGTGATGAGCGACGTTCAGACGTGGGTGAGCGCCGCCTTGACGGACGAGGACACGTGCATGGATGGGTTCGAGGATAAGGCCGTCAACGGCGGTGTTAAGGCGGCGGTGAGGGGGAAGGTGGTGAATGTGGCGCATATGACTAGTAATGCTTTGGCTCTCATTAATACGTATGCTTCCCTTCATGGTTGA |
