# Supplementary material for: Ocimum metabolomics in response to abiotic stresses: Cold, flood, drought and salinity
Source: PLoS One. 2019 Feb 6;14(2):e0210903. doi: 10.1371/journal.pone.0210903 (PMC6364901; doi:10.1371/journal.pone.0210903)
Supplement: S7 Table — (DOCX) [file pone.0210903.s014.docx]

**S7 Table. Summary of coding sequences (CDS) predicted from unigenes and their annotations.**

|  | **No. of CDS** | **No. of CDS with Blast Hits** | **No. of CDS with No Blast Hits** | **Percent CDS with Blast Hits** | **Percent CDS with No Blast Hits** |
| --- | --- | --- | --- | --- | --- |
| **CONTROL** | 40,560 | 39,073 | 1,487 | 96.33 | 3.67 |
| **COLD** | 32,731 | 31,574 | 1,157 | 96.47 | 3.53 |
| **DROUGHT** | 36,540 | 34,294 | 1,246 | 93.85 | 3.41 |
| **FLOOD** | 34,650 | 33,416 | 1,234 | 96.44 | 3.56 |
| **SALT** | 27,811 | 27,015 | 796 | 97.14 | 2.86 |
