# Supplementary material for: Ocimum metabolomics in response to abiotic stresses: Cold, flood, drought and salinity
Source: PLoS One. 2019 Feb 6;14(2):e0210903. doi: 10.1371/journal.pone.0210903 (PMC6364901; doi:10.1371/journal.pone.0210903)
Supplement: S8 Table — (DOCX) [file pone.0210903.s015.docx]

**S8 Table. GO distribution for CDS.**

| **GO category** | **CONTROL** | **COLD** | **DROUGHT** | **FLOOD** | **SALT** |
| --- | --- | --- | --- | --- | --- |
| **Biological Process** | 4,017 | 3,432 | 3,741 | 2,426 | 3,065 |
| **Cellular Component** | 2,921 | 2,448 | 2,730 | 1,786 | 2,269 |
| **Molecular Function** | 4,731 | 4,015 | 4,409 | 2,894 | 3,552 |
