# Supplementary material for: Ocimum metabolomics in response to abiotic stresses: Cold, flood, drought and salinity
Source: PLoS One. 2019 Feb 6;14(2):e0210903. doi: 10.1371/journal.pone.0210903 (PMC6364901; doi:10.1371/journal.pone.0210903)
Supplement: S1 Fig — (PPTX) [file pone.0210903.s017.pptx]

## Slide 1
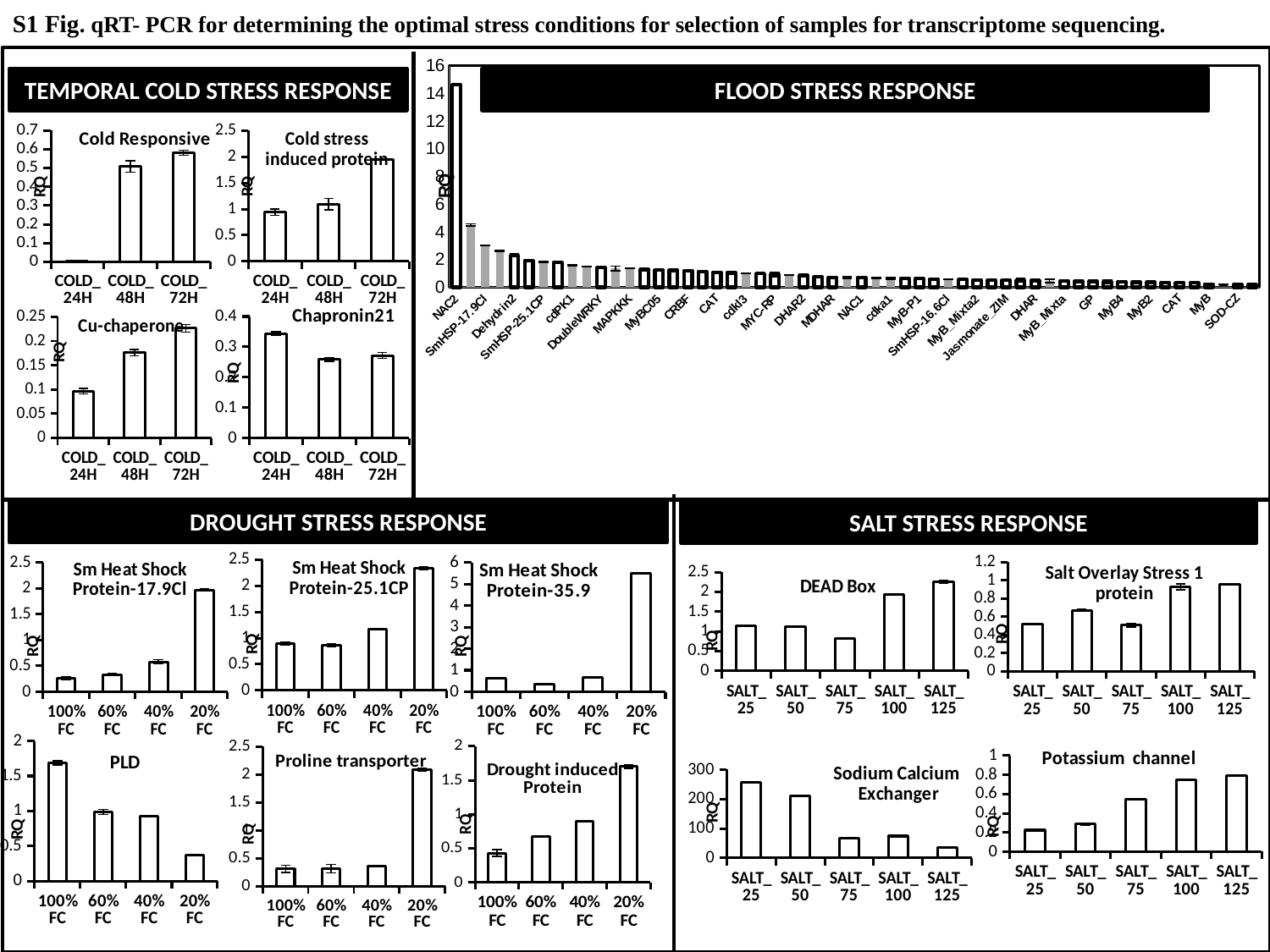

S1 Fig. qRT- PCR for determining the optimal stress conditions for selection of samples for transcriptome sequencing.
TEMPORAL COLD STRESS RESPONSE
### Chart
| Category | |
|---|---|
| NAC2 | 14.612898873833515 |
| SmHSP-17.4BCII | 4.50131959460269 |
| SmHSP-17.9Cl | 3.0065136320993826 |
| SmHSP.4797-5192 | 2.610383005269846 |
| Dehydrin2 | 2.294821595978397 |
| WRKYII | 1.911289079236007 |
| SmHSP-25.1CP | 1.8457370034784597 |
| Dehydrin1 | 1.7854856017474774 |
| cdPK1 | 1.592379275196834 |
| MAPK2 | 1.504898827886691 |
| DoubleWRKY | 1.409475108533898 |
| MAPKK | 1.3611310721446892 |
| MAPKKK | 1.356481620393882 |
| WRKY | 1.2832591078311166 |
| MyBC05 | 1.2527955558901118 |
| MyB1 | 1.2248868625997629 |
| CRBF | 1.1922799304969038 |
| bZIP | 1.127462148734656 |
| CAT | 1.089365059680675 |
| Ferritin | 1.0496286467276597 |
| cdkl3 | 1.0012657940464116 |
| LEA | 0.9889674888748582 |
| MYC-RP | 0.9216628465145391 |
| Dehydration induced Protein | 0.8868853372667117 |
| DHAR2 | 0.854271169909407 |
| COR | 0.7571413154487818 |
| MDHAR | 0.7058732232875676 |
| CaCmPk | 0.6880095520589915 |
| NAC1 | 0.6847871333877716 |
| MAPK | 0.6812203791813275 |
| cdka1 | 0.6464080187159659 |
| GST | 0.6420515320300105 |
| MyB-P1 | 0.6144174092980337 |
| WRKYI | 0.5933365245828639 |
| SmHSP-16.6Cl | 0.5734280965935704 |
| Inducer CBF | 0.5548960736985857 |
| MyB_Mixta2 | 0.5180698388932127 |
| ABA8'hydroxylase | 0.503669578371517 |
| Jasmonate_ZIM | 0.5001135157346867 |
| ERF1 | 0.48701378037426396 |
| DHAR | 0.47979183118822244 |
| SmHSP-35.9 | 0.46111324136359877 |
| MyB_Mixta | 0.45066583858404496 |
| MyC-GP | 0.42725669386192017 |
| GP | 0.426064867606503 |
| AP2 | 0.4021106634087978 |
| MyB4 | 0.37081949563918043 |
| cdkR | 0.36496727124928335 |
| MyB2 | 0.36079356187831246 |
| SOD-Fe | 0.35275550441541476 |
| CAT | 0.33022654501681803 |
| HSP70 | 0.3271116394174983 |
| MyB | 0.23403788649106363 |
| Hypoxia responsive | 0.21036637098567026 |
| SOD-CZ | 0.19573131555194168 |
| SOD-CZ2 | 0.17329014308242355 |FLOOD STRESS RESPONSE
### Chart: Cold Responsive
| Category | COR |
|---|---|
| COLD_24H | 0.0018532631968383594 |
| COLD_48H | 0.5087833979167725 |
| COLD_72H | 0.581037829087742 |
### Chart: Cold stress induced protein
| Category | COLD_Sip |
|---|---|
| COLD_24H | 0.940377874284883 |
| COLD_48H | 1.0946472224251793 |
| COLD_72H | 1.9482934620696106 |
### Chart:
| Category | Cu-chaperone |
|---|---|
| COLD_24H | 0.09675573588259433 |
| COLD_48H | 0.17622157855268292 |
| COLD_72H | 0.22621468628974228 |
### Chart:
| Category | Chapronin21 |
|---|---|
| COLD_24H | 0.3431653990271533 |
| COLD_48H | 0.25857012464564066 |
| COLD_72H | 0.2718006855379585 |DROUGHT STRESS RESPONSE
SALT STRESS RESPONSE
### Chart: Sm Heat Shock Protein-25.1CP
| Category | SmHSP-25.1CP |
|---|---|
| 100% FC | 0.8973993225303476 |
| 60% FC | 0.861651866753927 |
| 40% FC | 1.178476508092637 |
| 20% FC | 2.3307592620294653 |
### Chart: Sm Heat Shock Protein-35.9
| Category | SmHSP-35.9 |
|---|---|
| 100% FC | 0.6246933165342111 |
| 60% FC | 0.3549025880278781 |
| 40% FC | 0.6553547644077906 |
| 20% FC | 5.512002338063148 |
### Chart: Sm Heat Shock Protein-17.9Cl
| Category | SmHSP-17.9Cl |
|---|---|
| 100% FC | 0.2629917594286026 |
| 60% FC | 0.33478400339871467 |
| 40% FC | 0.580004500549567 |
| 20% FC | 1.9715721403397242 |
### Chart: Salt Overlay Stress 1 protein
| Category | SOS1 |
|---|---|
| SALT_25 | 0.52037047022106 |
| SALT_50 | 0.669367077678386 |
| SALT_75 | 0.5066367025966767 |
| SALT_100 | 0.9298078428302216 |
| SALT_125 | 0.9565511357501575 |
### Chart:
| Category | DEAD Box |
|---|---|
| SALT_25 | 1.137906879764068 |
| SALT_50 | 1.1101853034119014 |
| SALT_75 | 0.8202090117755516 |
| SALT_100 | 1.926294786634577 |
| SALT_125 | 2.2479119083087578 |
### Chart:
| Category | PLD |
|---|---|
| 100% FC | 1.685965482893545 |
| 60% FC | 0.9869846140359937 |
| 40% FC | 0.9283685146248108 |
| 20% FC | 0.3709218086813959 |
### Chart: Proline transporter
| Category | Pro.transporter |
|---|---|
| 100% FC | 0.31433536500044595 |
| 60% FC | 0.31659985503379334 |
| 40% FC | 0.36027763467642426 |
| 20% FC | 2.0890803756811636 |
### Chart: Drought induced Protein
| Category | Drought_indP |
|---|---|
| 100% FC | 0.43091672517973933 |
| 60% FC | 0.6728636080753516 |
| 40% FC | 0.8998961961757507 |
| 20% FC | 1.70335778447033 |
### Chart: Potassium channel
| Category | K.channel |
|---|---|
| SALT_25 | 0.22416423154736848 |
| SALT_50 | 0.28849105655121016 |
| SALT_75 | 0.5414237203286636 |
| SALT_100 | 0.7465816734769336 |
| SALT_125 | 0.7926305617402472 |
### Chart: Sodium Calcium
Exchanger
| Category | NaCa.Exchanger |
|---|---|
| SALT_25 | 256.54115943346545 |
| SALT_50 | 209.82263362593096 |
| SALT_75 | 68.3790986182856 |
| SALT_100 | 74.34310295565429 |
| SALT_125 | 35.44449082134319 |
