# Supplementary material for: Ocimum metabolomics in response to abiotic stresses: Cold, flood, drought and salinity
Source: PLoS One. 2019 Feb 6;14(2):e0210903. doi: 10.1371/journal.pone.0210903 (PMC6364901; doi:10.1371/journal.pone.0210903)
Supplement: S2 Fig — (A) Cold, (B) Flood, (C) Drought, (D) Salt, and (E) Control. Blue bar = Unigene, Red bar = CDS. (PPTX) [file pone.0210903.s018.pptx]

## Slide 1
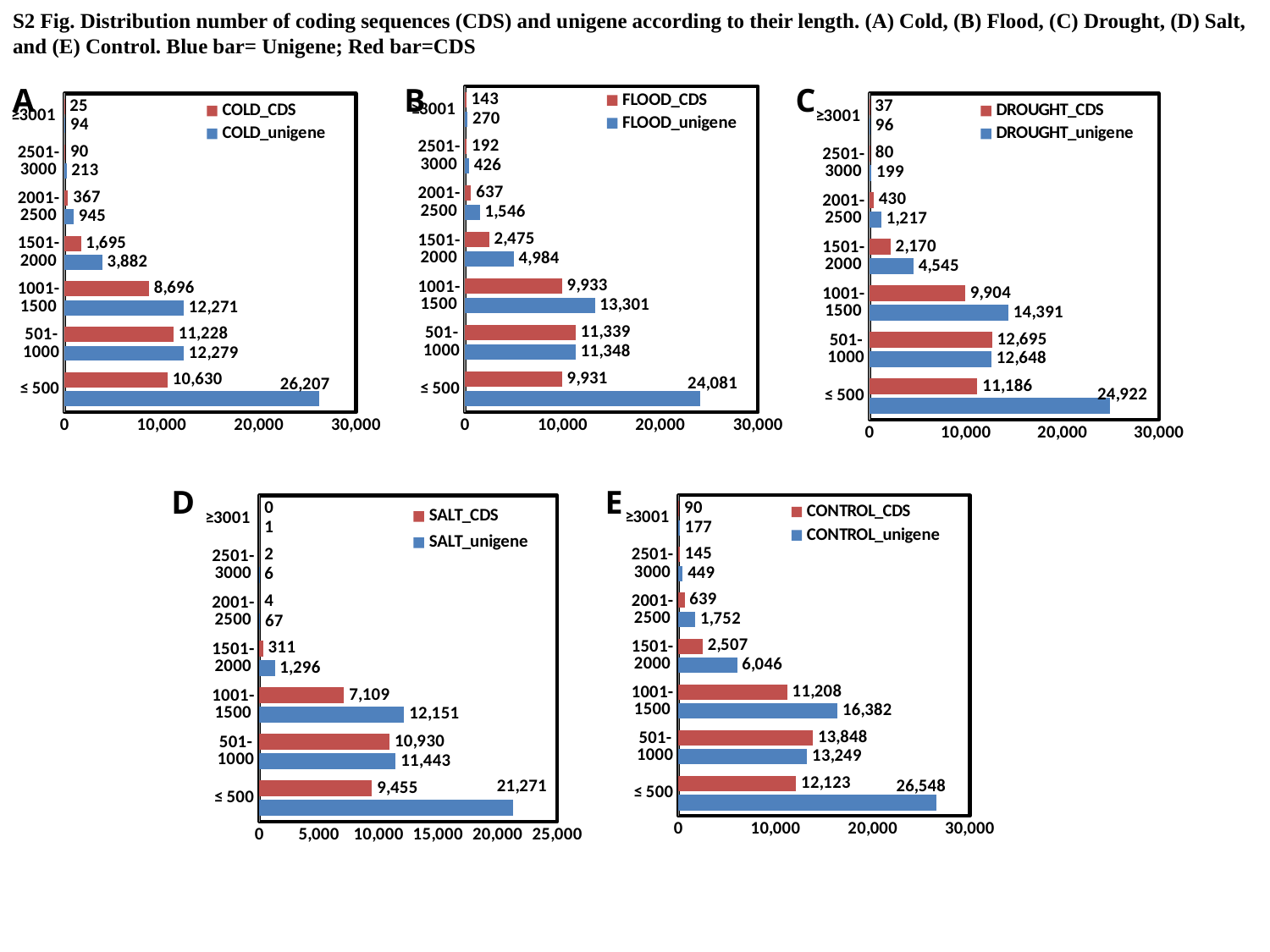

S2 Fig. Distribution number of coding sequences (CDS) and unigene according to their length. (A) Cold, (B) Flood, (C) Drought, (D) Salt, and (E) Control. Blue bar= Unigene; Red bar=CDS
### Chart
| Category | COLD_unigene | COLD_CDS |
|---|---|---|
| ≤ 500 | 26207.0 | 10630.0 |
| 501- 1000 | 12279.0 | 11228.0 |
| 1001- 1500 | 12271.0 | 8696.0 |
| 1501- 2000 | 3882.0 | 1695.0 |
| 2001- 2500 | 945.0 | 367.0 |
| 2501- 3000 | 213.0 | 90.0 |
| ≥3001 | 94.0 | 25.0 |A
B
C
### Chart
| Category | DROUGHT_unigene | DROUGHT_CDS |
|---|---|---|
| ≤ 500 | 24922.0 | 11186.0 |
| 501- 1000 | 12648.0 | 12695.0 |
| 1001- 1500 | 14391.0 | 9904.0 |
| 1501- 2000 | 4545.0 | 2170.0 |
| 2001- 2500 | 1217.0 | 430.0 |
| 2501- 3000 | 199.0 | 80.0 |
| ≥3001 | 96.0 | 37.0 |
### Chart
| Category | FLOOD_unigene | FLOOD_CDS |
|---|---|---|
| ≤ 500 | 24081.0 | 9931.0 |
| 501- 1000 | 11348.0 | 11339.0 |
| 1001- 1500 | 13301.0 | 9933.0 |
| 1501- 2000 | 4984.0 | 2475.0 |
| 2001- 2500 | 1546.0 | 637.0 |
| 2501- 3000 | 426.0 | 192.0 |
| ≥3001 | 270.0 | 143.0 |D
### Chart
| Category | SALT_unigene | SALT_CDS |
|---|---|---|
| ≤ 500 | 21271.0 | 9455.0 |
| 501- 1000 | 11443.0 | 10930.0 |
| 1001- 1500 | 12151.0 | 7109.0 |
| 1501- 2000 | 1296.0 | 311.0 |
| 2001- 2500 | 67.0 | 4.0 |
| 2501- 3000 | 6.0 | 2.0 |
| ≥3001 | 1.0 | 0.0 |E
### Chart
| Category | CONTROL_unigene | CONTROL_CDS |
|---|---|---|
| ≤ 500 | 26548.0 | 12123.0 |
| 501- 1000 | 13249.0 | 13848.0 |
| 1001- 1500 | 16382.0 | 11208.0 |
| 1501- 2000 | 6046.0 | 2507.0 |
| 2001- 2500 | 1752.0 | 639.0 |
| 2501- 3000 | 449.0 | 145.0 |
| ≥3001 | 177.0 | 90.0 |
