# Supplementary material for: Ocimum metabolomics in response to abiotic stresses: Cold, flood, drought and salinity
Source: PLoS One. 2019 Feb 6;14(2):e0210903. doi: 10.1371/journal.pone.0210903 (PMC6364901; doi:10.1371/journal.pone.0210903)
Supplement: S3 Fig — (PPTX) [file pone.0210903.s019.pptx]

## Slide 1
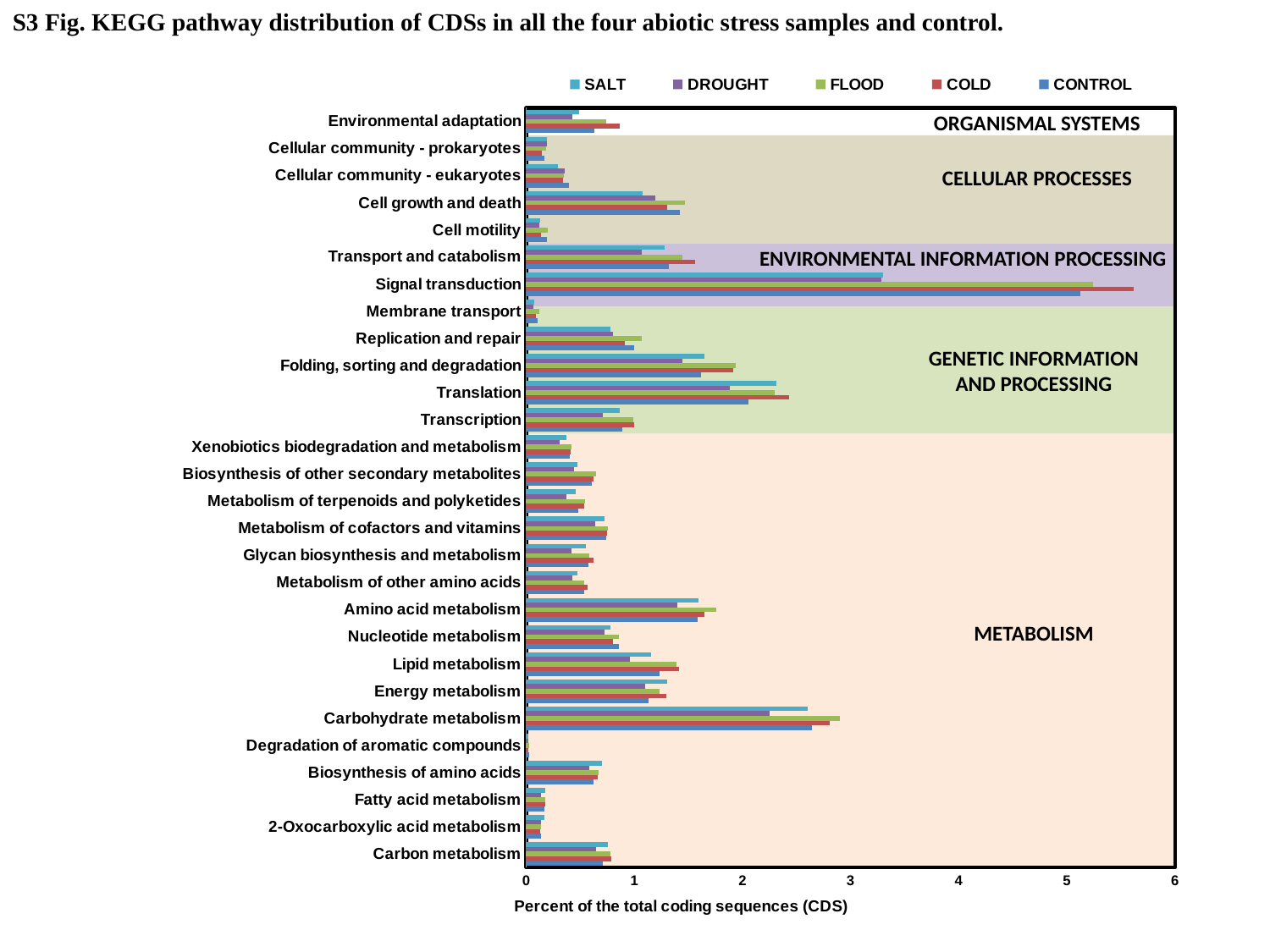

S3 Fig. KEGG pathway distribution of CDSs in all the four abiotic stress samples and control.
### Chart
| Category | CONTROL | COLD | FLOOD | DROUGHT | SALT |
|---|---|---|---|---|---|
| Carbon metabolism | 0.7100591715976331 | 0.7882435611499801 | 0.7763347763347787 | 0.6465399156210618 | 0.7586926036460424 |
| 2-Oxocarboxylic acid metabolism | 0.13806706114398423 | 0.12831871925697352 | 0.13564213564213626 | 0.13423921976877978 | 0.16899787853727066 |
| Fatty acid metabolism | 0.17011834319526725 | 0.1741468332773222 | 0.17604617604617662 | 0.13697879568242913 | 0.17978497716730854 |
| Biosynthesis of amino acids | 0.6262327416173548 | 0.659924841893009 | 0.6666666666666667 | 0.5862692455207936 | 0.7011614109525008 |
| Degradation of aromatic compounds | 0.027120315581854265 | 0.01833124560813913 | 0.02886002886002887 | 0.019177031395539983 | 0.017978497716730848 |
| Carbohydrate metabolism | 2.6454635108481264 | 2.807735785646641 | 2.9004329004329006 | 2.251931401019123 | 2.6068821689259645 |
| Energy metabolism | 1.1341222879684418 | 1.298463230576518 | 1.22943722943723 | 1.101309517286724 | 1.30164323469131 |
| Lipid metabolism | 1.2352071005917205 | 1.4145611194280652 | 1.391053391053391 | 0.9561119938633499 | 1.1542195534141166 |
| Nucleotide metabolism | 0.8579881656804736 | 0.8065748067581207 | 0.8571428571428577 | 0.7232480412032217 | 0.7766711013627716 |
| Amino acid metabolism | 1.5877712031558178 | 1.6498121047325216 | 1.7604617604617643 | 1.39718371596077 | 1.5964905972456938 |
| Metabolism of other amino acids | 0.5350098619329366 | 0.5652134062509545 | 0.5367965367965368 | 0.42463426661552794 | 0.4746323397216929 |
| Glycan biosynthesis and metabolism | 0.5769230769230806 | 0.6232623506767285 | 0.5829725829725827 | 0.41915511478823075 | 0.5537377296753083 |
| Metabolism of cofactors and vitamins | 0.7371794871794916 | 0.745470654730989 | 0.7561327561327561 | 0.6355816119664677 | 0.7263313077559236 |
| Metabolism of terpenoids and polyketides | 0.4807692307692308 | 0.5346613302373896 | 0.5454545454545455 | 0.37258232425620647 | 0.46024954154830705 |
| Biosynthesis of other secondary metabolites | 0.606508875739645 | 0.6232623506767285 | 0.6464646464646466 | 0.44381129801106844 | 0.4746323397216929 |
| Xenobiotics biodegradation and metabolism | 0.4068047337278129 | 0.40939781858177265 | 0.42135642135642315 | 0.3123116541559368 | 0.37035705296465604 |
| Transcription | 0.8875739644970404 | 1.0021080932449358 | 0.9898989898989895 | 0.7095501616349789 | 0.8629678904030815 |
| Translation | 2.0586785009861934 | 2.428890043078427 | 2.3001443001443 | 1.8848282285902143 | 2.3120348063715794 |
| Folding, sorting and degradation | 1.6173570019723906 | 1.915615166050536 | 1.93939393939394 | 1.446496082406439 | 1.6504260903958858 |
| Replication and repair | 1.0009861932938857 | 0.9135070728055976 | 1.064935064935069 | 0.8054353186126788 | 0.7802668009061143 |
| Membrane transport | 0.10355029585798817 | 0.09165622804069588 | 0.11832611832611874 | 0.06574982192756586 | 0.07550969041026932 |
| Signal transduction | 5.120808678500963 | 5.6154715712932655 | 5.2380952380952355 | 3.284751520464641 | 3.297256481248427 |
| Transport and catabolism | 1.3165680473372778 | 1.5642662918945338 | 1.443001443001439 | 1.0684346063229373 | 1.2836647369745744 |
| Cell motility | 0.1898422090729792 | 0.13748434206104376 | 0.19624819624819712 | 0.12328091611418551 | 0.1294451835604632 |
| Cell growth and death | 1.4176528599605522 | 1.3015184381778742 | 1.4660894660894659 | 1.191715522437128 | 1.078709863003848 |
| Cellular community - eukaryotes | 0.39201183431952796 | 0.34218325135193 | 0.3463203463203482 | 0.35340529286066646 | 0.294847362554385 |
| Cellular community - prokaryotes | 0.16765285996055188 | 0.14359475726375587 | 0.18181818181818263 | 0.19450988986904877 | 0.19416777534069254 |
| Environmental adaptation | 0.631163708086785 | 0.8646237511838973 | 0.7417027417027394 | 0.4273738425291765 | 0.4926108374384238 |ORGANISMAL SYSTEMS
CELLULAR PROCESSES
ENVIRONMENTAL INFORMATION PROCESSING
GENETIC INFORMATION AND PROCESSING
METABOLISM
