# Supplementary figures and images for: Ocimum metabolomics in response to abiotic stresses: Cold, flood, drought and salinity
Source: PLoS One. 2019 Feb 6;14(2):e0210903. doi: 10.1371/journal.pone.0210903 (PMC6364901; doi:10.1371/journal.pone.0210903)

## Slide 1
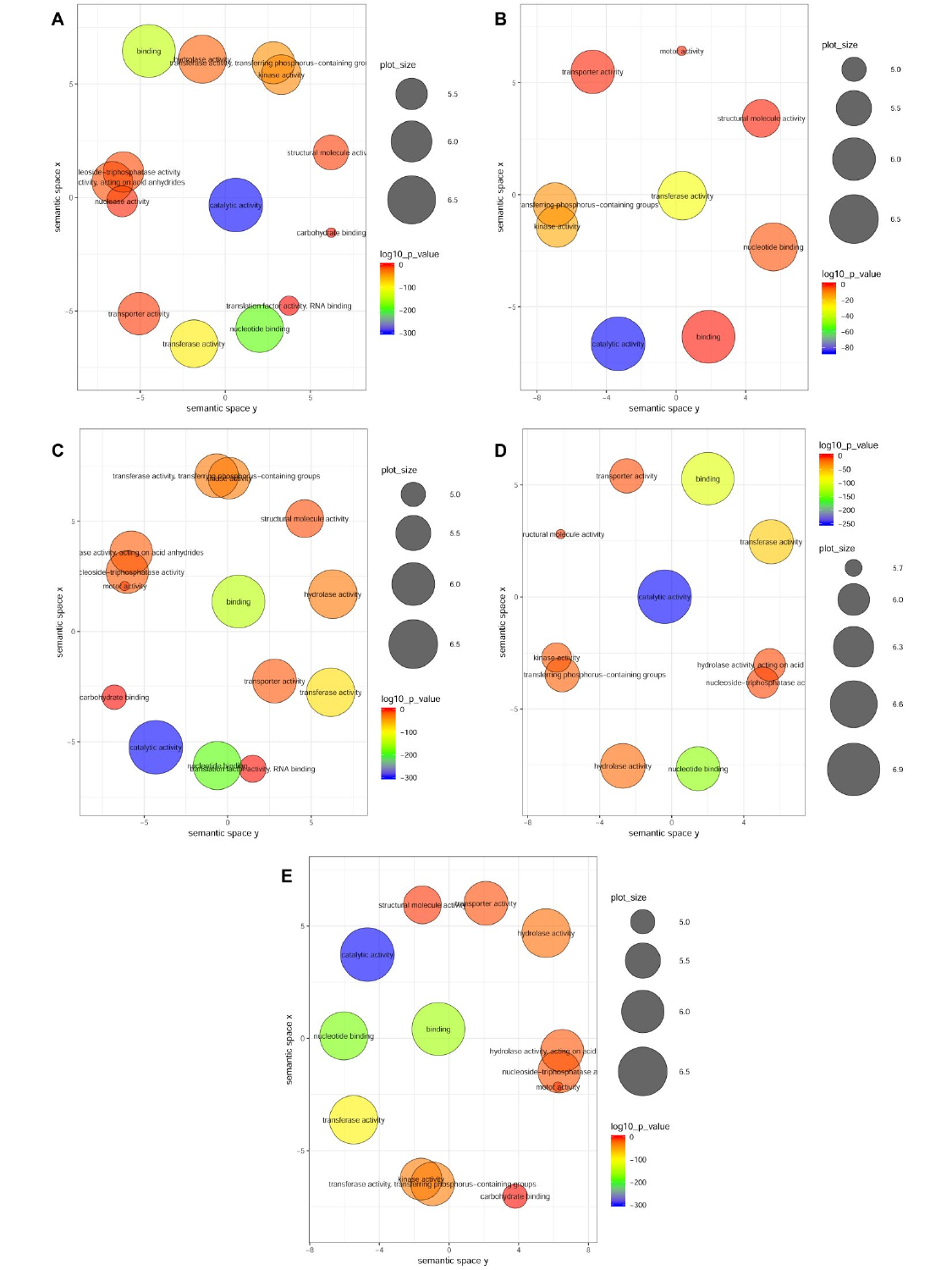

Supplement: S4 Fig — (A) Significantly enriched transcripts in MF GO terms related to cold treatment. (B) Significantly enriched transcripts in MF GO terms related to flood treatment. (C) Significantly enriched transcripts in MF GO terms related to drought treatment. (D) Significantly enriched transcripts in MF GO terms related to salt treatment. (E) Significantly enriched transcripts in MF GO terms related to control. Circles indicate the GO terms (Plant GO slims) clustered on the basis of semantic identities to other GO terms in ontology (larger circles depict more general terms, while adjacent circles are strongly related). Size of the circle is relative to the GO term frequency where the colors indicate the enrichment in form of log10P-value from the AgriGO results (blue lower, red higher). (PPTX) [file pone.0210903.s020.pptx]
